# Beta-hydroxy beta-methylbutyrate/arginine/glutamine (HMB/Arg/Gln) supplementation to improve the management of cachexia in patients with advanced lung cancer: an open-label, multicentre, randomised, controlled phase II trial (NOURISH)

**DOI:** 10.1186/s12885-021-08519-8

**Published:** 2021-07-12

**Authors:** Jennifer Pascoe, Aimee Jackson, Charlotte Gaskell, Claire Gaunt, Joyce Thompson, Lucinda Billingham, Neil Steven

**Affiliations:** 1 Queen Elizabeth Hospital Birmingham, Mindelsohn Way, Birmingham, B15 2TH UK; 2 Cancer Research UK Clinical Trials Unit (CRCTU), University of Birmingham, Edgbaston, Birmingham, B15 2TT UK; 3 Birmingham Heartlands Hospital, Bordesley Green E, Birmingham, B9 5SS UK; 4 Institute of Immunology and Immunotherapy, College of Medical and Dental Sciences, University of Birmingham, Edgbaston, Birmingham, B15 2TT UK

**Keywords:** Cachexia, Advanced lung cancer, Nutritional supplement, Supportive care, Clinical trial

## Abstract

**Background:**

Cancer cachexia causes significant morbidity and mortality in advanced lung cancer patients. Clinical benefit of β-hydroxy-β-methylbutyrate, arginine, and glutamine (HMB/Arg/Gln) was assessed in newly diagnosed patients.

**Methods:**

NOURISH, a prospective, two-arm, open-label, multi-centre, randomised controlled phase II trial compared cachexia in patients who received HMB/Arg/Gln with those who did not. All patients received structured nutritional, exercise and symptom control via a Macmillan Durham Cachexia Pack. Conducted in five UK centres, patients aged > 18 years, with newly diagnosed advanced small cell lung cancer (SCLC) or non-small cell lung cancer (NSCLC), who were able to take oral nutrition, with a performance status of 0-to-2 and a life expectancy > 4 months were eligible for trial entry. Patients suitable for treatment with curative intent were ineligible. The trial was designed as a signal-seeking pilot study with target recruitment of 96 patients. One-to-one randomisation was stratified by diagnosis (SCLC or NSCLC), stage of disease (locally advanced or metastatic) and performance status. The primary outcome measure was treatment success defined as a patient being alive without significant loss of lean body mass (not > 5%) by 12 weeks. Secondary outcome measures included quality of life.

**Results:**

Between February-2012 and February-2013, 38 patients were recruited, 19 to each arm. Baseline characteristics were balanced. The trial was halted due to slow accrual and partial adherence. Trial data demonstrated no evidence of treatment benefit. No serious adverse events were reported during the trial.

**Conclusions:**

Further evaluation of HMB/Arg/Gln in this setting could not be recommended on the basis of this trial.

**Clinical trial registration:**

ISRCTN registry: 39911673; 14-Apr-2011 10.1186/ISRCTN39911673.

**Supplementary Information:**

The online version contains supplementary material available at 10.1186/s12885-021-08519-8.

## Background

Cancer cachexia results in significant morbidity and mortality. It is very common among patients with advanced lung cancer, with an estimated incidence of between 36 and 76% [1–3], and its presence is associated with worse outcomes [4, 5].

Patients with cancer cachexia experience a number of distressing symptoms, functional impairment and decreased tolerance of cancer treatment [6, 7]. Cancer cachexia is described as a multifactorial syndrome defined by an ongoing loss of skeletal muscle mass (with or without loss of fat mass) that cannot be fully reversed by conventional nutritional support and leads to progressive functional impairment [4].

In recent years there has been a significant increase in research in the field of cachexia resulting in greater understanding of pathophysiology and an appreciation that cancer cachexia represents a continuum of pre-cachexia, cachexia and refractory cachexia [4, 8]. Although its pathophysiology remains incompletely understood, it is known to be multifactorial in nature and characterised by a negative protein and energy balance and abnormal metabolism [4, 9]. A number of different pathways have been associated with, and contribute to, the pathogenesis of cancer cachexia including the secretion of inflammatory cytokines such as tumour necrosis factor-α (TNFα), proteolysis-inducing factor (PIF), lipolysis and lipid-mobilising factor (LMF), as well as abnormalities in glucose, fat and protein metabolism, and abnormalities in mitochondrial energy metabolism which contribute to tissue catabolism, all promoting cancer cachexia [8].

This increased understanding of the pathology behind the development of cachexia has led to some promising new angles of investigation of potential therapeutic agents. It has long been recognised that cancer cachexia cannot be reversed by nutritional support alone [9]. However, despite a large number of randomised clinical trials of investigational agents including, amongst others, progestins [10], cannabinoids [11], corticosteroids [12], non-steroidal anti-inflammatory drugs (NSAIDs) [13] and thalidomide [14], there is currently no effective treatment for cancer cachexia in clinical use. It is possible that this, in part, is because clinical trials of investigational agents for cancer cachexia often recruit patients with very advanced disease or refractory cachexia. These patients have severe muscle wasting, catabolism and a low performance status and are unlikely to benefit from any cachexia therapy. This frequently results in poor recruitment and high dropout rates. In this situation, it is possible that a potentially effective agent has been unable to demonstrate clinical efficacy due to trial design. One promising agent has recently emerged however, anamorelin, which has demonstrated benefit in patients with advanced non-small cell lung cancer (NSCLC)-associated cachexia [15–17], although it still remains to be adopted into routine clinical care.

At the time of the NOURISH trial’s inception, an agent with a strong biological rationale for use in cancer cachexia was β-hydroxy β-methyl butyrate (HMB) in combination with arginine and glutamine (HMB/Arg/Gln). The oral nutritional supplement was initially reported to improve wound healing via improved protein and collagen synthesis [18]. β-hydroxy β-methyl butyrate is an active metabolite of the amino acid leucine that may improve muscle protein turnover [19]. Arginine may synergise with HMB to attenuate muscle loss [20], with studies suggesting glutamine can upregulate muscle protein synthesis [21]. All three components of this amino acid rich supplement may work together to decrease muscle damage from reactive oxygen species and pro-inflammatory cytokines [20–22]. Of relevance to NOURISH, clinical studies suggested HMB/Arg/Gln supports maintenance of lean body mass (LBM) in older, healthy adults [23]. A large randomised trial of 472 patients with advanced cancer and who experienced 2–10% weight loss were given HMB/Arg/Gln or placebo for 8 weeks [24]. Although, no significant difference in LBM was observed at the end of treatment, a trend towards higher LBM in the intervention arm compared to placebo was noted. These data supported an earlier smaller randomised trial where 49 patients with advanced cancer and weight loss greater than 5% were administered HMB/Arg/Gln or control [25]; a significant increase in fat-free mass (FFM) in the intervention arm was observed (1.6 kg +/− 0.94; *P* < 0.05). Both trials however experienced a high dropout rate; only 37 and 18% completing the trial, respectively. Therefore, further investigation specifically in advanced lung cancer was warranted.

We postulated that to test the effectiveness of an intervention it not only needed to be given early in the disease process before the onset of refractory cachexia but also in conjunction with attention to nutritional support, exercise support, symptom control and appropriately targeted anticancer therapy. It was important that this supportive therapy was deliverable within everyday clinical practice. We identified the Macmillan Durham Cachexia Pack (MDCP) as a vehicle with which to deliver a standardised symptom control programme [26, 27]. The MDCP was a resource developed in 2007 by a Durham-based team with support from professionals around the UK. It provided an evidence-based guide for healthcare professionals to assess and manage common symptoms and problems seen in patients with anorexia-cachexia syndrome. The pack also contained a number of leaflets to help patients and their families deal with the emotional and psychological impact of the condition, however, efficacy of the packs use by clinicians remains unpublished. The MDCP used during the NOURISH trial has been included in Supplementary Appendix 1.

The NOURISH trial was, therefore, a randomised phase II trial designed as a pilot to detect a signal that dietary supplementation with HMB/Arg/Gln, on a background of structured nutritional and symptom support, delays the onset of cachexia in patients with advanced lung cancer sufficiently to justify further investigation in a larger phase III trial. Unlike previous trials before it, patients recruited into NOURISH were not required to have weight loss or other symptoms of cachexia. This paper reports the results from the NOURISH trial, which despite the limited data, can still contribute to the pool of evidence in this important clinical area.

## Methods

### Study design

The NOURISH trial was a multicentre, open label, two-arm, randomised controlled phase II clinical trial recruiting patients from five hospitals in the United Kingdom.

### Patients

Patients with newly diagnosed advanced small cell lung cancer (SCLC) or NSCLC who were able to take oral nutrition with a performance status of 0 to 2 and a life expectancy greater than 4 months were eligible for this trial. Patients who were suitable for radical treatment with curative intent, and/or patients who had already commenced first line chemotherapy or radiotherapy, and/or those in whom the diagnosis of lung cancer was made more than 8 weeks previously, were not eligible for trial entry.

### Randomisation

Eligible patients were randomly assigned on a 1:1 basis to receive the HMB/Arg/Gln nutritional supplement or not. Treatment allocation was by a computerised minimisation algorithm, accessed by investigators via telephone, which was developed and run by the Cancer Research UK Clinical Trials Unit (CRCTU) at the University of Birmingham. Randomisation was stratified by diagnosis (SCLC or NSCLC), stage of disease (locally advanced or metastatic) and WHO performance status (0, 1 or 2). These were balanced across the treatment groups.

### Procedures

Patients were randomised to receive either the experimental arm of HMB/Arg/Gln (one sachet twice daily) for 12 weeks or until intolerable, or the control arm of no HMB/Arg/Gln. Each sachet contained HMB 1.2 g, arginine 7 g, glutamine 7 g and was 78 cal and was dissolved in 240-300 ml of cold water or juice.

**Fig. 1:**
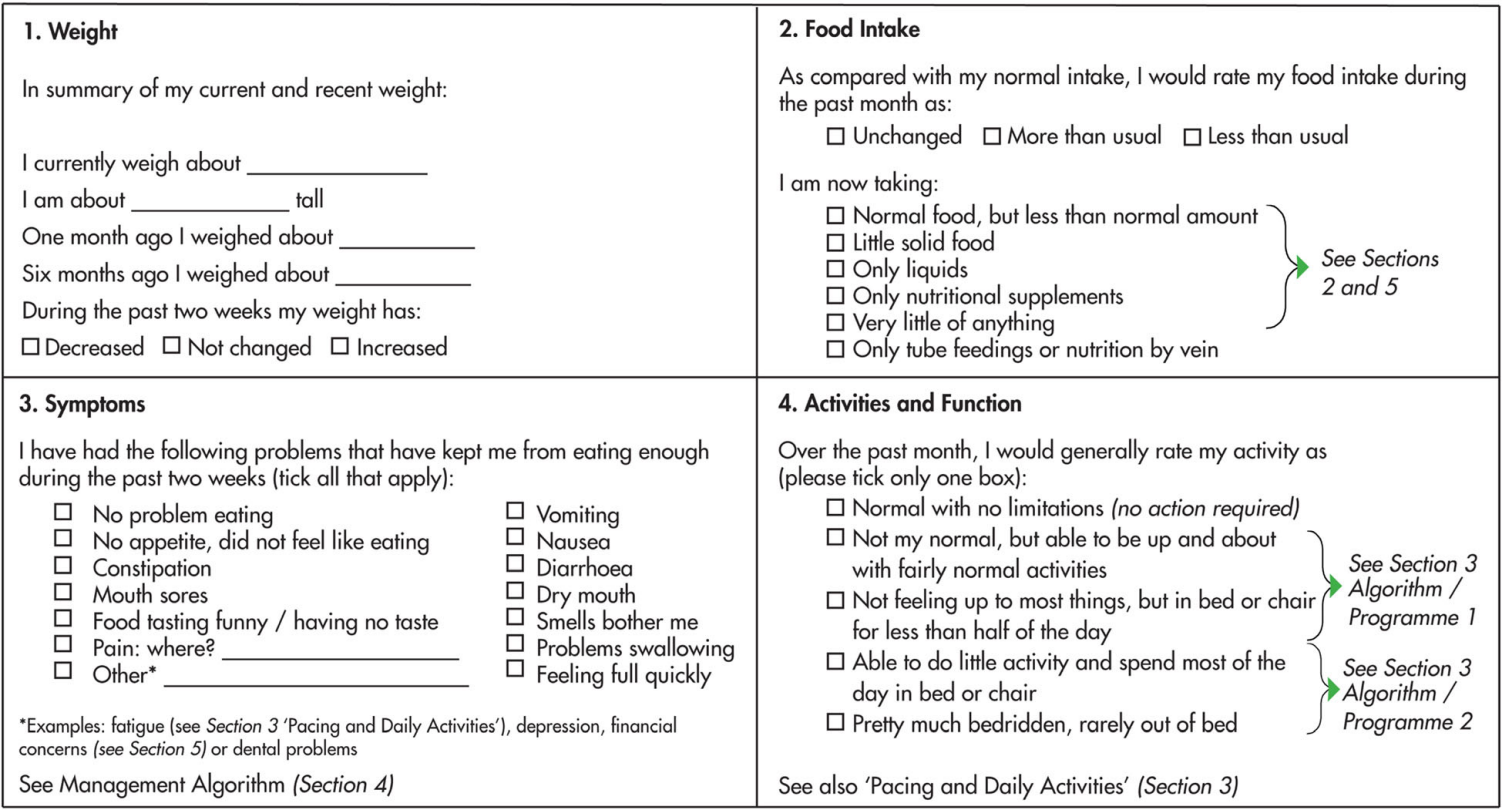
Abridged Patient-Generated Subjective Global Assessment. Taken from the Macmillan Durham Cachexia Pack, 2007 (Supplementary Appendix 1)

All patients received structured nutritional, exercise and symptom control advice through use of the MDCP at each trial visit [26]. Patients completed the Patient Generated Subjective Global Assessment (PG-SGA) [28] contained within the MDCP, which was reviewed by a member of the research team who then offered appropriate advice and/or interventions as guided by the MDCP. As specified within the MDCP, an abridged PG-SGA was then completed by patients at each trial visit (Fig. 1).

All patients received treatment for their underlying condition as felt appropriate by their oncologist. This could include palliative chemotherapy, radiotherapy or active symptom control.

Study visits were conducted at six time points during the trial; baseline, 3-, 6-, 9- and 12-weeks during treatment, with a final visit taking place 6 weeks after completion of the trial intervention. At each visit, measurement of LBM was performed by bioelectrical impedance analysis (BIA) using a bioelectrical impedance leg-to-leg analyser. In addition, handgrip strength was measured using the Jamer™ dynamometer.

The Functional Assessment of Anorexia Cachexia Therapy (FAACT) questionnaire [29], was administered by research nurses at baseline and week 12 visits to assess quality of life (QoL). The questionnaire was completed independently by patients.

### Outcomes

The primary outcome measure was treatment success defined as a patient being alive without significant loss of LBM (not more than 5%) by 12 weeks.

Secondary outcomes consisted of change in LBM measured from baseline to week 12, LBM at 3-weekly intervals from start of treatment intervention for 12 weeks, functional status assessed by handgrip strength across trial visits and change in FAACT QoL score between baseline and week 12.

### Statistical analysis

The statistical design was based on the binary primary outcome measure of treatment success, as defined above, and used an extension of Simon’s two-stage design for single arm phase II trials, described by Jung et al., [30] . Assuming a treatment success rate of 40% on the control arm and taking a relaxed significance level of 0.2, appropriate for a signal-seeking pilot phase II trial, it was determined a sample size of 48 patients per arm has power of 0.85 to detect an absolute improvement in the treatment success rate of 20% on the experimental arm i.e. improvement to 60%. Therefore, the trial aimed to recruit 96 patients randomised in a 1:1 ratio between the two arms and if the number of treatment successes on the experimental arm was greater than or equal to five then it would be deemed sufficiently beneficial to warrant further investigation in a larger phase III trial.

As stipulated in the protocol, a Data Monitoring Committee (DMC) was not planned for this short-term, phase II trial. However, an interim analysis was scheduled to take place when recruitment had reached 50%, at which point trial data would be reviewed by an independent statistician to assess progress and give advice on whether the accumulated data from the trial, together with the results from other relevant trials, justified continued recruitment. There were no formal stopping rules.

With the trial not reaching its target recruitment, primary outcome analysis based on the Jung design was not possible. Therefore, trial treatment arms were compared in terms of treatment success rate using an odds ratio with 95% confidence interval, with estimates based on the intention-to-treat principle.

For secondary outcomes, descriptive analysis was used to report the change in LBM, handgrip strength and QoL over time.

All statistical analyses were performed using SAS version 9.3.

The trial was registered on ISRCTN: 39911673.

## Results

**Fig. 2:**
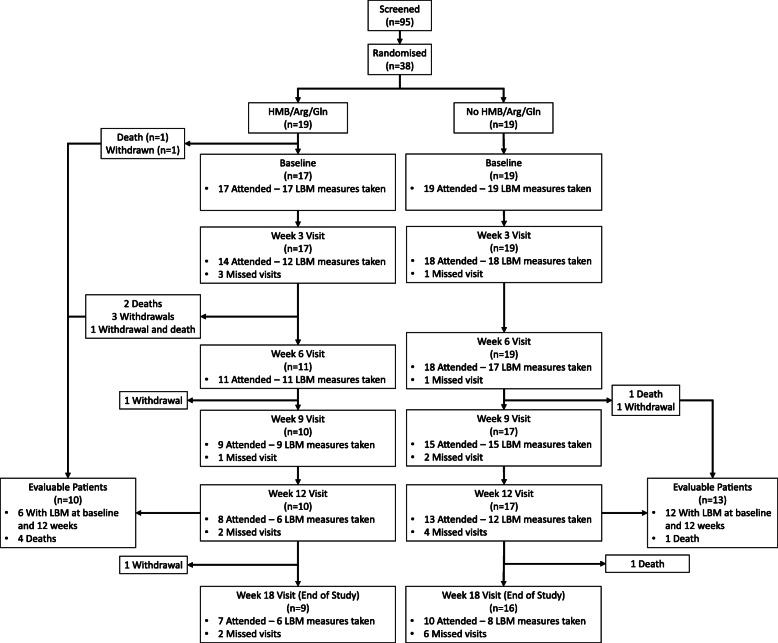
NOURISH trial profile. LBM, lean body mass

Between February-2012 and February-2013, 95 patients were screened for the trial of which, 38 patients were randomised; 19 to HMB/Arg/Gln and 19 to no HMB/Arg/Gln (Fig. 2). Collection and analysis of patient screening logs revealed the common reasons patients failed eligibility included; greater than 8 weeks from diagnosis, poor performance status, entry into other treatment trials and patients’ unwillingness to attend the extra hospital visits required.

In February 2013, there was a temporary halt to recruitment due to concerns over the lack of adherence and early discontinuation from treatment, together with patient withdrawals and deaths and an interim analysis was initiated. A futility analysis was requested by the external independent statistician to determine the probability a significant result would be observed in favour of the experimental arm if the trial was to continue recruiting. The results demonstrated a < 1% chance of a positive outcome being observed. It was therefore recommended by the external independent statistician the trial be discontinued, which was agreed by the Trial Management Group in December-2013 and the trial was closed to recruitment with 38 patients included.

Patient characteristics and stratification variables at baseline were well balanced across the treatment arms (Table 1). Of the 38 patients randomised, 68% were aged over 60, 61% male, and 76% had performance status of 1 or more. The majority of patients randomised were diagnosed with NSCLC (84%) with a large number of patients having metastatic disease (63%). NOURISH was designed as a pragmatic study with minimal data collection so details about the primary cancer treatment being received by the patients during the trial were not collected, but these patients would typically have been receiving palliative chemotherapy such as gemcitabine and carboplatin, or carboplatin and etoposide.

**Table 1:** Baseline patient characteristics

		HMB/Arg/Gln *n* = 19 N (%)	No HMB/Arg/Gln *n* = 19 N (%)	All *n* = 38 N (%)
**Age**	60 or below	6 (32)	6 (32)	12 (32)
	Over 60	13 (68)	13 (68)	26 (68)
**Sex**	Male	12 (63)	11 (58)	23 (61)
	Female	7 (37)	8 (42)	15 (39)
**Diagnosis**	SCLC	3 (16)	3 (16)	6 (16)
	NSCLC	16 (84)	16 (84)	32 (84)
**Staging**^a^	Locally advanced	8 (42)	6 (32)	14 (37)
	Metastatic	11 (58)	13 (68)	24 (63)
**WHO Performance status**	0	2 (10.5)	7 (37)	9 (24)
	1	15 (79)	11 (58)	26 (68)
	2	2 (10.5)	1 (5)	3 (8)

^a^Correlative staging has been added retrospectively for information, but was not collected at the time of the trial: Locally advanced = Stage 3B NSCLC; Metastatic = Stage 4 NSCLC and extensive stage SCLC

*SCLC* small-cell lung cancer, *NSCLC* non-small-cell lung cancer, *HMB/Arg/Gln* β-Hydroxy β-Methylbutyrate/Arginine/Glutamine

Of the 38 patients randomised, one withdrew and one died prior to the baseline visit on the HMB/Arg/Gln arm (Fig. 2). All 36 patients who attended their baseline visit completed the PG-SGA contained within the MDCP. As a result, 14 patients received advice at this time, 4 randomised to receive HMB/Arg/Gln and 10 randomised to the control arm. The main interventions given were verbal advice (n = 5) , exercise advice (n = 4) , and verbal advice with a dietary referral (n = 2). Data were collected following completion of an abridged PG-SGA within the MDCP from 96 of the 106 subsequent trial visits on weeks 3, 6, 9 and 12. Sixteen interventions were made, five to patients randomised to receive HMG/Arg/Gln and eleven to not receiving HMG/Arg/Gln. The most common interventions were exercise advice, and verbal advice, each made six times during the subsequent visits.

**Fig. 3:**
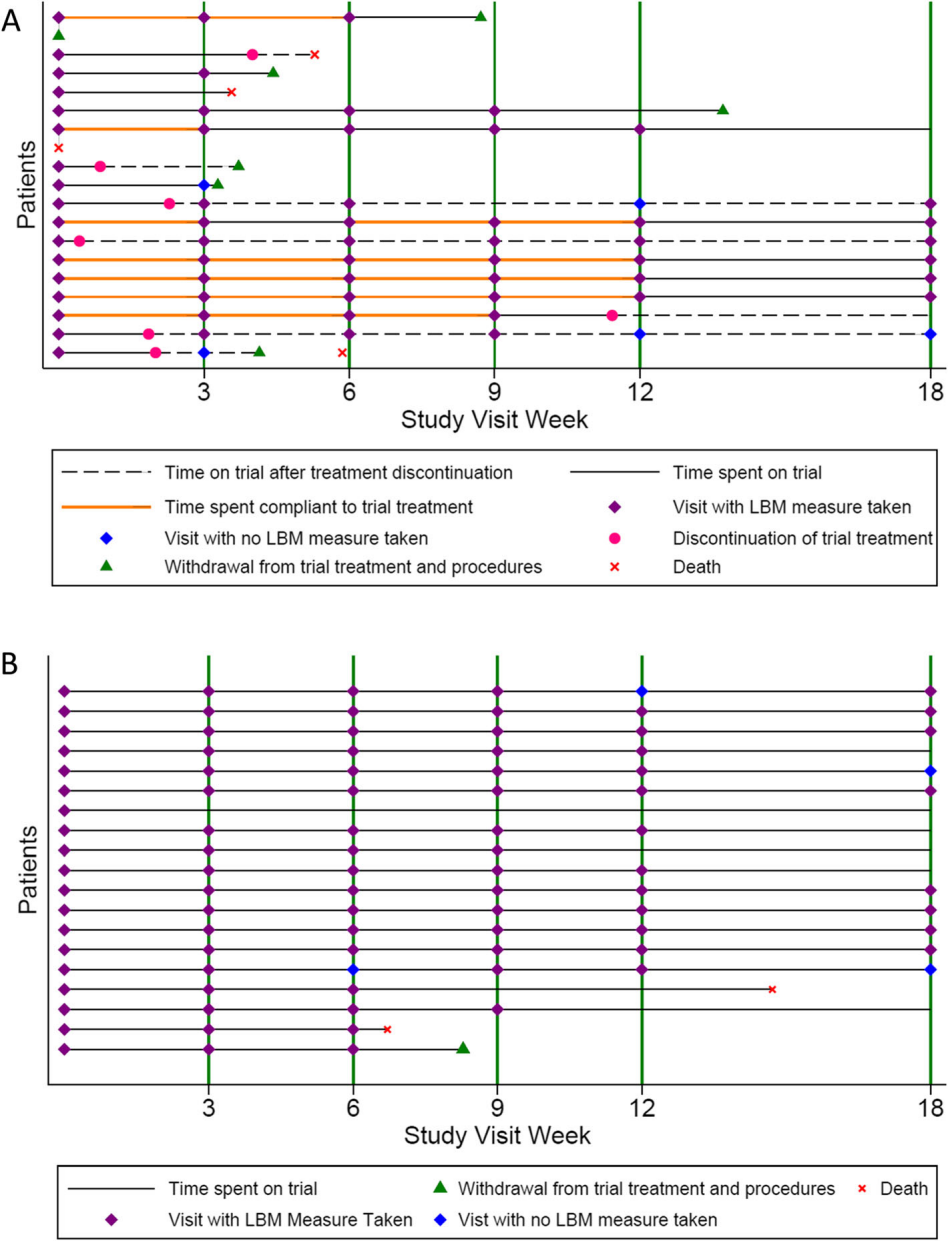
Adherence to treatment and scheduled study visits by randomised patients. Swimmer plots to visualise adherence to treatment and attendance at scheduled study visits of patients randomised to receive HMB/Arg/Gln (A) or patients randomised receive no HMB/Arg/Gln (B). The pathway of each individual patient is represented by a horizontal line and relevant outcome/symbols as per the key. HMB/Arg/Gln, β-Hydroxy β-Methylbutyrate/Arginine/Glutamine; LBM, lean body mass

Adherence to treatment and scheduled study visits on both treatment arms are summarised in Figs. 2 and 3. Of the 19 patients randomised to the HMB/Arg/Gln arm, 17 attended the baseline visit and were provided with treatment but only 7 patients were still taking experimental drug at the time of their next visit; 4 patients went on to take treatment for the full 12 weeks as planned (with one of these having a 3-week break), one took treatment for 9-weeks, one for 6-weeks and one for 3-weeks. The main reason stated for non-adherence of 10 patients between the baseline and week 3 visit was “Not Acceptable/Unpalatable” trial treatment (4; this included 2 patients who subsequently withdrew their consent from the trial). Further reasons included withdrawal of consent from trial (n = 1)and deterioration of the patients’ condition (n = 1), with the remainder unspecified. At subsequent visits, patient withdrawal (n = 5) and forgetting to take the trial treatment (n = 3) were noted as the main reasons for non-adherence. In terms of adherence to study visits in the experimental treatment arm, only 7 patients attended all their planned visits during the 12-week study period, with the remainder being 6 withdrawals, 3 deaths and 3 with missing visits.

Of the 19 patients randomised to the no HMB/Arg/Gln arm, one died and one withdrew during the 12-week study period, and 13 attended all their planned study visits during this time. Four patients missed visits.

There were no Serious Adverse Events reported in the trial.

Of the 38 patients randomised for the primary analysis, 10 were evaluable for primary outcome analysis from the HMB/Arg/Gln arm, and 13 from the control (Fig. 2). Three treatment successes were reported in the experimental arm compared to nine in the control arm (Table 2). For an intention-to-treat (ITT) analysis, patients with missing primary outcome data were combined with those who were recorded as failures and those who died. The main reasons for data not being available were withdrawal of patients within the HMB/Arg/Gln arm, and patients not attending the week 12 clinic visit in the no HMB/Arg/Gln arm (Table 2). The ITT analysis shows treatment success rate of 16% on the HMB/Arg/Gln arm and 47% on the control arm. The odds ratio comparing the success rate for experimental treatment versus control is estimated as 0.210 with 95% confidence interval 0.045 to 0.960. This indicates that the odds of treatment success are reduced with HMB/Arg/Gln, the opposite to that hypothesised.

**Table 2:** Primary outcome analysis

	HMB/Arg/Gln *n* = 19 N (%)	No HMB/Arg/Gln *n* = 19 N (%)
**Success (alive without a drop of >5% in LBM)**	3 (16)	9 (47)
**Failure (alive with a drop of >5% in LBM)**	3 (16)	3 (16)
**Died**	4 (21)	1 (5)
**Data not-available**	9 (47)	6 (32)
**Reasons for unavailable data**		
Patient withdrawal	6 (67)	1 (17)
None attendance at week 12 visit	2 (22)	4 (66)
Measure not taken	1 (11)	1 (17)

*HMB/Arg/Gln* β-Hydroxy β-Methylbutyrate/Arginine/Glutamine *LBM* Lean body mass

**Fig. 4:**
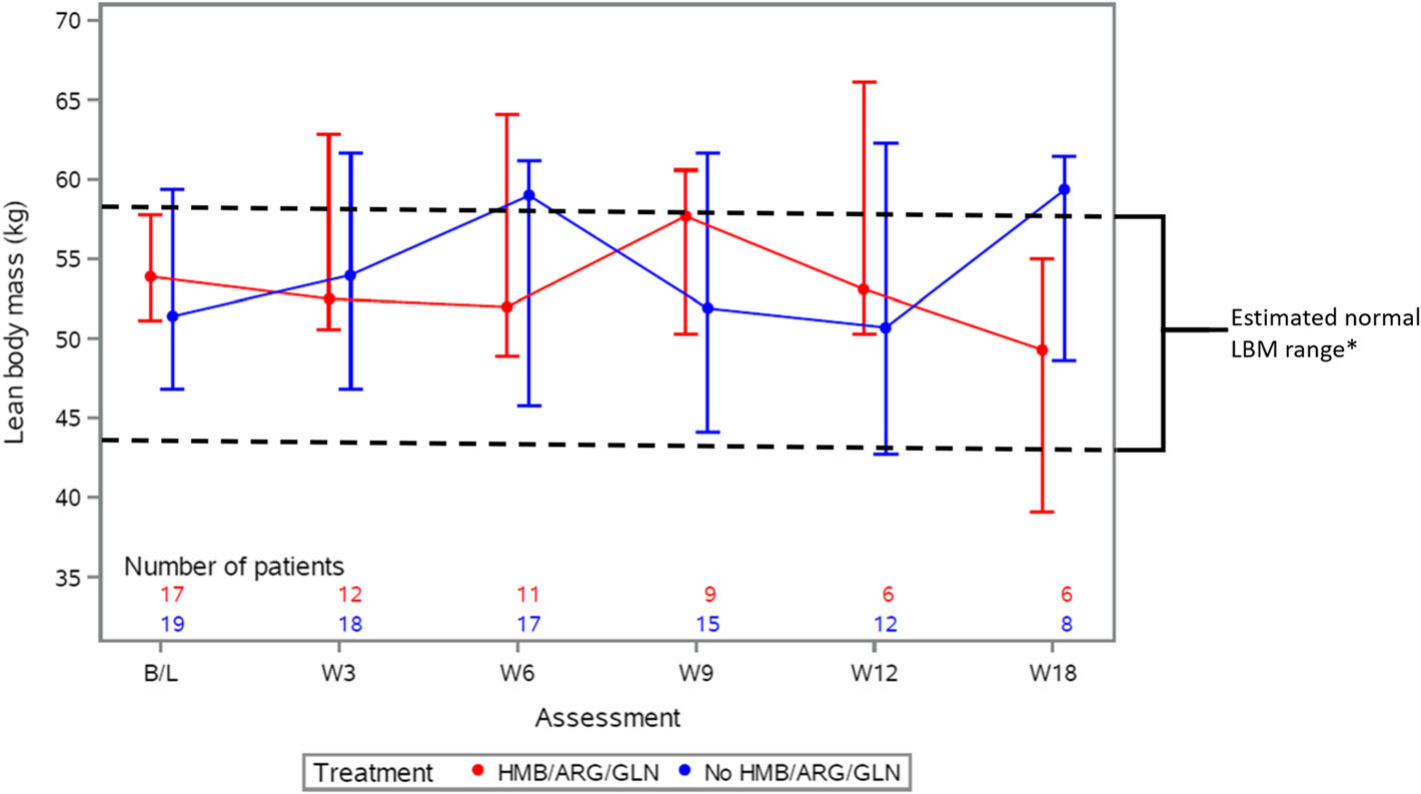
Change in lean body mass. Median and interquartile ranges of patients’ lean body mass comparing those randomised to receive HMB/Arg/Gln (red) or no HMB/Arg/Gln (blue). The number of patients included at each assessment is noted within the graph, under the relevant assessment, and colour-coded per arm. Only those patients with available measurements were included. * Estimates of normal LBM range assumes the following: Ideal body weight of a 5′10″ male = 73 kg, with approximate LBM of 80%, and; ideal body weight of 5′5″ female = 61.5 kg, with approximate LBM of 70%. B/L, baseline; W, week; HMB/Arg/Gln, β-Hydroxy β-Methylbutyrate/Arginine/Glutamine

**Fig. 5:**
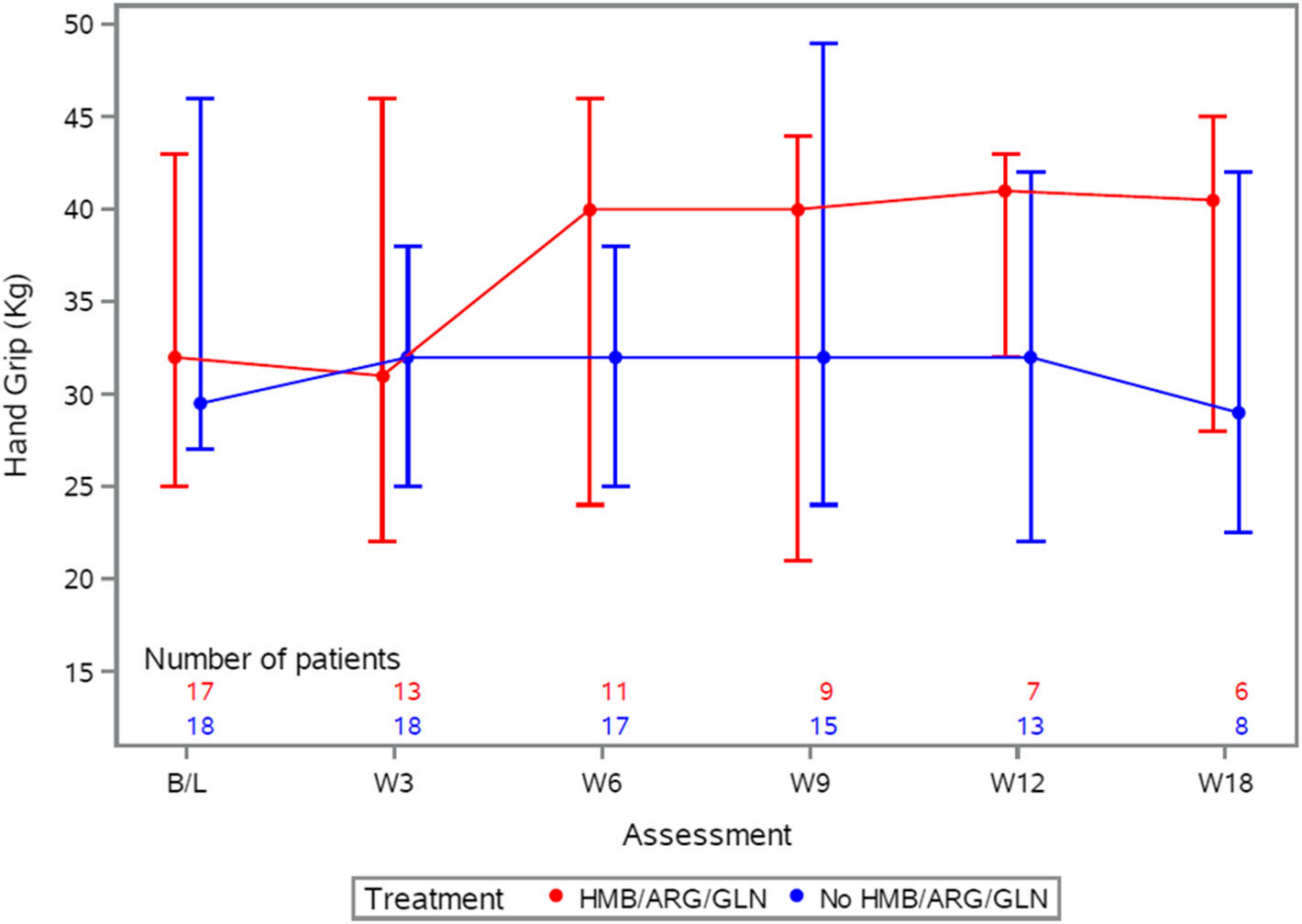
Change in handgrip strength. Median and interquartile ranges of patients’ dynamometer isometric grip force comparing those randomised to receive HMB/Arg/Gln (red) or no HMB/Arg/Gln (blue). The number of patients included at each assessment is noted within the graph, under the relevant assessment, and colour-coded per arm. Only those patients with available measurements were included. HMB/Arg/Gln, β-Hydroxy β-Methylbutyrate/Arginine/Glutamine

Analysis of secondary outcomes showed no evidence of a difference between arms in terms of change in LBM and handgrip strength over the 12 weeks post randomisation and no clear trend over time in either measure (Figs. 4 and 5). In terms of the FAACT QoL score, the mean change at 12 weeks from baseline was a decrease of − 12 i.e., worsening, on the HMB/Arg/Gln arm compared to an increase of + 6 i.e. improvement, on the control arm.

## Discussion

The NOURISH trial was a randomised phase II trial designed as a pilot to detect a signal that dietary supplementation with HMB/Arg/Gln, on a background of nutritional and symptom support, delays the onset of cachexia in patients with advanced lung cancer sufficiently to justify further investigation in a randomised phase III trial. It was the intention that a phase III trial would formally test the hypothesis that the intervention results in clinical benefit.

The interim and final analyses demonstrated poor treatment adherence. In addition, the analysis provided no evidence to show that the intervention delayed the onset of cachexia in this patient population. The conclusion from this trial therefore, was further evaluation of HMB/Arg/Gln in this setting could not be recommended without strategies to address the slow recruitment and tolerability of the intervention.

Although recruitment issues were anticipated, as observed in other interventional trials for cancer cachexia reported prior to initiation of this trial [24, 25], the trial remained open for 2 years in the anticipation that recruitment would improve in this most clinically relevant population of patients receiving palliative treatment. This issue was addressed in a randomised controlled trial of different service delivery models to improve pain control in the palliative setting, published after the NOURISH trial was stopped [31]. Furthermore, even though recruitment during the NOURISH trial was focused at the earliest point in the clinical pathway, rather than only after symptoms of cachexia were noted, this trial concluded these issues still persisted suggesting most if not all patients who took part were not pre-cachectic. It is interesting to note that, given the high proportion of NOURISH trial patients with NSCLC with metastatic disease, a very recent study suggests the majority of patients with advanced NSCLC present with some degree of cachexia [32].

A contributory factor to the high dropout rate may have been intolerance of the product (powder dissolved in cold water or juice); feedback from the patient diaries suggested that many patients found the trial intervention unpalatable. Although the supplement was reported to be well tolerated in healthy subjects [23], it may be less tolerated in this patient cohort. Whether this is due to disease-related symptoms and toxicities associated with other treatments such as chemotherapy which rendered patients less able to tolerate the trial intervention is unknown.

Of note, initial concepts for, and development of this trial included placebo control to minimise bias. Ethical approval was obtained for this study design and site initiation arranged on this basis. During set-up of this trial, although the trial intervention and a matched placebo were sourced, the offer to fund this element of the trial was withdrawn in 2011 due to the changes in research priorities of the company during an economically uncertain period. As a result, the trial was redesigned as an open-label study and a substantial amendment approved by the ethics committee. This change in trial design had a negative effect on the planned opening of one site which withdrew their participation due to the lack of placebo control. This also negatively impacted on trial recruitment.

Since closure of the NOURISH trial, two Japanese trials have assessed the clinical benefit of HMB/Arg/Gln within supportive care measures in patients undergoing chemoradiotherapy due to head and neck cancers [33], and perioperatively in patients scheduled to undergo open surgery for abdominal malignancies [34]. Adherence rates in both trials were high, with conclusions generally in favour of further investigation in larger phase III trials to reduce the incidence of chemoradiotherapy-induced oral mucositis and post-operative wound complications, respectively. Its use in patients with cachexia and or advanced lung cancer however has not been repeated in any subsequent trials.

Since the time this trial was conceived and developed, there has been an important shift in the approach to research in cancer cachexia resulting in the formation of an international consensus group to address the lack of consistency in cancer cachexia definition, diagnosis and trial design [4]. In addition, a recent review has urged medical regimens to not only treat the cancer site but to provide a personalised nursing-based intervention specific to cachexia in the hope of inhibiting progression of this debilitating syndrome and improve patient QoL [8]. To this end the use of the MDCP was successfully incorporated by nurses and dieticians across the five hospitals involved in this trial. Anecdotal evidence from trial patients reported benefit of the nutritional supplements (including HMB/Arg/Gln), pharmacological interventions and exercises that were prescribed. An unexpected but important success of the trial was the raised awareness of the MDCP in participating centres, which we hope the principles it contains will continue to be employed beyond the context of the trial. The Mulitmodal-Exercise, Nutrition and Anti-inflammatory medication for Cachexia (MENAC) trial which is currently open to recruitment aims to address these issues through a multi-modality approach to cancer cachexia [35].

## Conclusions

The NOURISH trial sought to assess whether the nutritional supplement HMB/Arg/Gln given on a background of structured nutritional and symptom support, could delay the onset of cachexia in patients with advanced lung cancer sufficiently to justify further investigation in a larger phase III trial. The key novelty of NOURISH was that patients did not require weight loss to be eligible for the trial. In addition, the incorporation of the MDCP for all eligible patients, and its specificity for patients with advanced lung cancer, set it apart from previously published trials at the time. Early closure of the trial due to slow recruitment and partial adherence, suggests that further investigation within this setting may not be appropriate unless the issue of palatability is addressed. In addition, any future trials will need to be designed with improved strategies for recruitment. Despite these issues, however, the NOURISH trial demonstrated that the use of the MDCP for cancer cachexia by healthcare professionals may be considered as a useful tool in the care of these patients. The benefit of this type of approach is supported by recent trials testing the use of new psychoeducational interventions in patients with cancer cachexia [36, 37].

## Supplementary Information

**Additional file 1: Supplementary Appendix 1.** Macmillan Durham Cachexia Pack. The Macmillan Durham Cachexia Pack (MDCP) was a resource developed in 2007 by Macmillan and a multi-professional team in County Durham and Darlington NHS Foundation Trust with support from professionals around the UK. It provides an evidence-based guide for healthcare professionals to assess and manage common symptoms and problems seen in patients with anorexia-cachexia syndrome. The pack also contains a number of leaflets to help patients and their families deal with the emotional and psychological impact of the condition. As a non-copyrighted resource, freely provided and distributed on CD-ROM to enable materials to be printed out according to need, it was originally available online [26] but has since been superseded. Permission for the use and inclusion of the MDCP within this manuscript was gained in writing from the Pack Editor and Clinical Lead, Dr. Colette Hawkins.

## Data Availability

Participant data and the associated supporting documentation will be available within six months after the publication of this manuscript. Details of our data request process, including the Data Sharing Request Form, is available on the CRCTU website (https://www.birmingham.ac.uk/research/crctu/data-sharing-policy.aspx). The completed form should be sent to NewBusines@trial.bham.ac.uk. Only scientifically sound proposals from appropriately qualified research groups will be considered for data sharing. The decision to release data will be made by the CRCTU Director’s Committee, who will consider the scientific validity of the request, the qualifications and resources of the research group, the views of the Chief Investigator and the trial steering committee, consent arrangements, the practicality of anonymising the requested data and contractual obligations. A data sharing agreement will cover the terms and conditions of the release of trial data and will include publication requirements, authorship and acknowledgements and obligations for the responsible use of data. An anonymised encrypted dataset will be transferred directly using a secure method and in accordance with the University of Birmingham’s IT guidance on encryption of data sets.

## Abbreviations

BIA: Bioelectrical impedance analysis
CRCTU: Cancer Research UK Clinical Trials Unit
DMC: Data Monitoring Committee
FAACT: Functional Assessment of Anorexia Cachexia Therapy
FFM: Fat-free mass
HMB: β-hydroxy β-methyl butyrate
HMB/Arg/Gln: β-hydroxy β-methyl butyrate in combination with arginine and glutamine
IMP: Investigational medicinal product
LBM: Lean body mass
LMF: Lipolysis and lipid-mobilising factor
MDCP: Macmillan Durham Cachexia Pack
MENAC: Mulitmodal-Exercise, Nutrition and Anti-inflammatory medication for Cachexia
NSAIDs: Non-steroidal anti-inflammatory drugs
NSCLC: Non-small cell lung cancer
TNFα: Tumour necrosis factor-α
PG-SGA: Patient Generated Subjective Global Assessment
PIF: Proteolysis-inducing factor
QoL: Quality of life
SCLC: Small cell lung cancer

## References

[1] von Haehling S, Anker SD (2010). Cachexia as a major underestimated and unmet medical need: facts and numbers. J Cachexia Sarcopenia Muscle.

[2] Sørensen J (2018). Lung cancer cachexia: can molecular understanding guide clinical management?. Integr Cancer Ther.

[3] Hug A, Phillips I, Allan L, Ezhil V (2016). Rate of cachexia in lung cancer. Eur J Surg Oncol (EJSO).

[4] Fearon K, Strasser F, Anker SD, Bosaeus I, Bruera E, Fainsinger RL, Jatoi A, Loprinzi C, MacDonald N, Mantovani G, Davis M, Muscaritoli M, Ottery F, Radbruch L, Ravasco P, Walsh D, Wilcock A, Kaasa S, Baracos VE (2011). Definition and classification of cancer cachexia: an international consensus. Lancet Oncol.

[5] Mytelka DS, Li L, Benoit K (2018). Post-diagnosis weight loss as a prognostic factor in non-small cell lung cancer. J Cachexia Sarcopenia Muscle.

[6] Arrieta O, Michel Ortega RM, Villanueva-Rodríguez G, Serna-Thomé MG, Flores-Estrada D, Diaz-Romero C (2010). Association of nutritional status and serum albumin levels with development of toxicity in patients with advanced non-small cell lung cancer treated with paclitaxel-cisplatin chemotherapy: a prospective study. BMC Cancer.

[7] Andreyev HJN, Norman AR, Oates J, Cunningham D (1998). Why do patients with weight loss have a worse outcome when undergoing chemotherapy for gastrointestinal malignancies?. Eur J Cancer.

[8] Zhu R, Liu Z, Jiao R, Zhang C, Yu Q, Han S, Duan Z (2019). Updates on the pathogenesis of advanced lung cancer-induced cachexia. Thorac Cancer.

[9] Bruggeman AR, Kamal AH, LeBlanc TW, Ma JD, Baracos VE, Roeland EJ (2016). Cancer Cachexia: beyond weight loss. J Oncol Pract.

[10] Ruiz Garcia V, López-Briz E, Carbonell Sanchis R, Gonzalvez Perales JL, Bort-Marti S (2013). Megestrol acetate for treatment of anorexia-cachexia syndrome. Cochrane Database Syst Rev.

[11] Strasser F, Luftner D, Possinger K, Ernst G, Ruhstaller T, Meissner W (2006). Comparison of orally administered cannabis extract and delta-9-tetrahydrocannabinol in treating patients with cancer-related anorexia-cachexia syndrome: a multicenter, phase III, randomized, double-blind, placebo-controlled clinical trial from the cannabis-in-cachexia-study-group. J Clin Oncol.

[12] Paulsen Ø, Klepstad P, Rosland JH, Aass N, Albert E, Fayers P, Kaasa S (2014). Efficacy of methylprednisolone on pain, fatigue, and appetite loss in patients with advanced cancer using opioids: a randomized, placebo-controlled, double-blind trial. J Clin Oncol.

[13] Solheim TS, Fearon KCH, Blum D, Kaasa S (2013). Non-steroidal anti-inflammatory treatment in cancer cachexia: a systematic literature review. Acta Oncol.

[14] Gordon JN, Trebble TM, Ellis RD, Duncan HD, Johns T, Goggin PM (2005). Thalidomide in the treatment of cancer cachexia: a randomised placebo controlled trial. Gut..

[15] Temel JS, Abernethy AP, Currow DC, Friend J, Duus EM, Yan Y, Fearon KC (2016). Anamorelin in patients with non-small-cell lung cancer and cachexia (ROMANA 1 and ROMANA 2): results from two randomised, double-blind, phase 3 trials. Lancet Oncol.

[16] Currow D, Temel JS, Abernethy A, Milanowski J, Friend J, Fearon KC (2017). ROMANA 3: a phase 3 safety extension study of anamorelin in advanced non-small-cell lung cancer (NSCLC) patients with cachexia. Ann Oncol.

[17] Katakami N, Uchino J, Yokoyama T, Naito T, Kondo M, Yamada K, Kitajima H, Yoshimori K, Sato K, Saito H, Aoe K, Tsuji T, Takiguchi Y, Takayama K, Komura N, Takiguchi T, Eguchi K (2018). Anamorelin (ONO-7643) for the treatment of patients with non-small cell lung cancer and cachexia: results from a randomized, double-blind, placebo-controlled, multicenter study of Japanese patients (ONO-7643-04). Cancer..

[18] Williams JZ, Abumrad N, Barbul A (2002). Effect of a specialized amino acid mixture on human collagen deposition. Ann Surg.

[19] Zanchi NE, Gerlinger-Romero F, Guimarães-Ferreira L, de Siqueira Filho MA, Felitti V, Lira FS, Seelaender M, Lancha AH (2011). HMB supplementation: clinical and athletic performance-related effects and mechanisms of action. Amino Acids.

[20] Potenza MA, Nacci C, Mitolo-Chieppa D (2001). Immunoregulatory effects of L-arginine and therapeutical implications. Curr Drug Targets Immune Endocr Metabol Disord.

[21] Xi P, Jiang Z, Zheng C, Lin Y, Wu G (2011). Regulation of protein metabolism by glutamine: implications for nutrition and health. Front Biosci (Landmark Ed).

[22] Eley HL, Russell ST, Tisdale MJ (2008). Attenuation of depression of muscle protein synthesis induced by lipopolysaccharide, tumor necrosis factor, and angiotensin II by beta-hydroxy-beta-methylbutyrate. Am J Physiol Endocrinol Metab.

[23] Deutz NEP, Pereira SL, Hays NP, Oliver JS, Edens NK, Evans CM, Wolfe RR (2013). Effect of β-hydroxy-β-methylbutyrate (HMB) on lean body mass during 10 days of bed rest in older adults. Clin Nutr.

[24] Berk L, James J, Schwartz A, Hug E, Mahadevan A, Samuels M, Kachnic L, RTOG (2008). A randomized, double-blind, placebo-controlled trial of a β-hydroxyl β-methyl butyrate, glutamine, and arginine mixture for the treatment of cancer cachexia (RTOG 0122). Support Care Cancer.

[25] May PE, Barber A, D’Olimpio JT, Hourihane A, Abumrad NN (2002). Reversal of cancer-related wasting using oral supplementation with a combination of β-hydroxy-β-methylbutyrate, arginine, and glutamine. Am J Surg.

[26] Macmillan Cancer Support. 2007. Macmillan Durham Cachexia Pack. https://learnzone.org.uk/courses/course.php?id=67 Last accessed 10th May 2015.

[27] Hawkins C, Andrew I (2012). Raising awareness of the Macmillan Durham Cachexia pack (MDCP) within a local cancer network. BMJ Support Palliat Care.

[28] The Scored Patient-Generated Subjective Global Assessment. http://pt-global.org/?page_id=13 Last accessed 16th March 2021.

[29] Ribaudo JM, Cella D, Hahn EA, Lloyd SR, Tchekmedyian NS, Roenn JV, Leslie WT (2000). Re-validation and shortening of the functional Assessmentof anorexia/Cachexia therapy (FAACT) questionnaire. Qual Life Res.

[30] Jung S-H (2008). Randomized phase II trials with a prospective control. Stat Med.

[31] LeBlanc TW, Lodato JE, Currow DC, Abernethy AP (2013). Overcoming recruitment challenges in palliative care clinical trials. J Oncol Pract.

[32] White R, Weekes CE, Grant R, Baldwin C, Ahmed H. Determining the prevalence and severity of cancer cachexia in advanced non-small cell lung cancer and its relationship with chemotherapy outcomes. Support Care Cancer. 2020. 10.1007/s00520-019-5259-1. 10.1007/s00520-019-05259-1 PMC7378112 31916005

[33] Yokota T, Hamauchi S, Yoshida Y, Yurikusa T, Suzuki M, Yamashita A, Ogawa H, Onoe T, Mori K, Onitsuka T (2018). A phase II study of HMB/Arg/Gln against oral mucositis induced by chemoradiotherapy for patients with head and neck cancer. Support Care Cancer.

[34] Wada N, Kurokawa Y, Tanaka K, Miyazaki Y, Makino T, Takahashi T, Wada H, Yamasaki M, Yamasaki M, Nakajima K, Eguchi H, Takiguchi S, Mori M, Doki Y (2018). Perioperative nutritional support with beta-hydroxy-beta-methylbutyrate, arginine, and glutamine in surgery for abdominal malignancies. Wounds..

[35] Solheim TS, Laird BJA, Balstad TR, Bye A, Stene G, Baracos V, Strasser F, Griffiths G, Maddocks M, Fallon M, Kaasa S, Fearon K (2018). Cancer cachexia: rationale for the MENAC (multimodal-exercise, nutrition and anti-inflammatory medication for Cachexia) trial. BMJ Support Palliat Care.

[36] Buonaccorso L, Bertocchi E, Autelitano C, Allisen Accogli M, Denti M, Fugazzaro S, Martucci G, Costi S, Tanzi S (2021). Psychoeducational and rehabilitative intervention to manage cancer cachexia (PRICC) for patients and their caregivers: protocol for a single-arm feasibility trial. BMJ Open.

[37] Molassiotis A, Brown T, Cheng HL, Byrnes A, Chan RJ, Wyld D, Eastgate M, Yates P, Marshall AP, Fichera R, Isenring L, To KF, Ko PS, Lam W, Lam YF, Au LF, Lo RSK (2021). The effects of a family-centered psychosocial-based nutrition intervention in patients with advanced cancer: the PiCNIC2 pilot randomised controlled trial. Nutr J.

