# Supplementary material for: Beta-hydroxy beta-methylbutyrate/arginine/glutamine (HMB/Arg/Gln) supplementation to improve the management of cachexia in patients with advanced lung cancer: an open-label, multicentre, randomised, controlled phase II trial (NOURISH)
Source: BMC Cancer. 2021 Jul 12;21:800. doi: 10.1186/s12885-021-08519-8 (PMC8274132; doi:10.1186/s12885-021-08519-8)
Supplement: Supplementary file 1 — Additional file 1: Supplementary Appendix 1. Macmillan Durham Cachexia Pack. The Macmillan Durham Cachexia Pack (MDCP) was a resource developed in 2007 by Macmillan and a multi-professional team in County Durham and Darlington NHS Foundation Trust with support from professionals around the UK. It provides an evidence-based guide for healthcare professionals to assess and manage common symptoms and problems seen in patients with anorexia-cachexia syndrome. The pack also contains a number of leaflets to help patients and their families deal with the emotional and psychological impact of the condition. As a non-copyrighted resource, freely provided and distributed on CD-ROM to enable materials to be printed out according to need, it was originally available online [26] but has since been superseded. Permission for the use and inclusion of the MDCP within this manuscript was gained in writing from the Pack Editor and Clinical Lead, Dr. Colette Hawkins. [file 12885_2021_8519_MOESM1_ESM.pdf]

Background

What is cachexia?

Cachexia is the involuntary weight loss associated with certain diseases, of which malignancy is one. Preferential loss of muscle and visceral protein is typical and the speed and extent to which this occurs can be dramatic. The underlying pathological pathways driving cachexia are complex and only partially understood. In practice, cachexia is usually seen within the anorexia-cachexia syndrome (ACS). This can cause:

Physical consequences

- anorexia (reduced appetite / interest in food)
- weight loss (including loss of muscle)
- a range of symptoms, which may include:
  - early satiety (feeling full quickly)
  - nausea (with or without vomiting)
  - mouth problems
  - bowel changes (constipation or diarrhoea)
  - fatigue and weakness.

Psychosocial and emotional consequences

- for both patients and their carers may include:
  - anxiety
  - conflict around food
  - altered body image as a result of weight loss
  - low mood.

What is this pack?

- The Macmillan Durham Cachexia Pack is a resource for professionals, to guide them in the assessment and management of common problems seen in patients with ACS.
- It has been developed by a multiprofessional team and pulls together resources relevant to the management of ACS.
- It is intended to facilitate timely screening, assessment, intervention and / or referral to relevant health professionals in order to manage the physical, psychological and emotional impact of ACS more effectively.
- The resources have been developed using the evidence base, where possible, or current best practice in the absence of an evidence base.
- The pack is a guide and is not intended to be prescriptive. Certain parts can be adapted according to local policy and developing evidence.
- The pack concentrates on managing the consequences of ACS rather than treatments intended to influence the underlying pathophysiology.

How to use the pack

This pack is designed to give you a framework to be able to implement a system of managing cachexia in your organisation.

Which patients is the pack meant for?

- all patients with advanced cancer
- all patients with lung cancer, regardless of stage
- all patients with upper gastrointestinal cancer (oesophago-gastric or hepato-biliary), regardless of stage
- basic principles could be used in patients with cachexia from non-malignant conditions such as heart failure, but the evidence base for this pack, together with the experience with its use, is predominantly from work and research in patients with cancer.

How should I use the pack?

- Use at any stage of the patient's illness:
    - as a screening tool
    - as a tool for standardised assessment and management.
- **Always start with the assessment tool** (*Section 1*)
    - any healthcare professional can use the assessment tool with patients at any stage of their illness
    - where possible, allow the patient to complete the tool themselves with support where needed
    - the answers in each of the four quadrants in the assessment tool will direct you to relevant sections of the pack.
- The assessment tool can be used repeatedly throughout a patient's illness.
- Remember to offer the patient information leaflets.
- There is a patient information leaflet about the pack ('The Macmillan Durham Cachexia Pack: Problems with Eating and Weight Loss in Cancer', *Section 5*). This gives patients and carers an overview of the anorexia-cachexia syndrome and the leaflets available to them from the pack. This leaflet can be displayed locally (for example, cancer information centres and clinical areas).

CD-ROM

All documents within the pack are available on the enclosed CD-ROM for local adaptation and reprinting. The CD-ROM enables you to add your organisation's logo onto the documents and enables you to print out appropriate numbers of the documents, depending on the needs in your area.

The pack tries to give as much flexibility as possible to allow you to use the pack within your current documentation and systems of care delivery. Some areas of the documents, however, are locked where alteration would significantly alter the use of the tool.

How to use the CD: Place the CD in the tray and load the disk. It should auto run. If it does not auto run, go to your CD drive, right click the icon and select open, then double click on the file Cachexiapack.pdf. This will launch the application. Minimum system requirements are a PC with minimum 64MB RAM running Windows 98 with Microsoft Word 97 and Adobe Acrobat 4.

Once open, click on 'Sections'. Each document can be accessed here, by clicking on the title. Close the document by clicking the lower small grey crossed box, in the top right hand corner.

What is in the pack?

Section 1: Assessment tool

- **Abridged Patient-Generated Subjective Global Assessment (PG-SGA)**
  - The tool provides an overview of four key areas of the patient's clinical condition: change in weight over time, food intake, symptom profile and activities and function
  - The tool directs to relevant sections of the pack for management options, appropriate advice and related patient information leaflets.

Section 2: Dietary strategies

- Two algorithms to identify basic dietary interventions that can be instituted immediately and to identify patients who would benefit from early assessment by a dietitian. Use one algorithm depending on the condition of the patient:
  - **Dietary Algorithm 1**: for patients in the early palliative stages of illness
  - **Dietary Algorithm 2**: for patients in the late palliative stages of illness

- **Guidelines for using nutritional supplements** to help you identify when and what to prescribe according to the patient's needs

- **Focus on Food patient / carer information leaflets** to highlight strategies for eating well and food fortification

- **Patient fact sheets on storage and use of nutritional supplements and recipes** to give to patients and / or their carers to help make the most of the supplements prescribed.

Section 3: Exercise strategies

This section contains two algorithms with associated programmes of exercise / activity which will help guide you to give appropriate advice to patients / carers.

- **Exercise Algorithm 1**: an algorithm for healthcare professionals to initiate exercises for patients with mild / moderate functional limitation as identified on PG-SGA

- **Patient Exercise Programme 1**: an exercise sheet for patients as defined in Exercise Algorithm 1

- **Exercise Algorithm 2**: an algorithm for healthcare professionals to initiate exercises for patients with more severe functional limitation as identified on PG-SGA

- **Patient Exercise Programme 2**: an exercise sheet for patients as defined in Exercise Algorithm 2

- **Pacing and Daily Activities**: an information sheet for patients with fatigue.

Section 4: Management strategies

This section provides advice on how best to manage the common symptoms encountered in anorexia-cachexia syndrome.

- **Management strategies table:**
  - Each management strategy covers both non-pharmacological and pharmacological options
  - Each management strategy for a symptom, guides you to the leaflets can be used from Section 5 to support your treatment

- **Mouth care**: a patient / carer fact sheet to encourage good oral hygiene.

Section 5: General patient information leaflets

This section contains patient information leaflets that can be printed out and used by the healthcare professional and given to the patient and / or their carers dependant on the needs identified in the assessments.

Section 6: Evidence base

This section contains a review of the evidence base that has informed the development of this pack as well as the tools within the pack. This evidence is current as of September 2007.

- **Dietary strategies**

- **Exercise strategies**

- **Symptom management strategies**.

Acknowledgements

Durham Cachexia Project Team:

Pack Editor and Clinical Lead*:* Colette Hawkins, Consultant in Palliative Medicine, University Hospital of North Durham

Project Steering Group Chairman*:* Graeme Kirkpatrick, Deputy Chief Pharmacist, County Durham & Darlington Foundation Trust

Project Researchers:
Inga Andrew, Macmillan Senior Clinical Pharmacist, University Hospital of North Durham
Kerry Waterfield, Staff Grade in Palliative Medicine, University Hospital of North Durham

Macmillan Development Manager: Stephen Williams, Macmillan Cancer Support

Additional Contributors:

- Tessa Aston, Macmillan Dietitian / AHP Lead North of England Cancer Network
- Christine Baldwin, Lecturer in Nutrition and Dietetics, Royal Marsden Hospital, London
- Trevelyan Beyer, Specialist Physiotherapist, St. Cuthbert's Hospice, Durham
- Yvonne Bottle, 'Focus on Food' Project Dietitian (North Durham), County Durham and Darlington NHS Foundation Trust
- Jane Hopkinson, Senior Research Fellow, Macmillan Research Unit, University of Southampton
- Rachael Masters, Highly Specialist Dietitian (South Durham), County Durham and Darlington NHS Foundation Trust
- Carolyn Wyatt, Macmillan Information Centre Manager, University Hospital of North Durham.

With thanks to:

- Macmillan Cancer Support
- Sam Johnston and the North Durham Palliative Care Team
- Staff and patients of University Hospital of North Durham
- Yan Yiannakou, Consultant Gastroenterologist, University Hospital of North Durham
- Christine Wyn-Jones, Head of Nutrition and Dietetics, and Fiona Elliott, Senior Dietitian, University Hospital of North Durham
- Joan James, Lead Cancer Nurse, University Hospital of North Durham
- Ann Fox and the North of England Cancer Network
- Macmillan Research Unit, University of Southampton
- The 'Focus on Food' initiative; Nutrition and Dietetic Services, South Durham, Darlington and North Durham
- Cancer Backup
- Succinct Healthcare Communications and Consultancy.

Abridged Patient-Generated Subjective Global Assessment (PG-SGA)

Please complete the following

Patient ID

1. Weight	2. Food Intake
In summary of my current and recent weight:<br><br>I currently weigh about ______________<br>I am about ____________ tall<br>One month ago I weighed about __________<br>Six months ago I weighed about __________<br>During the past two weeks my weight has:<br>☐ Decreased ☐ Not changed ☐ Increased	As compared with my normal intake, I would rate my food intake during the past month as:<br>☐ Unchanged ☐ More than usual ☐ Less than usual<br><br>I am now taking:<br>☐ Normal food, but less than normal amount<br>☐ Little solid food<br>☐ Only liquids<br>☐ Only nutritional supplements<br>☐ Very little of anything<br>(the five boxes above → *See Sections 2 and 5*)<br>☐ Only tube feedings or nutrition by vein

3. Symptoms	4. Activities and Function
I have had the following problems that have kept me from eating enough during the past two weeks (tick all that apply):<br>☐ No problem eating<br>☐ No appetite, did not feel like eating<br>☐ Constipation<br>☐ Mouth sores<br>☐ Food tasting funny / having no taste<br>☐ Pain: where? ________________<br>☐ Other* ____________________<br>☐ Vomiting<br>☐ Nausea<br>☐ Diarrhoea<br>☐ Dry mouth<br>☐ Smells bother me<br>☐ Problems swallowing<br>☐ Feeling full quickly<br><br>*Examples: fatigue (see *Section 3* 'Pacing and Daily Activities'), depression, financial concerns *(see Section 5)* or dental problems<br><br>See Management Algorithm *(Section 4)*	Over the past month, I would generally rate my activity as (please tick only one box):<br>☐ Normal with no limitations *(no action required)*<br>☐ Not my normal, but able to be up and about with fairly normal activities<br>☐ Not feeling up to most things, but in bed or chair for less than half of the day<br>(the two boxes above → *See Section 3 Algorithm / Programme 1*)<br>☐ Able to do little activity and spend most of the day in bed or chair<br>☐ Pretty much bedridden, rarely out of bed<br>(the two boxes above → *See Section 3 Algorithm / Programme 2*)<br><br>See also 'Pacing and Daily Activities' *(Section 3)*

Dietary Strategies

This section contains two algorithms to help you to identify the most appropriate dietary strategies for your patients. Use after general assessment with the PG-SGA tool *(Section 1)*.

Dietary Algorithm 1: for patients at early palliative care stages

Early palliative care is defined as:

- patient diagnosed with a disease that cannot be cured; death is not likely to be imminent and the patient may have months or even years of life left and quality of life may be good
- patient may be undergoing palliative treatment to help to improve quality of life.

Dietary Algorithm 2: for patients at late palliative care stages

Late palliative care is defined as:

- patient experiencing a general deterioration in condition. Appetite reduces and the patient becomes increasingly fatigued. Other symptoms may also be exacerbated. Carers' anxieties may worsen at this time and they may become increasingly concerned about the patient's food intake.

The goal of nutrition therapy should NOT be weight gain or reversal of malnutrition, but it should be about quality of life, including comfort, symptom relief and enjoyment of food. Aggressive feeding may not be appropriate, especially if eating and drinking cause discomfort and / or anxiety to the patient. Therefore weighing patients is not advised.

Anxiety around nutrition is very common, particularly amongst carers. You need to be aware of the potential tensions that may arise between people living with cancer and their carers with respect to the patient's loss of appetite. It is important not to overlook this and to offer opportunity for concerns to be expressed. You may find some of the patient information leaflets *(Section 5)* helpful in addressing patient and / or carer concerns.

Dietary Algorithm 1

Weight loss and / or reduced appetite
(Use **Dietary Algorithm 2** for patients at late palliative care stages)

↓

Investigate contributing factors:

- Check symptoms / medication *(see Section 4)*
- Check practical points
  - Refer to a dietitian at ANY stage if you have concerns
  - Support needed for eating / drinking
  - Cooking and food availability
  - Appearance of food / drink
  - Confusion / forgetfulness
  - Dentures / oral hygiene
  - Environment
  - Finances
  - Posture
  - Special diet

↓

Patient agrees to dietary intervention

↓

Advise on:

- Nourishing snacks and drinks
- Eating little and often
- Food fortification

See Section 2 (Focus on food leaflets) and Section 5

Review within one month, or sooner if clinical concern

Patient anxious and / or unable to manage food fortification →

Issue:

- ONE WEEK prescription of Complan® Shake at TWO sachets per day

OR Other fresh milk-based supplement at ONE sachet per day

OR Starter Pack at TWO cartons per day

See Section 2 (Guidelines for using nutritional supplements), offer patient fact sheets (Section 2)

↓

Issue ONE MONTH prescription of supplement

Review every month

All patients should be reviewed by a dietitian after six months of supplement prescription

Appetite improved / weight stable →

Continue nutrition support

Reassess within one month, or sooner if clinical concern

Supplement not tolerated or patient anxious about nutrition →

Refer to dietitian

If patient does not agree to dietary intervention, explain the potential benefits for symptom control. Patient may accept some dietary advice. Reassess within one month, or sooner if clinical concern.

See Sections 2 (Focus on food leaflets), 4 and 5

Dietary Algorithm 2

NB. This algorithm is intended for use with patients who are too unwell to manage meals, rather than for patients who have difficulties swallowing

Be clear of your goals:

- Reassurance and support to patient and carers—anorexia and weight loss are normal in advanced disease *(See Section 5 for leaflets)*
- Enjoyment of nourishing food and drinks where possible (so long as this does not increase patient distress)
- Treat reversible symptoms—eg constipation *(See Section 4)*

Weight gain is an unrealistic goal

↓

Consider practical difficulties:

- Refer to a dietitian at ANY stage if you have concerns
- Support needed for eating / drinking
- Cooking and food availability
- Appearance of food / drink
- Confusion / forgetfulness
- Dentures / oral hygiene
- Environment
- Finances
- Posture
- Special diet

↓

Identify food and / or drinks that the patient enjoys and encourage their consumption

↓

Consider recipes for nourishing drinks *(See Section 2, Focus on food leaflets)*

↓

Nutritional supplements

Before using any nutritional supplements consider the following:

- May only be of benefit to patients on psychological grounds
- Avoid making patient feel they have to take these
- Avoid giving false hope that they will improve nutritional status
- Discuss with GP / multidisciplinary team if you need further advice

If you decide to use nutritional supplements, try 'over-the-counter' products—eg Build-up®, Complan® Foods—before selecting prescription products

See Section 2 for information on nutritional supplements and patient fact sheets

Seek advice from / refer to a dietitian if there are concerns / queries that you cannot answer

Seek advice from the patient's clinical nurse specialist / specialist palliative care team for treatment of symptoms that you are not able to manage

Guidelines for using Nutritional Supplements

Introduction

These guidelines are designed to help you to understand when and what supplements to recommend for your patients as well as how they should be given. Nutritional supplements may not always be appropriate. Remember to refer to your local policy on using nutritional supplements if you have one.

When to use nutritional supplements

This section will help you to identify when nutritional supplements would be most appropriate.

The focus should be on enjoyment of nourishing foods in the first instance. However, some patients may find that a nutritional supplement helps where food intake is compromised, such as in cases of fatigue, swallowing difficulties or oral problems.

If a patient is not managing to take the prescribed amount of nutritional supplement, the dietitian should be contacted, unless the patient is approaching the last few days of life.

What nutritional supplements to use

Nutritional supplements are prescribed on form FP10, ACBS (Committee on Borderline Substances) endorsed.

Please refer to the current edition of the *British National Formulary (BNF)* for 'Contraindications to Use' for each product.

See Table 1 for recommended products.

Non-prescribed (over-the-counter) nutritional products

Over-the-counter products, such as Complan® (Complan Foods) and Build Up® (Nestlé) are available from supermarkets and chemists. These products are not available on prescription and do not have the same nutritional content as the prescribed nutritional supplements. *For more information on these and other nourishing drinks see Section 2, Focus on food leaflets and recipe ideas.*

Remember to offer patient fact sheets on storage and use of nutritional supplements and recipe ideas (see later in this section).

Range of Prescribed Nutritional Supplements

Table 1 shows the range of nutritional supplements available.

UHT milk-based supplements: Suitable as a sole source of nutrition. These supplements are recommended for patients who are able to take only a limited range of foods.

Fruit juice-based supplements: Not intended as a sole source of nutrition. Recommended for patients taking food, although can also be used in late palliative setting for patients unable to manage diet / UHT milk-based supplements. Useful for patients who do not like milk-based drinks. Not all are milk protein free. Refer to manufacturer details or seek advice from a dietitian.

Fresh milk-based supplements: In this range, only Complan® Shake is suitable as a sole source of nutrition for patients who are only able to take a limited range of foods. The other fresh milk-based supplements are not intended as a sole source of nutrition and are recommended in patients taking food, although they can also be used in a late palliative setting for patients unable to manage diet / UHT milk-based supplements. Patients may prefer fresh milk-based supplements.

Nutritional supplement starter packs: Useful particularly for finding out the patient's taste preferences. Contain an assortment of UHT milk-based and fruit juice-based supplements. The packs include information on recipes and serving suggestions.

EPA-enriched UHT milk-based supplements: These products are indicated for use by patients with pancreatic cancer. They should be used only under close medical supervision. They can be used as a sole source of nutrition or as a supplement to the patient's diet. Research has been done to assess the effectiveness of the omega-3 fatty acid eicosapentaneoic acid (EPA) and to see if it slows down the metabolic processes that lead to cachexia. Results to date have been mixed. If the decision is to trial these products it should be under the close supervision of a dietitian who is able to assess the appropriateness of the supplement, instruct on its use, and monitor and review the patient's nutritional status.

Other protein and energy nutritional supplements

These products should be prescribed only under the guidance of a dietitian, and include:

- fat supplements: Calogen® (SHS Ltd), Liquigen® (SHS Ltd)
- carbohydrate supplements: Super Soluble Maxijul® (SHS Ltd), Liquid Maxijul® (SHS Ltd), Polycal Powder® (Nutricia), Polycal Liquid® (Nutricia), Vitajoule® (Vitaflo)
- protein supplements: Promod® (Abbott), Protifar® (Nutricia), Vitapro® (Vitaflo)
- powdered soups: Vitasavoury® 200 and Vitasavoury® 300 (Vitaflo) high energy savoury supplement.

The dietitian will advise the patient on how to take these supplements mixed in certain foods and / or added to certain drinks.

Table 1: Types of nutritional supplements available on prescription*

UHT milk-based supplements	Fruit juice-based supplements	Fresh milk-based supplements
**Fortisip Bottle**® (Nutricia)<br>200 ml: 300 kcal, 12 g protein<br>*Fortisip Multifibre (with 4.5 g fibre), Fortifresh (yoghurt style)*	**Fortijuce**® (Nutricia)<br>200 ml: 300 kcal,<br>8 g protein	**Complan**® **Shake** (Complan Foods)<br>200 ml: 387 kcal,<br>16 g protein
**Ensure Plus**® (Abbott)<br>220 ml: 330 kcal, 14 g protein<br>Enrich Plus (with 2.5 g fibre),<br>*Ensure Plus Yoghurt Style (220 ml)*	**Enlive Plus**® (Abbott)<br>220 ml: 330 kcal,<br>11 g protein	**Enshake**® (Abbott)<br>310 ml: 600 kcal,<br>16 g protein
**Fresubin**® **Energy** (Fresenius)<br>200 ml: 300 kcal, 11 g protein<br>*Fresubin Energy Fibre Drink (with 4 g fibre)*	**Provide Xtra**® (Fresubin)<br>200 ml: 250 kcal,<br>7.5 g protein	**Calshake**® (Fresenius)<br>300 ml: 596 kcal,<br>11.6 g protein
**Clinutren**® **1.5** (Nestlé)<br>200 ml: 300 kcal, 11 g protein<br>*Clinutren 1.5 Fibre (with 5 g fibre)*	**Clinutren**® **Fruit** (Nestlé)<br>200 ml: 250 kcal,<br>8 g protein	**Scandishake**® (Nutricia)<br>300 ml: 598 kcal,<br>11.7 g protein
**Resource**® **Shake** (Novartis)<br>175 ml: 304 kcal,<br>9 g protein	**Resource**® **Fruits** (Novartis)<br>187 ml: 300 kcal,<br>8 g protein	

Nutritional supplement starter packs	EPA-enriched UHT milk-based supplements
**Enmix Commence**® (Abbott)<br>Contains 10 tetrapaks: 4 Ensure Plus, 2 Ensure Plus Yoghurt and 4 Enlive Plus	**ProSure**® (Abbott)—banana, orange, vanilla<br>240 ml: 300 kcal, 16 g protein, 1.0 g EPA
**Forti Range—FS010** (Nutricia)<br>Contains 4 Fortisips, 2 Fortifresh, 4 Fortijuce	**FortiCare**® (Nutricia)—cappuccino, orange and lemon, peach and ginger<br>125 ml: 200 kcal, 11.3 g protein, 0.75 g EPA

*Manufacturers constantly update their range of nutritional supplements and the nutritional composition of the products they supply. For further information seek advice from a dietitian.

STORAGE AND USE OF NUTRITIONAL SUPPLEMENTS

Storage

- Check the 'best before' date on the supplement drink before use
- Store drinks in a cool, dry place—eg a kitchen cupboard or pantry
- Once opened, drinks should be stored in the fridge and any unused portion discarded after 24 hours
- If the drink is left at room temperature it should be discarded after four hours

How to take your drink

- Milk-based and fruit-based supplement drinks are best taken sipped slowly through the day. Remember to discard any remaining drinks after four hours
- It is best to have these drinks after or between meals or in the evening, so that they do not make you feel too full to manage any food you might like
- Drinks are best served chilled, although they can also be heated gently if preferred
- Do not boil supplement drinks
- Drinks can be frozen into ice cubes, lollies or used to make desserts
- Nutritional supplements are available in a variety of flavours. Ask your GP if you would like to try some other flavours
- Drinks can be used in certain normal foods and drinks. Ask your GP / district nurse / dietitian for some recipe ideas

MACMILLAN
DURHAM
CACHEXIA
PACK
We work together to improve the experience of cachexia

Patient Fact Sheet

RECIPE IDEAS FOR FRUIT-BASED SUPPLEMENTS

Below is a list of fruit-based supplement drinks that may have been prescribed for you by your hospital doctor or GP:

- Enlive Plus® (Abbott Nutrition)
- Fortijuce® (Nutricia)
- Resource® Fruit Flavour Drink (Novartis)
- Provide Xtra® (Fresenius)
- Clinutren® Fruit (Nestlé)

You can use any of the above drinks in the following recipes:

Fruit Sparkler (serves 1)

- 1 carton fruit-based supplement, chilled—eg Enlive Plus, Fortijuce
- 70 ml lemonade, chilled

Mix and serve.

Apple and Blackcurrant Drink (serves 1)

- 1 carton apple flavour fruit-based supplement
- 50 ml blackcurrant cordial

Warm the apple flavour supplement gently in a pan. Do not boil. Stir in the blackcurrant cordial. Remove from heat and serve.

Fruit Juice Mixes (serves 1)

- 1 carton fruit-based supplement, chilled
- 100 ml fruit juice, chilled—eg orange juice with orange or pineapple flavour supplement; pineapple juice with pineapple or lemon and lime flavour supplement; apple juice with apple or pineapple flavour supplement

Mix the fruit juice with the fruit-based supplement and serve.

Fruit Jellies (serves 2)

- 1 carton fruit-based supplement (any flavour)
- 1 pack jelly (flavour to match the chosen fruit-based supplement)
- 275 ml hot water

Dissolve jelly in ½ pint hot water. Add fruit-based supplement to make 1 pint. Spoon into bowls and place in fridge until set. Serve with cream or ice cream.

Fruit Dessert (serves 1)

- 1 carton fruit-based supplement
- 3 rounded teaspoons cornflour
- A little water

Slowly heat the fruit-based supplement in a pan. Do not boil. At the same time, mix cornflour with a little water to form a paste. Rapidly stir cornflour paste into the fruit-based supplement and cook on a low heat until thickened. Spoon into a bowl. Serve hot or cold.

Further advice

If you have any questions about your supplement drink, please contact your dietitian, district nurse or GP.

MACMILLAN DURHAM CACHEXIA PACK
We work together to improve the experience of cachexia

RECIPE IDEAS FOR UHT MILK-BASED SUPPLEMENTS

Below is a list of some of the milk-based supplement drinks that may have been prescribed for you by your hospital doctor or GP:

- Ensure Plus® (Abbott Nutrition)
- Enrich Plus® (Abbott Nutrition)
- Fortisip® (Nutricia)
- Fortisip Multifibre® (Nutricia)
- Fresubin Energy Drink® (Fresenius)
- Fresubin Energy Fibre Drink® (Fresenius)
- Resource® Shake (Novartis)
- Clinutren® 1.5 (Nestlé)

You can use any of these drinks in the following recipes:

Banana Shake (serves 1)

- 1 carton banana flavour milk-based supplement, chilled
- 75 g vanilla ice cream
- Half a ripe banana

Blend ingredients together. Serve immediately in a tall glass and decorate with banana slices. Alternatively, try substituting banana with strawberry flavour drink and fresh strawberries.

Yoghurt Supreme (serves 2)

- 1 carton milk-based supplement, chilled—fruit flavour to match flavour of yoghurt
- 125 g thick and creamy yoghurt

Blend ingredients together. Serve immediately in a tall glass and decorate with fresh fruit sliced. Alternatively freeze and serve as an ice cream.

Hot Chocolate Delight (serves 1)

- 1 carton chocolate flavour milk-based supplement
- 2–3 tablespoons whipped cream
- A few drops of vanilla essence
- Chocolate flake and 1 teaspoon cocoa

Heat the supplement drink in a pan, but do not boil. Pour warm drink into a mug. Add a few drops of vanilla essence. Top with whipped cream. Sprinkle with cocoa and decorate with pieces of chocolate flake. Serve immediately.

Strawberry Whip (serves 2)

- 1 carton strawberry flavour milk-based supplement drink, chilled
- 1 packet instant whip
- 1–2 scoops ice cream—strawberry or vanilla flavour

Whisk ingredients together in a bowl. Place in a fridge until set.

Further advice

If you have any questions about your supplement drink, please contact your dietitian, district nurse or GP.

MACMILLAN
DURHAM
CACHEXIA
PACK
We work together to improve the experience of cachexia

Nourishing drinks

Try to have drinks which contain lots of calories, rather than tea and coffee. A cup of full cream milk provides the same calories as drinking eight cups of tea! Suggestions are given on some moderate and higher calorie drinks.

Low (less than 50 calories)
Water, tea, coffee, sugar free/ diet drinks, squash with water.

Moderate (50-100 calories)
Lemonade/cola, lager/beer, sherry, fruit juices, squash made with lemonade, semi and skimmed milk.

High (100-200 calories)
Cider, full cream milk, milkshakes/coffee/hot chocolate/malted drinks made with full cream milk, Build Up, Complan.

Practical tips to increase your calorie intake:

- Pour 2 tablespoons of double cream over some cake/icecream (extra 250 calories)
- Add 2 teaspoons of jam or honey to milk pudding (extra 100 calories)
- Melt grated cheese (a small match box size) into soup (extra 125 calories)
- Add a tablespoon of double cream to hot chocolate or malted drink (extra 125 calories)

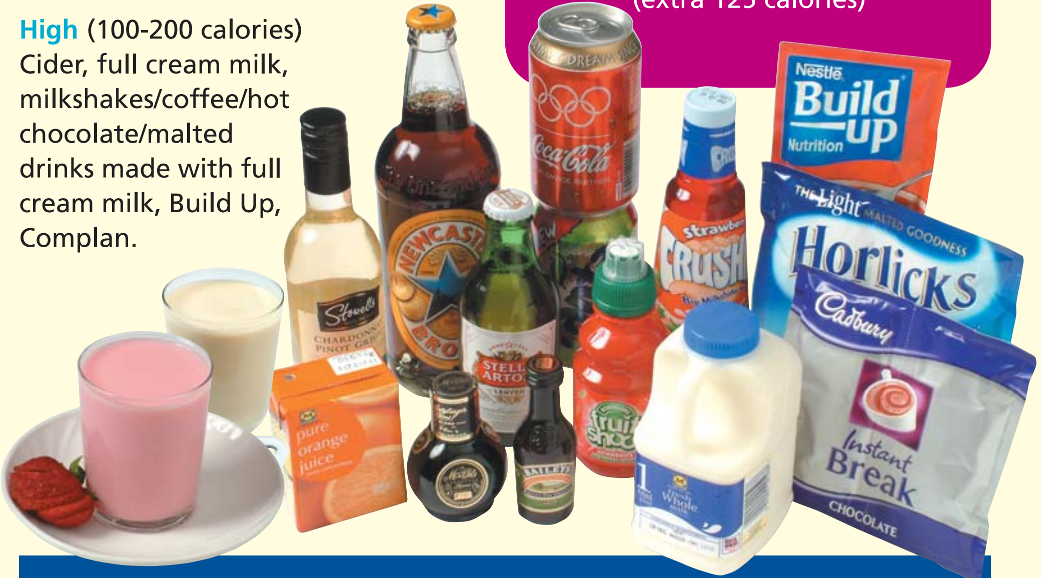

If your weight has decreased by more than 4lb (2kg) in a month, whilst following the recommendations in this leaflet, please contact the person who gave you this leaflet for further advice.

This document is available upon request in large print, audio cassette, and other languages. Please telephone 01388 455712 (South Durham) or 01207 594461 (North Durham).

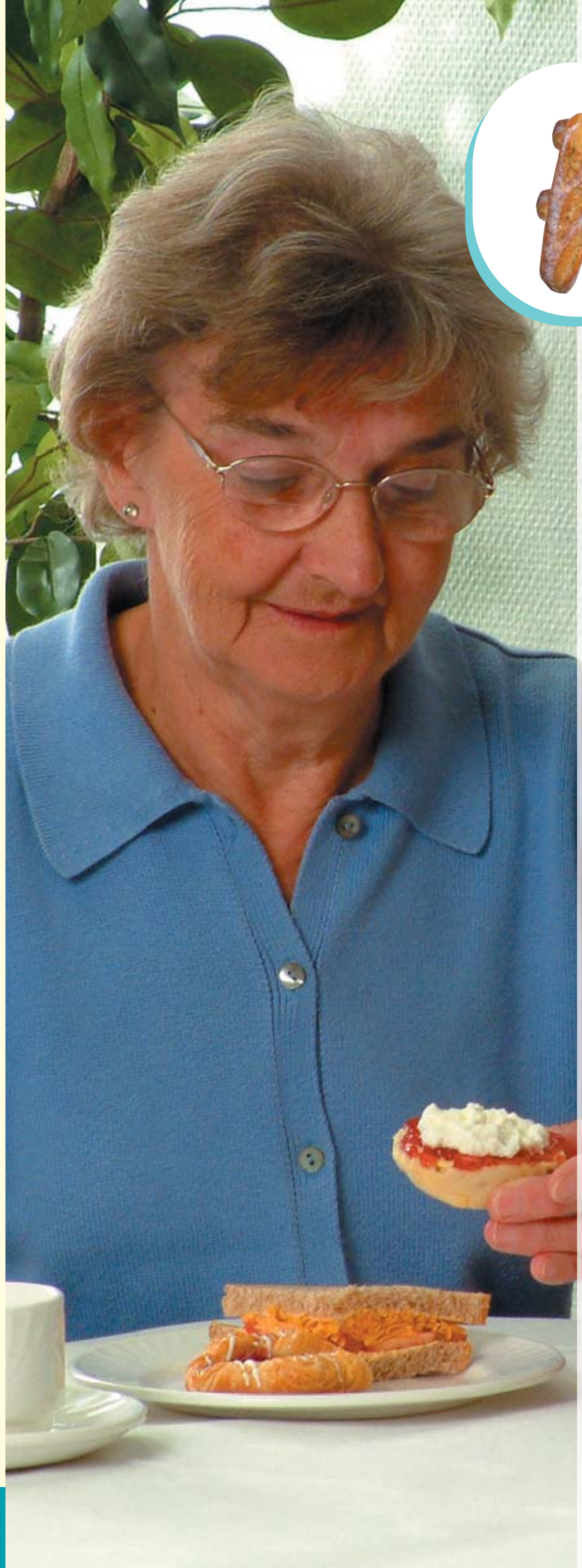

focus on Food

Adapting meals to prevent weight loss

focus on Food Is part of the Nutrition & Dietetic Services at South Durham, Darlington and North Durham

NHS

"Focus on Food" is funded by Darlington, Derwentside, Durham Dales, Durham and Chester le Street and Sedgefield Primary Care Trusts.

If you are experiencing a poor appetite, it is important that you still eat and drink adequately to prevent you from losing weight. This leaflet contains ideas for nourishing snacks, drinks and recipes for adapting your meals to increase the calorie and nutrient content. This is called "food fortification".

Suggestions for a poor appetite:

- Eat a "little of what you fancy"
- Eat three small meals a day
- Eat nourishing snacks between meals
- At each meal fortify one dish
- Drink nourishing drinks
- Always try to eat full fat fat versions
- Always try to use full sugar products, unless you have diabetes
- Monitor your weight at least once a month

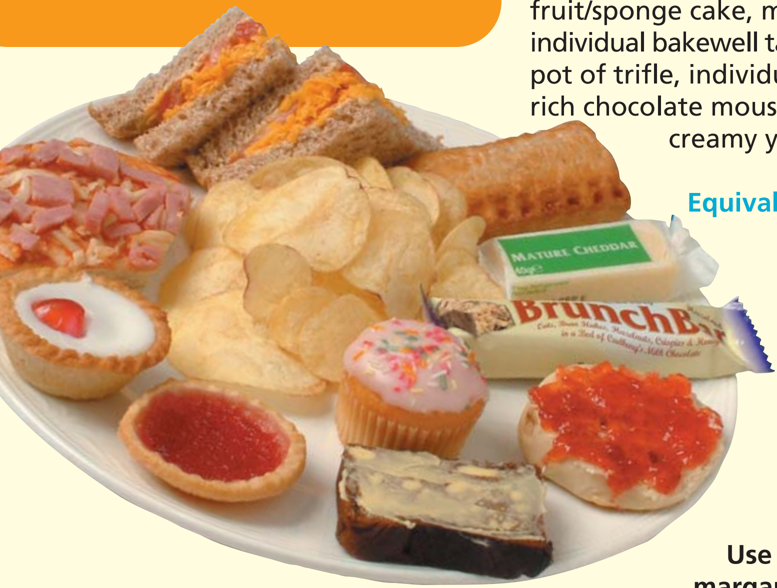

Nourishing snacks

Eat a small nourishing snack between meals. A plain biscuit is not a nourishing snack as it only contains 35 calories. A better choice of snacks are listed below, shown as the equivalent to eating 4, 6 or 8 plain biscuits.

Equivalent to 4 plain biscuits (140 calories):
A fairy cake, slice of malt loaf, half a hot cross bun, half a scone and jam, medium sausage roll, packet of crisps, scoop of ice cream, slice pizza, a cereal bar, handful of dried fruit, pot of custard or rice pudding, small matchbox cheese, ham/cheese sandwich (1 slice), jam tart.

Equivalent to 6 plain biscuits (210 calories):
A crumpet, half a teacake, slice of fruit/sponge cake, mini pork pie, an individual bakewell tart/fruit pie, doughnut, pot of trifle, individual cheesecake, pot of rich chocolate mousse, pot of thick and creamy yoghurt.

Equivalent to 8 plain biscuits (280 calories):
Small slice flapjack, 2 slices malt loaf, half a scone, jam and cream, 2 crackers and cheese, a danish pastry, chocolate bar, handful of peanuts (50g).

Use generous amounts of margarine/butter on snacks.

High calorie, protein (fortified) diets

Fortifying is when small quantities of everyday foods, such as cream, milk powder or butter are added to a food dish to increase the nutritional content, without increasing the portion size. This means every mouthful you eat will be full of nourishment.

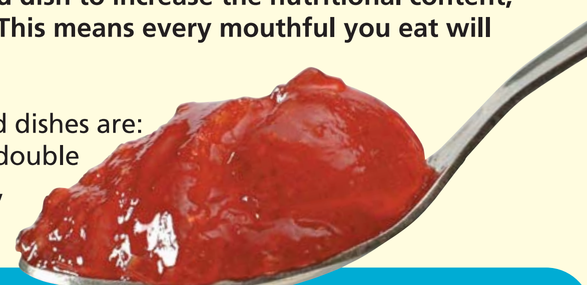

Everyday foods you could add to food dishes are: milk powder (1 heaped tablespoon), double or whipping cream (1-2 tablespoons), butter or margarine (1 teaspoon) or jam (1 teaspoon). When adding milk powder to dishes, mix it into a paste with some cream or milk, otherwise it may not mix well.

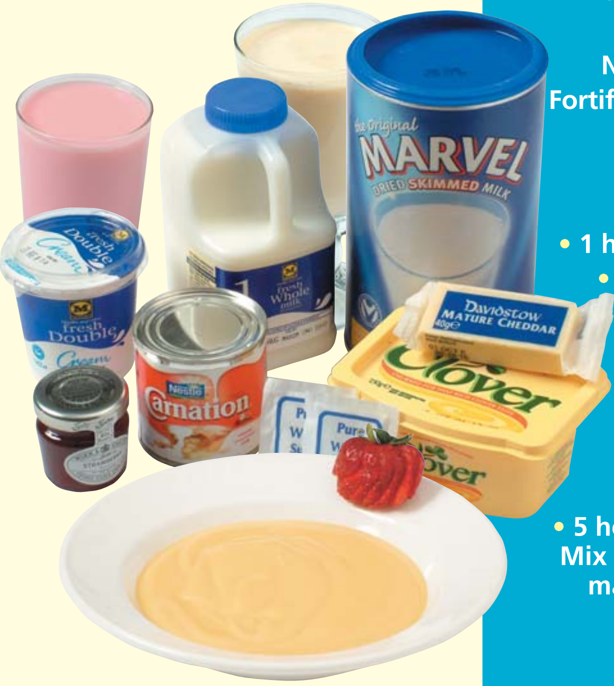

Fortified custard/porridge/milk pudding

- 1 ladle of normal custard/porridge/milk pudding
- 1 heaped tablespoon milk powder
- 2 tablespoon double cream

Normal versions: 150 calories
Fortified versions: average 450 calories

Fortified Soup

- 1 ladle of normal soup
- 1 heaped tablespoon milk powder
- 2 tablespoons double cream

Normal soup: 80 calories
Fortified soup: 350 calories

Fortified milk

- 1 pint full cream milk
- 5 heaped tablespoons milk powder

Mix milk powder with some milk to make a runny paste and add to remainder of pint.

Standard milk: 375 calories
Fortified milk: 630 calories

Adapting meals to prevent weight loss focus on Food

To help prevent weight loss it is important to have TWO nourishing drinks a day from this leaflet. Each drink (200mls) contains at least 300 calories and 10g protein, which is similar to the prescribable nutritional drinks, such as Fortisip, Ensure Plus, Fresubin.

Tips for nourishing drinks:

- Always use full cream milk.
- Always use double or whipping cream not single cream.
- Mix milk powder to a runny paste with milk or cream to help it mix well.
- **Information for diabetics:** Milkshake recipe use pureed fruit or drink with a meal or snack. Hot chocolate recipe use “Highlights” or “Options”.

Focus on Food milkshake

- 200mls milk
- 2 heaped tablespoons milk powder
- Milkshake syrup or powder to taste
- Calories per serving: 300

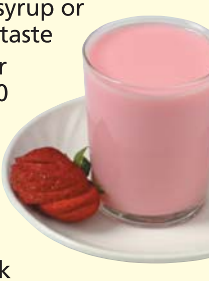

Yoghurt drink

- 150mls milk
- 1 heaped tablespoon milk powder
- 1 pot (150g) thick and creamy yoghurt
- Calories per serving: 300

Pineapple or banana yoghurt drink

- 300mls milk
- 1 heaped tablespoon milk powder
- 1 pot (150g) thick and creamy yoghurt
- 3 pineapple rings or 1 ripe banana
- Liquidise all the ingredients for 15 seconds. Serve chilled
- Calories per serving: 300 pineapple, 300 banana

Nourishing hot chocolate

- 150mls milk
- 1 heaped tablespoon milk powder
- 1 teaspoon coffee powder
- 2 tablespoons of cream
- Calories per serving: 350

Nourishing coffee

- 150mls milk
- 1 heaped tablespoon milk powder
- 1 teaspoon coffee powder
- 2 tablespoons of cream
- Calories per serving: 350

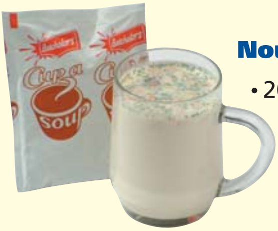

Nourishing Cup-a-Soup

- 200mls milk
- 1 heaped tablespoon milk powder
- 1 packet of Cup-a-Soup
- Calories per serving: 300

Nourishing liqueur drink

- 125mls milk
- 1 heaped tablespoon milk powder
- 2 tablespoons of cream
- 4 tablespoons cream liqueur (e.g. Baileys)
- Calories per serving: 500

Nourishing hot chocolate or malted drink

- 150mls milk
- 1 heaped tablespoon milk powder
- 3 teaspoons hot chocolate powder or malted drink powder (e.g. Ovaltine, Horlicks)
- 2 tablespoons cream
- Calories per serving: 450

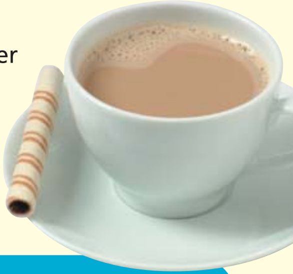

Further tips for adding extra calories

- Add a measure (25mls) of sherry, brandy, or liqueur to hot drinks (extra 55 calories)
- Add 1 tablespoon double cream (extra 135 calories)
- Add 1 scoop of vanilla icecream to cold drinks (extra 115 calories)
- Build Up or Complan are also useful options as high calorie nourishing drinks
- If you find recipes too creamy replace 1 tablespoon cream with 4 tablespoon full cream milk, this will reduce the calories by 100

This document is available upon request in large print, audio cassette, and other languages. Please telephone 01388 455712 (South Durham) or 01207 594461 (North Durham).

Exercise Algorithm 1

Patient wants exercise advice

Check for complicating factors:
- Cardiac (risk from increased heart rate or change in blood pressure / postural hypotension)
- Recent onset of back pain
- Recent onset of neurological deficit
- Limb pain on active movement
- Any unexplained fall in last two weeks
- Patient requires assistance of two people to stand or transfer
- Persistent dizziness

Complicating factor present

Discuss with relevant clinician, specialist nurse or physiotherapist before prescribing exercise

Complicating factor absent

Give copy of exercise programme 1 and draw patient's attention to general safety advice

Instruct patient to sit upright in chair or on edge of bed with aid for balance nearby (if required)

Go through each exercise in turn, ensuring that the patient understands:

- Start and finish positions
- Frequency of sessions per day / week
- Repetitions of exercises per session
- Precautions

Able to perform all exercises and patient feels confident to continue programme independently

Go through instruction at bottom of programme, regarding walking practice—reinforce safety. It is crucial that this programme does not become another task to 'fail'

Make sure patients start with very low repetitions and builds slowly

Leave patient with exercise programme and instruct them on relevant follow up

Unable to perform one or more exercise safely or without pain

Score out (on patient's exercise programme) any exercise that is unsafe and refer to a physiotherapist

If there are serious concerns or the patient finds all exercises very difficult, do not leave patient with exercise programme; refer to physiotherapist

Patient Fact Sheet

EXERCISE PROGRAMME 1

Things you need to know before starting to exercise:

- You are in charge of your exercise programme
- Even small amounts of exercise can be beneficial; it is not helpful to put yourself under too much pressure
- You can decide when and by how much to increase and decrease the number of repetitions of each exercise
- If you develop pain through exercising, stop and seek advice
- If you feel unsafe or at risk in any of the start positions, seek advice
- Rest is important as well as exercise
- If any symptoms worsen during or just after exercising, stop and seek advice
- These exercises may seem ‘simple’, but they can be effective in strengthening the muscles you need to stand and walk

Things you need to know when exercising:

- For all exercises (shown on the next page), sit upright in a chair (if possible one that you can independently rise from)
- Repetitions:
    - Repeat each exercise 2–15 times. Try out a low number and then build slowly. You should not be exhausted after each set of exercises
    - Repeat the whole programme 1–3 times per day. Try out a low number then build slowly
- If you are able to walk safely without the assistance of another person (OK to use a mobility aid), it is very important to include walking practice in your daily exercise programme. Choose a distance that is manageable and in a safe environment and include it in your exercise programme. If you require advice about more demanding exercise, please seek referral to a physiotherapist

Exercise 1	Exercise 2	Exercise 3
**Ankle bending**	**Knee straightening**	**Sit–stand**
**Start** 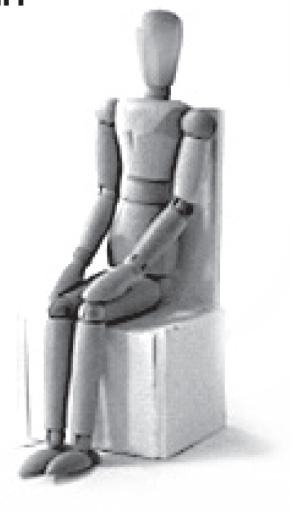	**Start** 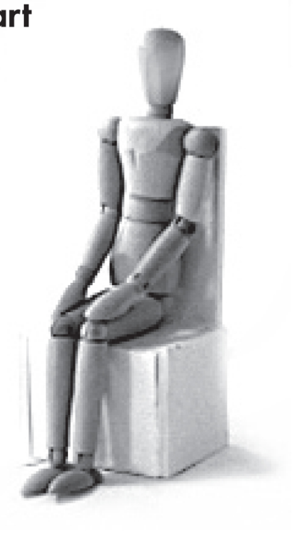	**Start** 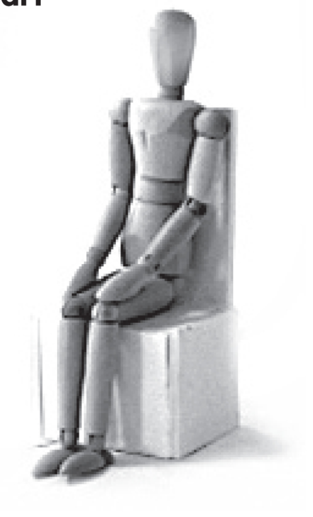
**Finish** 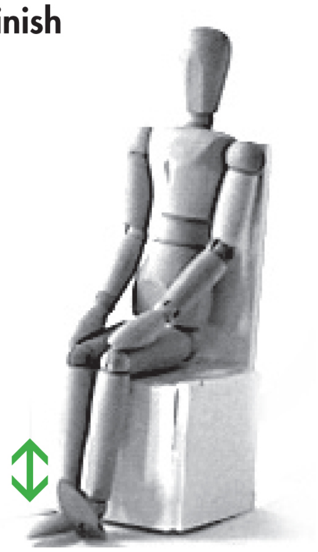	**Finish** 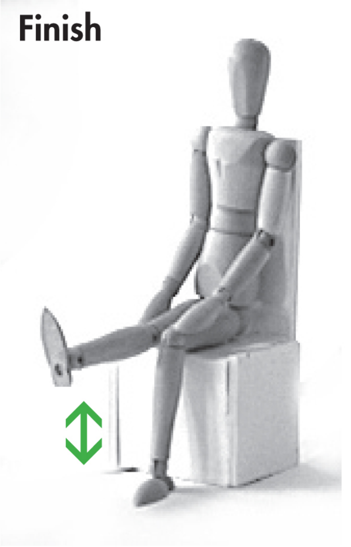	**Finish** 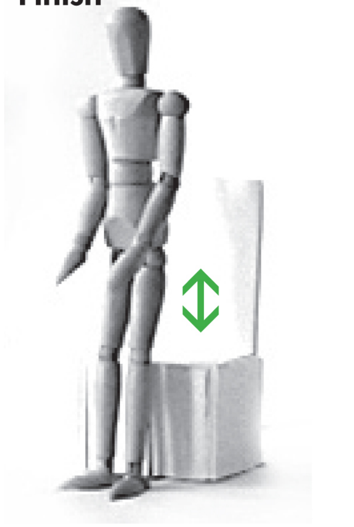
Keep foot up for three seconds. Slowly return to start position	Hold leg straight for three seconds. Slowly return to start position	Lean forwards and rise. Hold onto something for balance (do not attempt if you are at risk of falling). It is OK to push up with your arms. Sit down slowly

MACMILLAN
DURHAM
CACHEXIA
PACK
We work together to improve the experience of cachexia

Exercise Algorithm 2

Patient / carer wants exercise advice

↓

Is patient able to follow instructions?

Yes

Give patient / carer Exercise Programme 2

↓

Go through each exercise in turn, ensuring that the patient understands:

- Start and finish positions
- Repetitions of exercises per session
- Frequency of sessions per day / week
- Precautions

It is crucial that this programme does not become another task to ‘fail’; make sure that patients start with very low repetitions and build slowly

No

If a carer can / wishes to assist, teach gentle passive limb movements:

- Hip / knee bend
- Ankle bend
- Elbow bend
- Finger straightening

These movements should be slow and gentle; **never** push against resistance or cause pain

Patient Fact Sheet

EXERCISE PROGRAMME 2

These exercises should be done lying flat on your back, propped up in bed or sitting in a chair with your legs raised on a stool.

Before beginning the programme, take three slow, deep breaths.

Exercise 1

Move your toes

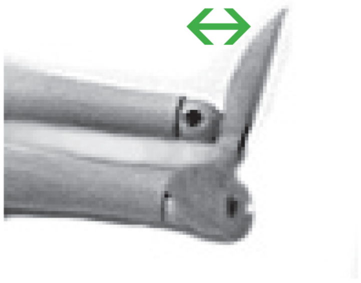

Exercise 2

Ankle circles:

- Put your legs out straight
- Make a circle with your toes so that your foot moves
- Do this exercise with one foot, slowly, in one direction and then in the other direction
- Repeat with the other foot

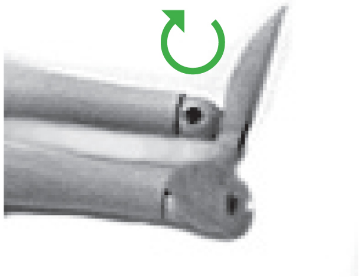

Exercise 3

'Static Quads' (tightening your thigh muscles):

- Keep your knee straight throughout this exercise
- Bring your toes up towards you (so that your ankle bends)
- Push the back of your knee down into the bed / chair— the muscle of the thigh should tighten
- Hold this position for three seconds
- Release
- Repeat

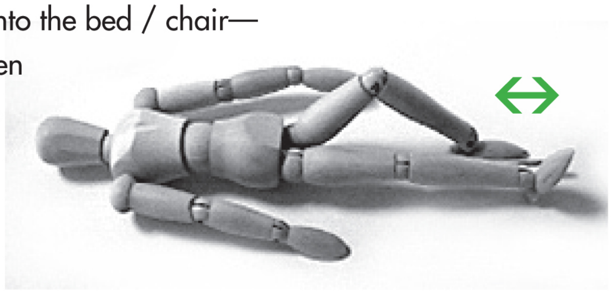

**Exercise 4**<br>Arm raise:<br>• Lie on your back or propped up in bed<br>• Raise one arm above your head<br>• Lower slowly<br>• Repeat with the other arm<br>• If it is easier, interlink your fingers and raise your arms together	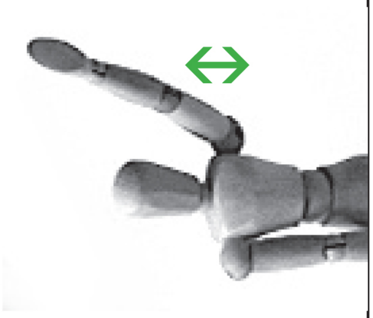

All of these exercises should be pain free. If you experience pain or a worsening of any of your symptoms during exercise, stop and seek advice.

Repetitions:

- Repeat each exercise 2–15 times. Try out a low number and then build slowly
- Repeat the whole programme 1–3 times per day

MACMILLAN DURHAM CACHEXIA PACK
We work together to improve the experience of cachexia

Patient Fact Sheet

PACING AND DAILY ACTIVITIES

This leaflet is aimed at individuals experiencing tiredness / fatigue that makes daily activities difficult (especially for those who have ’good days’ and ‘bad days’).
Use this leaflet alongside advice on managing your diet.

Often when suffering with cancer-related weight loss there is an imbalance between energy going into your body and the energy that you use in normal activities.

Energy in (food and drink calories) ***LESS THAN*** *energy out (used by muscles during activity).*

This imbalance is one cause of cancer-related fatigue. The tiredness / fatigue is not relieved by rest and is characterised by sensations of exhaustion and loss of motivation to perform activities (sometimes severe).

Planning ahead, pacing and listening to your body may help to offset this fatigue.

Planning ahead

Especially when you identify tasks that cause extreme tiredness.

- Ask others (family, friends, neighbours) for help with daily activities
- Plan your day so that you have time to rest and do the things you want to do
- Spread tasks out over the week
- Use equipment to help save energy (for instance sitting or perching instead of standing). An occupational therapist can help you find ways of saving energy
- Organise tools and materials in work area (kitchen etc). Store items within easy reach

Pacing activities

Many activities do not need to be done quickly or all in one go.

- The principle of successful pacing of activities is avoiding exhaustion
- Try going more slowly
- Take planned breaks before you become tired
- Save some tasks for later in the day or even the next day
- It is easier to keep some small reserves for the next task than it is to build those reserves after they are depleted (exhaustion)

Listening to your body

When we perform everyday tasks, we tend to be in 'automatic' mode, but this is not very useful to those who experience debilitating fatigue and tiredness. To successfully pace yourself and plan ahead, you must learn to 'hear' what your body is telling you before, during and after activities.

Try looking closely at one particular task (shaving, dressing, bathing, etc):

- At what point does the feeling of significant tiredness begin?
- Are there natural breaks in the task?
- Could you sit down to do the activity?
- Is there someone who could help you? (Talk to your GP, district nurse or social worker)

For expert advice and assessment related to pacing and equipment, request referral to an occupational therapist.

Information is also available from Cancerbackup: freephone 0808 800 1234, website www.cancerbackup.org.uk.

Management Strategies Guidance

This section provides advice on how best to manage the common symptoms encountered in ACS. These have been proposed using the available evidence base (often weak), current best practice or experience during the Macmillan Durham Cachexia Project *(see Section 6, Evidence base)*. In some situations, there are a number of possible therapeutic options. The suggestions given here are not exhaustive and are intended to offer ideas only. Local policy, prescriber preferences and patient characteristics may require alternative approaches not listed here.

Prescribing Guidance

Please note the following when using the drugs recommended in this section*:*

- Always try and reverse causes of symptoms first.
- Pharmacological options should be chosen on an individual basis, taking into consideration: patient choice, contraindications, current medication, side-effect profile etc. Drugs with a star (*) after their name are not intended for long-term use unless clearly indicated and reviewed. All prescribed drugs need review at regular intervals.
- Refer to local palliative care guidelines (often available for pain and nausea / vomiting).
- Responsibility for drug choices remains with the prescriber.
- Many drugs are not suitable for long-term use and should be reviewed at regular intervals. These include:
    - corticosteroids
    - dopamine antagonists (including metoclopramide)
    - thalidomide
    - fluconazole
    - loperamide.
- Responsibility for ongoing review of these drugs should be clearly communicated.
- Stop a drug if it gives no benefit.

The Macmillan Durham Cachexia Pack does not contain full prescribing information for medicines. Healthcare professionals should consult the relevant Summary of Product Characteristics (SPC) and be aware that some recommendations may be outside of the product licence.

Patient / Carer Fact Sheet

MOUTH CARE

Looking after your mouth is an important part of helping your symptoms such as dry mouth, sore mouth and taste changes. Some ideas of ways you can do that are listed below. It is always important to be gentle with your mouth.

Brushing teeth

Brushing your own teeth: Your toothbrush should be small headed, with medium texture, nylon filaments. The brush should be changed every three months. Brushes are better than sponges for effective tooth cleaning. Electric toothbrushes are not better than manual toothbrushes.

Brushing someone else's teeth: Put a towel over the patient's clothes to protect them from splashes and sit them comfortably. Their head should be well supported. Stand behind the patient and hold the lower jaw all the time. Brush gently, being careful to avoid injuring the gums, cheeks and tongue.

Toothpaste: Use of toothpaste is not as important as the actual brushing. Choose a toothpaste with 1000 ppm fluoride. If the mouth is sore, you can try Kingfisher® natural toothpaste or tea tree oil toothpaste.

Mouthwash: Chlorhexidine mouthwash can be useful to control plaque and keep the mouth clean and healthy. This is useful to stop infections and sore mouths developing. It is also helpful for people who are too unwell to have effective tooth brushing. Use 10 ml twice daily (diluted 50% with warm water if it stings) after brushing the teeth.

Denture care

Removal: Take dentures out and clean daily. If you are helping to take someone else's dentures out, you should: protect the patient's clothing with a towel, support their head, remove lower dentures first—rotate gently then remove (never pull straight out).

Cleaning: Dentures should be washed with liquid soap and rinsed thoroughly. If the dentures are infected, leave them to soak in a solution of dilute Milton®.

Storage: Store dentures in water, ideally overnight, but always for a minimum of one hour per day.

Fitting: Dentures last 5–10 years at the most. It is important that dentures fit well; illness can affect the fit, so they should be checked at regular intervals. If dentures are damaged, do not use.

Keeping your mouth comfortable

Cleaning: Clean your teeth (and tongue) every four hours during the day as above.

Dryness: You can take sips of water to keep your mouth moist, but you should be careful if you tend to feel full quickly that water is not preventing you from having something nourishing. Ask your doctor about mouth gels or sprays to help. Use sugar-free chewing gum or sweets to help stimulate the production of saliva.

Soreness: Ask your doctor about mouthwashes. See toothpaste advice above.

Further information on mouth care is available from Cancerbackup:
freephone 0808 800 1234, website www.cancerbackup.org.uk.

MACMILLAN
DURHAM
CACHEXIA
PACK
We work together to improve the experience of cachexia

Management Strategies

- Always try to reverse causes of symptoms first.
- Pharmacological options should be chosen on an individual basis, taking into consideration: patient choice, contraindications, current medication, etc. Drugs with a star (*) after their name are not intended for long-term use unless clearly indicated and reviewed.
- All prescribed drugs need review at regular intervals.
- Refer to local palliative care guidelines (often available for pain and nausea / vomiting).
- Always seek advice if unclear.

No appetite	Weight loss	Feeling full quickly
**Non-pharmacological options**<br><br>Facilitate food intake<br>*(refer to Section 2):*<br>• Extra foods—eg nourishing snacks, drinks and desserts<br>• Eat 'little and often'<br>• Eat 'what you want, when you want'<br>• Use small plates<br>• Fortify foods and drinks<br>• Consider oral nutritional supplements<br><br>*For family / carer tension / anxiety see leaflets provided in Section 5*<br><br>**Pharmacological options**<br><br>• Dexamethasone* 4 mg mane after food; review weekly<br>• Megestrol acetate 160 mg tds (+/- ibuprofen 400 mg tds after food); review every two weeks	**Non-pharmacological options**<br><br>Facilitate food intake<br>*(refer to Section 2):*<br>• Extra foods—eg nourishing snacks, drinks and desserts<br>• Eat 'little and often'<br>• Eat 'what you want, when you want'<br>• Use small plates<br>• Fortify foods and drinks<br>• Consider oral nutritional supplements<br>• Assess pressure risk<br><br>*For family / carer tension / anxiety see leaflets provided in Section 5*<br><br>**Pharmacological options**<br><br>• Megestrol acetate 160 mg tds; review every two weeks<br>• EPA supplement: Prosure® (*see Section 2, Range of prescribed nutritional supplements*) or Omacor® 1 g od to bd<br>• Thalidomide* 50 mg bd (unlicensed product, company registration required)	**Non-pharmacological options**<br><br>Facilitate food intake<br>*(refer to Section 2):*<br>• Extra foods—eg nourishing snacks, drinks and desserts<br>• Eat 'little and often'<br>• Eat 'what you want, when you want'<br>• Use small plates<br>• Fortify foods and drinks<br>• Consider oral nutritional supplements<br><br>*For family / carer tension / anxiety see leaflets provided in Section 5*<br><br>**Pharmacological options**<br><br>• Metoclopramide* 10 mg tds before food; review within one week and fortnightly thereafter<br>• If this dose is inadequate or patient is already taking the drug, increase dose to maximum of 120 mg per day in divided doses

<table>
<tr><th>Nausea / vomiting</th><th>Diarrhoea</th><th>Constipation</th></tr>
<tr><td>Consider cause and treat if possible

Non-pharmacological options

• Avoid triggers where possible (eg smells)
• Acupressure wrist bands
• Leaflets (Section 5, PILs 7 and 9)

Pharmacological options

If complete bowel obstruction excluded:

• Metoclopramide* 30–60 mg per day by injection if needed (can be given in subcutaneous syringe driver)

If this dose is inadequate or the patient is already taking the drug:

• Increase dose to maximum of 120 mg per day
• Use other anti-emetics as per local palliative care advice</td><td>Ensure medical staff are aware of this symptom; if not, refer

Non-pharmacological options

• Increase fluid intake
• Review diet
• Low fibre foods—eg white bread / cereals, pasta, rice

Pharmacological options

• Rehydration sachets; one after each loose bowel motion

Only after medical review

• Loperamide*, 4 mg stat, 2 mg after each loose bowel motion (maximum 16 mg per day)</td><td>Non-pharmacological options

• Increase fluid intake
• Increase fresh / frozen / dried fruit and vegetable intake
• Increase mobility / exercise (Sections 1 and 3)
• Increase intake of cereals, bread, rice and pasta

Above likely to be inappropriate for more poorly patients

Pharmacological options

Review all of the following drugs within one week and then 1–2 weekly thereafter.

Motion soft, but unable to pass:

• Senna 15 mg nocte

Motion hard, but able to pass:

• Docusate 200 mg nocte / bd

• If inadequate response to single agent, try combination of senna and docusate

If already tried above:

• Macrogols (eg Movicol® / Idrolax®) 1–2 sachets daily;</td></tr>
</table>

Mouth sores	Dry mouth	Altered taste
Examine mouth<br><br>Offer Patient / carer fact sheet: Mouth care *(Section 4)*<br><br>**Non–pharmacological options**<br><br>• Avoid acidic / salty / spicy foods and drinks<br>• Avoid dry, rough texture foods<br>• Cold drinks may be more soothing<br><br>**Pharmacological options**<br><br>1. Oral candida<br>• Fluconazole 50 mg mane, review after seven days; if candida still present continue 50 mg mane for a further seven days; if still present, swab and send for culture and sensitivity analysis<br>2. Ulcers (five-day course):<br>• Adcortyl in Orabase®, apply to ulcer qds, do not rub in<br>• Corlan® pellets, apply to ulcer qds<br>3. Cause unknown<br>• Benzydamine 0.15% mouthwash 15 ml, 1–3 hourly (dilute with warm water if stings)	Examine mouth<br><br>Offer Patient / carer fact sheet: Mouth care *(Section 4)*<br><br>**Non–pharmacological options**<br><br>• Moisten foods with sauces / gravy<br><br>Saliva stimulants:<br><br>• Sugar-free chewing gum chewed regularly, after meals<br>• Sugar-free sweets<br>• Sips of fluid offer temporary relief only<br><br>*See leaflets in Section 5*<br><br>**Pharmacological options**<br><br>• Pilocarpine 5 mg tds; review every two weeks<br><br>Saliva substitutes (combination or separately):<br><br>• Bioténe Oralbalance® gel, rub on hand then apply qds and prn to mouth and lips<br>• AS Saliva Orthana® spray, qds and prn	Examine mouth<br><br>Offer Patient / carer fact sheet: Mouth care *(Section 4)*<br><br>**Non–pharmacological options**<br>• Choose favourite foods<br>• If taste decreased / absent, add flavours—sugar, salt, herbs, spices<br>• If taste increased, remove / avoid the above<br><br>*See leaflets in Section 5*<br><br>**Pharmacological options**<br><br>Check renal function then trial of:<br><br>• Solvazinc*® one tds; review every two weeks

Problems swallowing	Bothered by smells	Fatigue
Refer to medical staff if they are unaware of problem. Patient may need referral to speech and language therapist<br><br>Offer Patient / carer fact sheet: Mouth care *(Section 4)*		Consider cause:<br><br>• Anaemia; renal dysfunction; hypercalcaemia; hypothyroidism (check bloods)<br>• Depression<br>• Sedative drugs (review)<br>• Sleep pattern
**Non-pharmacological options**<br><br>• Modified texture diet (eg soft, moist foods)<br>• Delivered meal providers (eg Meals on Wheels may supply appropriate diets)<br><br>**Pharmacological options**<br><br>Consider candida *(see 'Mouth sores')* and oesophagitis<br><br>• Metoclopramide* 10 mg tds may be useful (see 'Feeling full quickly')	**Non-pharmacological options**<br><br>• Cold foods (cereals, milk puddings, sandwiches, crackers)<br>• Advise patient to avoid cooking for themselves<br><br>Family / carer tension / anxiety:<br>• If smells are bothering the patient, advise them to leave the food preparation area<br>• Open all windows and close all doors while cooking<br>• Try neutralising air fresheners (but stop if aggravating)<br><br>*See leaflets in Section 5*	**Non-pharmacological options**<br><br>• Realistic goals and pacing exercises *(Section 3 'Pacing and Daily Activities' leaflet)*<br>• Appropriate exercise programme *(Sections 1 and 3)*<br>• Promote good sleep at night (Cancerbackup helpline / website)<br>• Equipment provision (may need occupational therapy / physiotherapy assessment)<br>• Delivered meal providers (eg Meals on Wheels)<br>• 'Ready meals' to heat<br>• Nourishing drinks may be easier than a meal<br>• Nutritional supplements *(Section 2)*

Common questions

Which foods contain protein and are easy to eat?

Fish, eggs, baked beans, lentils, milk, yoghurt, cheese.

What foods contain energy and are easy to eat?

Cakes, sweets, chocolate, bananas, fresh fruit juice, rice, pasta.

Are there any foods that contain both protein and energy?

Cereals with milk (eg Weetabix®), scrambled egg with buttered toast, milk puddings, milkshakes, smoothies.

Further information

www.mru.nursing.soton.ac.uk/patient.html
(from November 2006)

Cancerbackup publishes free information about using supplements to fortify meals, including recipes. Call **020 7696 9003** or visit **www.cancerbackup.org.uk**

WE ARE MACMILLAN. CANCER SUPPORT

Macmillan Cancer Support
89 Albert Embankment
London SE1 7UQ
Tel 020 7840 7840
Fax 020 7840 7841
www.macmillan.org.uk

© Macmillan Cancer Support, June 2006. Registered charity number 261017.
Isle of Man charity number 604.

© University of Southampton, March 2006. This leaflet has been prepared using evidence from many sources including research with 200 people living with advanced cancer.

MAC5844 A

MAC5884 A

EATING WELL

Information to help people living with cancer

University of Southampton | School of Nursing and Midwifery

WE ARE MACMILLAN. CANCER SUPPORT

Are you living with advanced cancer?

If so, you may be eating less than before, and be worried about eating well.

How you eat well when you have cancer can be different to how you ate before. It's about making the most of the appetite that you have. This leaflet can help you.

You can also talk to your nurse or dietitian who will be able to assess your eating habits. They may be able to help you find ways to eat well.

Tips to help you eat well

What you eat can make a difference to how you feel. Eating well can be important to feeling good.

How do I know if I am eating well?

A diet that's high in energy and protein is healthy for someone who has lost weight or who has a small appetite. If you are making the most of your appetite by eating as much protein and energy as you are able to, then you are eating well.

How can I make the most of my appetite?

Think about the sort of foods you eat, rather than the amount that you eat. Try to think about the foods you can eat that contain energy and protein. It may be possible to increase the amount of these in your diet without increasing the size of your meals.

The simplest way to eat well is to think about what you enjoy eating and are able to eat. Then choose to eat the foods you prefer that contain the most protein and energy.

Are there other ways I can make the most of my appetite?

You could consider fortifying your diet by increasing the nutrients in your food, without increasing the amount you eat. You can fortify your food in two ways:

1. You can add foods that contain a lot of protein or energy to others. For example, add two tablespoons of double cream to a bowl of soup. Or melt butter on vegetables to add energy. Another idea is to add four tablespoons of powdered milk to a pint of full cream milk before using it in puddings or sauces.
2. You can add commercially prepared supplements to food or drink. (Supplements are special powders or liquids that contain a lot of protein, energy or both.) For example, add neutral Build-Up or Complan to gravy, sauces and soups. Alternatively, you can add two to four teaspoons of a high energy powder, such as Maxijul or Polycal, to drinks.

I want to have as much energy as possible

High energy foods include rice, pasta, bread, biscuits and anything containing sugar or fat. You may find it helpful to have a high energy snack before an activity. For example, eat a square of chocolate or take a drink of pure fruit juice before getting dressed.

I want to eat foods that will help me keep my muscle strength

Foods that can help you to keep your muscle strength contain protein. Cheese, fish, chicken, eggs, milk, yoghurt, nuts and minced meat are all sources of protein that are easy to eat.

I've heard that there are special diets for people with cancer

You could decide to take a special diet to help you feel good. However, there is no scientific evidence that organic food or any special cancer diet can prevent the spread of cancer or prolong life.

Why do many people with cancer have eating problems?

There are many reasons why eating habits can change. Treatments and medicines can affect how someone feels about eating. Feeling sick, having diarrhoea or constipation can stop someone eating. Feeling low, tiredness or pain can make eating difficult.

Even if the cancer is not causing any other problems, it can affect eating habits. This is because the body's response to the cancer can include a loss of appetite.

Because cancer changes the way the body uses food, it is possible to lose weight even if eating normally.

What can I do?

You can also join the Macmillan Eating Web Discussion Group where patients and carers share their experiences of living with eating difficulties and what they have found helpful.

Read the tips in this leaflet to find out what others have found helpful when living with someone with eating difficulties.

Further information

Have you got a small appetite?
(2001) British Dietetic Association

www.mru.nursing.soton.ac.uk/carer.html
(from November 2006)

WE ARE MACMILLAN. CANCER SUPPORT

Macmillan Cancer Support
89 Albert Embankment
London SE1 7UQ
Tel 020 7840 7840
Fax 020 7840 7841
www.macmillan.org.uk

© Macmillan Cancer Support, June 2006. Registered charity number 261017.
Isle of Man charity number 604.

© University of Southampton, March 2006. This leaflet has been prepared using evidence from many sources including research with 200 people living with advanced cancer.

MAC5844 B

MAC5884 B

HELPING SOMEONE LIVE WITH CHANGES IN THEIR EATING HABITS

Information to help people living with cancer

University of Southampton | School of Nursing and Midwifery

WE ARE MACMILLAN. CANCER SUPPORT

Do you know someone with advanced cancer who is having problems eating?

If so, you may have one or more of the following concerns.

Tips to help you live alongside someone with eating changes

I find living with someone whose appetite changes is very frustrating

The amount someone with cancer can eat can change from day to day for no reason. Their likes and dislikes may even change from hour to hour.

It can be best to encourage the person to eat on the days and at the times that they feel most able to. This may not coincide with the times you normally eat. You might find it helpful to keep a stock of different foods so that you can offer them food at any time of the day.

Consider keeping tinned foods and pre-prepared frozen meals ready for the time the person feels able to eat. Quick food can be as good for them as a meal that takes a long time to prepare.

What should I do when they can't eat much?

It can be a good idea to offer small servings on a small plate. A portion that is too large can put someone off their meal. Try offering just a small amount of one of the foods other people are eating.

Soft food is easiest to eat because it does not need a lot of chewing. Try offering porridge, Weetabix®, soups, custards, bananas, yoghurts or milk-based drinks.

You could also keep snacks on hand. Snacking on nuts, crisps, toast, chocolate or biscuits between meals can be a good idea for someone who is only able to eat a little at mealtimes.

I find it difficult to know what to prepare

Try asking what they feel like eating. Try experimenting with different tastes. Often people who are having difficulty eating find that they prefer cold food or foods they would not have eaten in the past.

Drinking high energy and protein fluids, such as milk shakes, smoothies, BuildUp® or Complan® can be easier than eating.

It upsets me when they don't eat

It is natural to feel hurt if someone doesn't eat the food you have offered. Especially if it has taken you a long time to prepare, and you were hoping the person would eat a little to reward your effort. You may find yourself urging or nagging the person to eat. However, this can be unhelpful. It can lead to worry about mealtimes and further loss of the desire to eat. Consider what you would like someone to say to you if you felt unable to eat.

I can't help watching them eat

If you are concerned about the amount someone is eating then it is understandable that you will want to watch them eat. But, it can be troubling to have someone watch what you eat.

A solution may be to allow the person who is having eating difficulties to eat on their own, if they prefer. They can then eat what they want in their own time.

Common questions

Do lots of people have similar problems with eating?

Difficulty eating is one of the most common problems faced by people with cancer. Many family members and friends who live with someone with cancer have concerns about the person's eating habits.

continued overleaf

Further information

www.mru.nursing.soton.ac.uk/patient.html
(from November 2006)

WE ARE MACMILLAN. CANCER SUPPORT

Macmillan Cancer Support
89 Albert Embankment
London SE1 7UQ
Tel 020 7840 7840
Fax 020 7840 7841
www.macmillan.org.uk

© Macmillan Cancer Support, June 2006. Registered charity number 261017.
Isle of Man charity number 604.

© University of Southampton, March 2006. This leaflet has been prepared using evidence from many sources including research with 200 people living with advanced cancer.

MAC5844 F

MAC5884 F

LIVING WITH CHANGES IN THE SMELL OF FOOD

Information to help people living with cancer

University of Southampton | School of Nursing and Midwifery

WE ARE MACMILLAN. CANCER SUPPORT

Are you living with advanced cancer? Do you find that food smells different?

This leaflet will tell you what other people have found helpful when they were dealing with changes in their sense of smell.

Tips to help you live with changes in your sense of smell

In particular, hot dishes can smell odd or even as though the food is off. Hot drinks such as tea, coffee and chocolate can have a smell that makes you feel sick. You may find that you force yourself to eat food that smells unpleasant.

It is best to avoid food that smells unpleasant, if it spoils your enjoyment of other food.

I find the smell of food cooking unpleasant

Try to avoid the smell of cooking food. If someone else is cooking for you, you could go to a room that's away from the kitchen. Keep doors shut and windows open, or ask for the person cooking to open the windows in the kitchen.

If you are cooking for yourself, use a microwave when possible, or cook food in the oven rather than on a hob. For example, oven-bake fish instead of frying.

Perhaps there is someone who can prepare meals for you. You may need to ask, as they will probably be unaware that preparing food puts you off eating.

I don't like the smell of food on my plate

The smell of hot food is carried by rising hot air and steam as the food cools. Many cold foods have little smell. You may find that you prefer to eat your meals when they are quite cool.

Alternatively, you may find cold meals more enjoyable. Cold food is as good for you as hot food.

I find other smells put me off my food

Smells such as smoke, cleaning fluids and perfumes can put you off your meals. If you find these smells troubling, try to avoid them. Others may not be aware that smells are bothering you. You may need to explain that smells can put people with cancer off their food.

WE ARE MACMILLAN. CANCER SUPPORT

Further information

www.mru.nursing.soton.ac.uk/patient.html
(from November 2006)

WE ARE MACMILLAN. CANCER SUPPORT

Macmillan Cancer Support
89 Albert Embankment
London SE1 7UQ
Tel 020 7840 7840
Fax 020 7840 7841
www.macmillan.org.uk

© Macmillan Cancer Support, June 2006. Registered charity number 261017.
Isle of Man charity number 604.

© University of Southampton, March 2006. This leaflet has been prepared using evidence from many sources including research with 200 people living with advanced cancer.

MAC5844 H

MAC5884 H

LIVING WITH CHANGES IN THE TEXTURE OF FOOD

Information to help people living with cancer

University of Southampton | School of Nursing and Midwifery

WE ARE MACMILLAN. CANCER SUPPORT

Are you living with advanced cancer?

If so, you may find that the texture of food seems different when you eat.

This leaflet will tell you what others have found helpful when living with change in the feel of food in their mouth.

You can also talk to your nurse or doctor who will be able to assess the reason for the change in the texture of your food. The cause may be treatable.

Tips to help you live with texture change

You may find that you don't enjoy some foods any more because they have a different feel when they are in your mouth.

Perhaps meat and oranges seem 'stringy'. Or bread and potato feel mushy or gritty. Even some drinks may seem gluey.

When food has a strange texture it can mean that you have to force yourself to swallow. You may find that you have to spit the food out.

I find the feel of some foods makes me feel sick

Avoiding foods that have an unpleasant texture can help. Trying to eat foods that make you feel sick can put you off eating other things.

However, you might find that food that now has a strange texture can be eaten in a different form. For example, some people who are unable to eat bread find they can continue to eat toast.

Alternatively, consider getting your nourishment from different sorts of food. For example, eat rice instead of potato, or cheese and eggs instead of meat.

I find food dry

You may find that food seems to draw the saliva out of your mouth. When this happens it can be difficult to chew and swallow.

Soft, moist food is likely to be easiest to eat. Soft foods do not need a lot of chewing, for example, macaroni cheese or sponge pudding and custard. Sauce or gravy can help to make food soft and moist. You may also find taking sips of fluid with food helpful.

When people find some foods dry, they often still enjoy soups and cereals. You could consider increasing the energy and protein in soup by adding cream or cheese. Similarly, milk can be fortified with powdered milk before it is added to cereal, so that it contains more protein and energy. (See the *Eating well* leaflet for more information about fortifying foods.)

WE ARE MACMILLAN. CANCER SUPPORT

What can I do?

The tips in this leaflet show you some of the things other people have found helpful when living with eating difficulties.

You can also talk to your nurse or doctor who will be able to assess the reason for the change in your eating habits. The cause may be treatable.

Further information

Have you got a small appetite?
(2001) British Dietetic Association

www.mru.nursing.soton.ac.uk/patient.html
(from November 2006)

Macmillan Cancer Support
89 Albert Embankment
London SE1 7UQ
Tel 020 7840 7840
Fax 020 7840 7841
www.macmillan.org.uk

© Macmillan Cancer Support, June 2006. Registered charity number 261017.
Isle of Man charity number 604.

© University of Southampton, March 2006. This leaflet has been prepared using evidence from many sources including research with 200 people living with advanced cancer.

MAC5844 D

MAC5884 D

LIVING WITH CHANGES IN YOUR APPETITE

Information to help people living with cancer

University of Southampton | School of Nursing and Midwifery

WE ARE MACMILLAN. CANCER SUPPORT

Are you living with advanced cancer? Do you find that you are not eating as much as you used to?

If you are concerned about what you are eating, this leaflet can help you to know if you are making the most of your appetite. It tells you about some of the most common concerns and gives examples of what others have found helpful.

Tips to help you live with loss of appetite

I find eating hard work

Soft food is easiest to eat because it does not need chewing. You could try porridge, Weetabix® with milk, custards, milk puddings, stewed fruits, bananas, yogurts or milk-based drinks.

Drinking high energy and protein fluids, such as milkshakes, yogurt drinks or liquid nutritional supplements may be easier than eating.

I find my appetite changes

You may find that the amount you can eat changes from day to day for no reason. What you fancy may even change from hour to hour.

It is best to eat what you fancy. Eat on the days and at the times that you feel able to. This may not be at times you used to eat. To do this you may need to keep a stock of different tinned foods and pre-prepared frozen meals.

I feel hungry, but can't eat much

It may be helpful to have your portion served on a small plate. Perhaps have just a little of one of the foods other people are eating. If you feel full after a few mouthfuls at mealtimes, it can be a good idea to snack on nuts, crisps, toast, chocolate or biscuits between meals.

I never feel like eating anything

Try experimenting with different tastes. You may find you now like cold food or foods you would not have eaten in the past. Some people discover that although they used to prefer sweet foods they now prefer savoury, or visa versa.

I find preparing food wears me out

Using ready-made meals and accepting offers of help with cooking can make life easier. Quick food can be as good for you as a meal that takes a long time to prepare.

I worry that I upset other people when I don't eat

Listen to your body and try to explain to others what your body is telling you about eating. You can tell them that it's not their cooking, but the way you feel that is affecting your appetite.

I want to make the most of the appetite I have

Consider what your body will cope with and choose to eat or drink the things that contain most calories, protein or vitamins. Easy to eat foods that have all three include, smoothies, stewed fruit with custard, and cauliflower cheese.

Common questions

Do other people have the same problem?

Three out of four people with advanced cancer live with a change in their eating habits. Eating difficulties include loss of appetite and changes in the taste, texture and smell of food. Some cancers cause particular eating problems, such as difficulty swallowing.

Why am I eating differently?

There are many reasons why your eating habits can change. Treatments and medicines can affect how you feel about eating. Feeling sick, having diarrhoea or constipation can stop you wanting to eat. Feeling low in mood, tiredness or pain can also make eating difficult.

Even if your cancer is not causing you other problems, it can affect your eating habits. This is because your body's response to the cancer can include losing your desire to eat.

Further information

www.mru.nursing.soton.ac.uk/patient.html
(from November 2006)

Macmillan Cancer Support
89 Albert Embankment
London SE1 7UQ
Tel 020 7840 7840
Fax 020 7840 7841
www.macmillan.org.uk

© Macmillan Cancer Support, June 2006. Registered charity number 261017.
Isle of Man charity number 604.

© University of Southampton, March 2006. This leaflet has been prepared using evidence from many sources including research with 200 people living with advanced cancer.

MAC5844 G

MAC5884 G

LIVING WITH CHANGES IN YOUR SENSE OF TASTE

Information to help people living with cancer

University of Southampton | School of Nursing and Midwifery

Are you living with advanced cancer? Do you find that food tastes different?

This leaflet tells you what other people have found helpful when living with changes in their sense of taste. You can also talk to your nurse or doctor who will be able to assess the reason for the change in the taste of your food. The cause may be treatable.

Tips to help you live with taste change

You may be finding it difficult to eat because of the taste of food. Perhaps eating is no longer enjoyable because food tastes strange or your taste changes from day to day. A dry or coated tongue can lead to a change in your sense of taste.

I find food has no taste

Some people find it helpful to experiment with eating foods they have never eaten before. You might find that spicy, very sweet or highly seasoned foods are now enjoyable. It may also help to add salt, pepper or other herbs and spices to your food. Another way of living with loss of taste is to eat for texture not flavour. Rice and toast are textured foods that you may enjoy eating.

I find all food tastes strange

You may find that food tastes different. Perhaps sweet foods seem exceptionally sweet or savoury foods exceptionally salty. Why not avoid the foods that you no longer enjoy and experiment with new tastes?

You could consider different ways of preparing food. For example, try using a cooking oil spray to reduce the amount of oil on fried or roasted foods. Or maybe add salt at the table rather than during cooking.

Cold food can be just as good for you as cooked food. However, it usually has less smell and may therefore not taste as strong.

Cutlery can also change the taste of food. Some people with cancer find that they prefer plastic knives and forks.

The foods I fancy change repeatedly

You may be troubled by repeated changes in taste. What you fancy may change from hour to hour, making planning meals difficult and frustrating. It's a good idea to keep a stock of tinned and frozen foods in small portions, so that you don't have to chose what to eat in advance. Then it will be easier for you to eat different foods to your family, according to what you fancy.

I have a dry mouth

If you have a dry mouth this can change your experience of taste. It can also make eating hard work.

It is important to keep your mouth clean and moist. You may find it helpful to rinse your mouth with water or a weak salt solution (one teaspoon of salt in 500mls water), as often as every two hours. If your tongue becomes coated, brush it gently with a soft toothbrush when you clean your teeth or when you rinse your mouth. If you have dentures, take them out overnight.

To increase the amount of saliva in your mouth, try sucking sweets or chewing gum. You may find frequent sips of drink or sucking ice cubes/ice lollies soothing. Fizzy drinks can help keep your tongue free of coating.

Further information

www.mru.nursing.soton.ac.uk/patient.html
(from November 2006)

WE ARE MACMILLAN. CANCER SUPPORT

Macmillan Cancer Support
89 Albert Embankment
London SE1 7UQ
Tel 020 7840 7840
Fax 020 7840 7841
www.macmillan.org.uk

© Macmillan Cancer Support, June 2006. Registered charity number 261017.
Isle of Man charity number 604.

© University of Southampton, March 2006. This leaflet has been prepared using evidence from many sources including research with 200 people living with advanced cancer.

MAC5844 E

MAC5884 E

LIVING WITH DISAGREEMENTS ABOUT FOOD

Information to help people living with cancer

University of Southampton | School of Nursing and Midwifery

WE ARE MACMILLAN. CANCER SUPPORT

Are you living with advanced cancer? Do you find that you disagree with other people over what you should eat?

This leaflet will tell you what other people have found helpful when living with disagreements about food.

Tips to help you

Many people living with cancer find that they eat less than they used to. You might find that your smaller appetite causes you to disagree with others over what you should eat.

I don't mean to be awkward; I just can't eat very much

There are two very common misunderstandings about eating that often cause disagreements:

- others may not realise that your cancer is causing your lack of appetite and think that you are just not trying to eat
- others may try to help in ways that you find upsetting.

I find others think that I'm not trying to eat

Other family members may find it difficult to understand the change in your eating habits. They may have never lost their appetite.

It can be helpful to tell them what is happening to you. Describe the things that you think they will be able to imagine. For example, describe something as being so dry and bland it tastes like cardboard, or that you feel you will be sick if you take one more mouthful.

I get upset when others give me advice about what to eat

You may find others have ideas about what you should eat. They may encourage you to eat foods that you find unpleasant.

If you are living with advanced cancer, it is your quality of life that is important. Think about what you enjoy and what you can manage to eat. Then choose the foods or drinks that will give you energy and protein. You will then be eating well without letting food have a negative impact on your quality of life.

It puts me off my food when others watch me eat or comment on what I eat

Food is often used as a reward or a way of showing that we care. For example, we take gifts of food and drink to a party.

If someone has prepared food for you and you are unable to eat it, they may feel rejected. Try to notice the things that they do that are helpful and tell them. For example, if talking about food puts you off eating, tell your family how helpful it has been when they haven't talked about food for a time.

Another solution is to ask to eat on your own. You can then eat what you want in your own time. Some people find it best to help themselves to tinned, frozen or refrigerated food when they fancy. Friends and family might find it helpful if you make suggestions about where you would like to eat, and how you would like food served – perhaps you would like to serve your own portion too.

I don't want to hurt people's feelings when they are trying to help me

It can be difficult to talk to others about how you feel and what you would like. It may help to show them this leaflet or the leaflet called *Helping someone live with change in their eating habits*.

Further information

www.mru.nursing.soton.ac.uk/patient.html
(from November 2006)

WE ARE MACMILLAN. CANCER SUPPORT

Macmillan Cancer Support
89 Albert Embankment
London SE1 7UQ
Tel 020 7840 7840
Fax 020 7840 7841
www.macmillan.org.uk

© Macmillan Cancer Support, June 2006. Registered charity number 261017.
Isle of Man charity number 604.

© University of Southampton, March 2006. This leaflet has been prepared using evidence from many sources including research with 200 people living with advanced cancer.

MAC5844 C

MAC5884 C

MANAGING CHANGE IN YOUR EATING

Information to help people living with cancer

University of Southampton | School of Nursing and Midwifery

WE ARE MACMILLAN. CANCER SUPPORT

Are you living with advanced cancer? Are you having problems with eating?

If so, would you like some ideas on how to help yourself? This leaflet will tell you how other people have helped themselves to live with changes in their eating habits.

Tips to help you help yourself

Managing what we eat and drink is one way in which we gain a sense of control over our lives. When cancer causes a change in the desire to eat it can feel like you've lost some control.

What can I do to help myself?

It may be helpful to think of new ways of doing things that make you to feel in control of your life. Here is a list of things that others have found helpful:

- Rather than go out for a meal, you could suggest going to see an old favourite place.
- You may find you prepare more than you can eat and have leftover food to deal with. Perhaps divide meals into small portions and freeze for other occasions, or offer leftovers to family and friends.
- If you usually cook, teach another family member how to prepare meals.
- Try and be pleased with what you have eaten. It is better to feel happy about the mouthful you have eaten than troubled by thoughts about what you didn't eat.
- Tell people in advance if you plan not to eat at a social event. Also explain what you will do while they eat. Perhaps you could read, watch TV or take a nap.
- Buy yourself something new to wear. If you have lost weight, well-fitting clothes can help you feel better about your appearance.

How can I help my family understand the change in my eating habits?

Your family may have never experienced a loss of desire to eat or changes in the taste, smell or texture of their food. So they might find it difficult to understand, and try to help by urging you to eat. Try to explain what is happening to you. You could do this by describing something you are experiencing that they will be able to imagine. For example, you may feel that if you eat even a mouthful you will be sick. They might remember a time when they have eaten too much and have had similar experience.

I would like to talk to other people with similar problems

Eating problems can make you feel isolated. Many social events, such as family gatherings, include food and drink. It can be embarrassing to go along if you are unable to eat what others are eating. Also, changes in eating habits can mean you have less energy. So it can be difficult to go out and socialise.

The internet can help you get in touch with other people, without the need to leave your home. To read about others' experiences of eating difficulties go to **www.mru.nursing.soton.ac.uk/patient.html** (from November 2006)

Nutritional approaches to weight loss in cancer patients

the evidence base

Introduction

Cachexia is variably defined. The term is often used interchangeably with weight loss in patients with cancer, but this is an over-simplification of both the experience for patients and the underlying pathophysiology. Some definitions of cachexia describe percentage weight loss compared with pre-illness weight, speed of weight loss over time and / or disproportionate loss of muscle and visceral protein.[1–3] However, although cachexia is commonly recognised once patients have lost significant weight, the underlying pathology is established before weight begins to fall. The term anorexia-cachexia syndrome (ACS) encompasses the broad clinical features usually seen in patients with cancer-induced cachexia. Symptoms commonly associated with ACS include chronic nausea, early satiety (feeling full quickly) and fatigue.[4] Underlying cachexia and ACS is a complex of abnormal metabolic, neuro-endocrine and immunological pathways.

The general term weight loss is defined as a decrease in body weight resulting from voluntary or involuntary circumstances (*www.medterms.com*). This definition makes no attempt to take into consideration the clinical circumstances of a patient.

There are two important differences between cachexia and weight loss associated with reduced food intake:

- In starvation, lean muscle mass is preserved and the biggest tissue component of the weight loss is fat. In cachexia both muscle and fat are markedly depleted.
- In cachexia the degree of weight loss cannot be completely accounted for by reduced food intake. The characteristic muscle wasting occurs even in the presence of normal food intake and may be reported before any weight loss has occurred. Moreover, the weight loss of cachexia cannot be adequately treated with aggressive feeding. In contrast the weight loss associated with restricted food intake is characterised by a caloric deficiency that can be reversed with appropriate feeding.

For the remainder of this document, the term weight loss will be used to encompass all possible causes in patients with cancer.

Identification of patients at risk of nutritional problems

There are many recommendations on how to identify malnourished patients, which incorporate weight, body mass index (BMI) and recent nutritional intake.[5,6] Malnutrition is defined by the National Institute for Health and Clinical Excellence (NICE)[5] as any of the following: BMI <18.5, unintentional weight loss >10% within the preceding 3–6 months, BMI <20 plus unintentional weight loss >5% within preceding 3–6 months. However, BMI relates to chronic protein-energy status and does not reflect loss of body function resulting from recent unintentional weight loss; thus it can provide only a guide to the probability or risk of malnutrition rather than identification of malnourished individuals.

Significant unintentional weight loss suggests the presence of an underlying disease, which, if unchecked, is likely to produce further weight loss and deterioration in body function. To identify individuals on the basis of unintentional weight loss it is necessary to define the level of weight loss that is significant. Most of the proposed cut-offs include an estimation of the time frame during which the weight loss has occurred, and are not specific to cancer patients. However, one cut-off is consistent across the various recommendations: weight loss >10% occurring in the preceding six months.

Tools that screen for malnutrition include the Malnutrition Universal Screening Tool (MUST), which has been validated[6] and is cited as an example of an appropriate screening tool by NICE[5] for all patients in acute or community settings.

One tool designed specifically for cancer patients is the Patient-Generated Subjective Global Assessment (PG-SGA),[7] which relies on information about weight loss together with dietary intake, symptom profile, functional capacity and a physical examination. The original, validated version of PG-SGA is consistent with other tools in giving a weight loss of 10% or more the highest score and smaller amounts of weight loss a lower score. An abridged version is used in the Macmillan Durham Cachexia Pack, taking the first part of the document only. The questions have not been changed from the original version, and enable identification of the specific issues for individual patients and suggested strategies for management through the rest of the pack. The impact of the abridged PG-SGA, together with the management strategies detailed in *Section 4* of the pack, have been the subject of a within-group study of patients with cancer, conducted in Durham. Results are expected in early 2008.

Early identification of patients who will require intensive nutritional support as a result of their planned treatment is important. Nutritional screening and assessment for these patients is a priority.[8] Anticipating potential nutritional problems, successfully detecting them and treating them early may help the patient gain or maintain weight, improve the response to treatment and reduce complications.[9,10] Referral to a dietitian should be automatic for patients in whom nutritional problems are particularly likely, eg those with upper gastrointestinal or head and neck cancers, in accordance with the *Manual of Cancer Services* 2004.[11]

Evidence for providing nutritional intervention in patients with cancer and weight loss

The link between poor nutritional status and clinical outcome in cancer patients has been discussed in a review by Shils.[12] The article sets out the steps required to identify and manage the nutritional problems encountered by patients with cancer, and refers to cases in which early assessment of nutritional status, carried out by a physician, and subsequent management by a dietitian, proved pivotal. It highlights the importance of integrating nutritional assessment and management into the clinical care of patients, and the need for its acceptance by medical boards and teams responsible for the care of people with cancer. The Shils review was written in 1979, before any randomised controlled trials (RCTs) of nutritional intervention in patients with cancer had been undertaken, and was largely based on opinion, guided by observing the clinical effects of malnutrition on cancer patients. Nevertheless, the principles it identifies have been reiterated in numerous subsequent reviews, have been incorporated into many guidelines for nutritional practice and are considered to represent best practice in the nutritional / dietary management of patients with cancer and weight loss.

The first RCT of nutritional intervention in patients with cancer appeared in 1981.[13] Overall, to date, publications on the topic include 17 RCTs, two systematic reviews[14,15] and a set of clinical guidelines.[16] The findings have been unanimous in confirming the **lack of evidence** for a role for nutritional intervention in patients with cancer and weight loss.

A recent meta-analysis brought together data from all 17 randomised studies of nutritional intervention in patients with cancer.[17] The findings demonstrated that nutritional intervention was associated with an improvement in energy intake, but there were no differences in nutritional status, survival, tumour response and quality of life between groups receiving nutritional intervention and groups receiving no nutritional intervention. Of the 17 studies identified for the meta-analysis, 12 included data on survival. Only eight of the 17 studies were judged to be of good quality, as defined by the robustness of the randomisation process.[18] Survival results from the intervention and control arms of these eight studies were entered into a meta-analysis. There were 155 deaths in the treatment arm and 110 deaths in the control arm. The odds ratio for survival in the eight studies is 1.27 (95% confidence interval (CI)=0.86–1.89), ie the odds of survival were not significantly different if patients received nutritional intervention or no nutritional intervention.

Psychological impact of weight loss and anorexia

Despite the lack of evidence supporting nutritional intervention in people living with advanced cancers, weight loss remains one of the most common symptoms experienced by these patients.[19] Studies of nutritional interventions in people living with cancer have tended to concentrate on managing weight loss and its impact on traditional clinical outcomes, such as survival and tumour response. Little attention has been paid to the perspective of patients and how they might best be supported when managing the consequences of weight loss, anorexia and other changes to eating habits.

The prevalence of anxiety associated with anorexia has been investigated in 145 patients with advanced cancer.[20] 79% of patients had experienced some degree of anorexia, but only 36% expressed anxiety about this. Interestingly, there was inconsistency between patients and carers, with 87% of carers expressing anxiety about the patients' reduced appetite.

In another study, researchers interviewed people living with cancer and their carers about their experiences of loss of appetite.[21] A poetic transcription of the participants' words was taken to capture and communicate the essence of each person's experience. Themes that emerged included uncertainty and death, accepting limitations and what patients wanted, eg "listen to me". An example of one of the poetic transcripts is given on the following page.

An example of a poetic transcription[21]

Carer: "I don't think you realise how much it worries me, how I feel when I know you've not eaten anything. You've got to put petrol in the engine to get it to go."

Patient: "I know that you are being truthful, what you say is true. Not eating doesn't bother me. What bothers me is that it worries you. Don't be angry or feel I've let you down. It's not intentional. I know you're right, I know you're right. I'll try, I really will. But I can't. I physically can't."

A study examining the taboos about weight loss in cancer among nursing staff and carers has demonstrated that nurses often feel helpless in relation to managing weight loss and hence avoid mentioning the subject during discussions of symptoms.[22] The study also suggests that communication and a sense of connectedness are important to psychosocial well-being. One patient's frustration with the lack of effective communication over diet / nutrition is clear from a transcript recorded as part of another study from the same team (see below).[23]

From a patient interview[23]

"Sit down and talk to me properly…..Talk to me about my eating habits, rather than saying right we've got these nutritional supplements. There's things you learn….You know your own body, your likes and dislikes. So surely they can say 'Let's start from what you like'."

The issues of unmet need and patient and carer concern have also been highlighted in a study of the roles of dietitians, physiotherapists, occupational therapists and speech and language therapists in palliative care for oncology patients in the west of Scotland.[24] In needs assessment interviews with 150 palliative care patients, problems with eating or swallowing were expressed by 40 patients, of whom 23 had had no assistance and 11 reported that they wanted assistance but had not received any. In addition, of 100 patients who had poor appetite, 43 had received no assistance and 11 of these had wanted assistance.

Clearly, if a patient gains psychological benefit from nutritional support, regardless of the physical effects, this may be a valuable outcome. Nelson, for example, argues: "To deny patients improved appetite and / or food intake because it will not provide physiologic improvement ignores the very important benefit of symptom control to quality of life."[25]

Healthcare professionals are in a powerful position with regard to the impact of anorexia and cachexia on patients and their carers. It is very important to strike the right balance between acknowledging (and addressing) nutritional problems and over-emphasising the importance of nutrition. Strong encouragement (or pressure) to increase nutritional intake may increase tension and anxiety in patients with advanced disease and established ACS. In fact nutritional intake in such patients becomes irrelevant; a more pragmatic approach is needed, which includes encouraging foods and drinks that are enjoyed, assessing other barriers to eating and providing opportunities for patients to air concerns. The goal of supportive nutritional therapy, where patients wish to take this approach, is to move everyone involved (patients, carers and healthcare professionals) towards an acceptance of eating or not eating, and to reduce anxieties around eating and drinking. Healthcare professionals may be able to diminish eating-related concerns through the provision of support that helps patients to regain a balance between their eating reality and eating expectations.[23] Basic strategies to optimise nutritional intake, manage symptoms and address concerns may go some way to overcoming the sense of helplessness and hopelessness shared by patients and professionals when faced with ACS.

The remainder of this document will concentrate on the factors which can contribute to anorexia and weight loss and on the strategies available for providing nutritional help to patients with these symptoms. This section is supported by a range of leaflets included in the Macmillan Durham Cachexia Pack that deals with the various issues around eating difficulties.

Factors contributing to weight loss in cancer

1. Food intake

Taking a detailed diet history takes practice and training, but information sufficient to enable the provision of basic nutritional help can be obtained by asking about what a patient is eating each day, including snacks, and how this compares to previous eating habits. Lots of clues about the potential barriers to eating can be obtained during this part of the assessment.

2. Mechanical difficulties with eating

There are many physical changes that can result in mechanical difficulties with eating:

- odynophagia (pain on eating and swallowing)
- dysphagia (difficulty swallowing), which can range from needing to avoid some foods (but able to maintain a reasonable intake from softer / moist foods and drinks) to having difficulties with liquids
- soreness caused by lesions in the mouth
- ill-fitting dentures
- poor oral hygiene
- physical disabilities, eg a patient with arthritis of the hands may have difficulty opening food packaging.

When talking to patients about what they are able to eat it is important to ask questions about why habits have changed to try to discover any factors that limit their ability to eat.

3. Social and financial problems that affect eating

One in four of the UK population—nearly 14 million people—live in households with incomes below the European poverty line of half the average income.[26] Also, illness often affects a person's ability to work and earn money, and may lead to financial difficulties that have an impact on buying food.

Other social factors that influence food choice and eating include:

- budgeting skills
- cultural traditions
- religious beliefs
- education
- nutritional knowledge
- cooking facilities (which can vary in different types of living accommodation)
- habits
- likes and dislikes
- previous food experience
- willingness to experiment
- time available
- eating alone
- depression
- bereavement.

Talking to patients about what they eat gives insights into all aspects of the way they live, and can guide the way that nutritional help is given.

Food and eating have strong symbolic connections with survival and life, and provide one area where family members feel they can contribute to a patient's well-being and recovery. They often encourage their loved one to eat, and spend much time and effort preparing favourite foods only to be disappointed by the quantity eaten. This can lead to feelings of rejection of the caregiver's love, and feelings that the patient is not doing enough to keep well or to recover. Food then becomes a battleground, perpetuating the emotional turmoil experienced by the family and the patient. In these cases, lots of talking with both the patient and caregiver is needed to facilitate understanding and to support the patient in their choices about food.

4. Emotional difficulties

Mental function may be influenced by nutrition in several ways. It has been demonstrated that starvation and partial food deprivation in adults leads to anxiety and depression and other mental changes, which may in part be linked to specific micronutrient deficiencies.[27]

In addition, patients with cancer may be anxious and depressed about their condition, the treatments they are experiencing and about the future, which may in turn result in them eating less and feeling that they 'can't be bothered' with food. It is important to take this into consideration when giving advice about eating more, and to consider sensitive and appropriate strategies to help the patient feel better.

5. Symptoms that may reduce food intake

It is impossible to provide effective dietary advice for a patient if you do not have a clear understanding of all the symptoms that are limiting their food intake. The Macmillan Durham Cachexia Pack includes a comprehensive

assessment of symptoms as part of the nutritional assessment of a patient. Management of these symptoms takes place alongside the provision of advice on food.

The importance of asking patients about individual symptoms that might be restricting food intake is highlighted by Ottery, who states:[28] 'It is interesting to note that when doctors ask if they (the patient) are experiencing any problems eating, patients frequently respond that they are not. However, data elicited using the PG-SGA (a questionnaire completed by patients) reveal that whilst patients may not experience difficulties with chewing and swallowing per se, they may report difficulties with nausea, vomiting, diarrhoea, constipation or altered taste or smell.'

6. Pre-existing diets
A restrictive diet prescribed for a medical condition, eg hyperlipidaemia or diabetes, may limit the high-energy and high-protein foods that are necessary to prevent weight loss. Such dietary restrictions can usually be relaxed, especially if the patient is finding it difficult to maintain weight. For example, a patient with diabetes who has a poor appetite may tolerate small amounts of food throughout the day (even though they contain simple sugars) rather than following their usual diabetic meal plan.

Also, a patient may have adopted an alternative diet, believed to have a potential beneficial effect on their cancer. Such diets often require elimination of certain foods and / or intake of dietary supplements that may have side effects, and / or which interrupt meals and snacks (eg because they are taken on an empty stomach several times a day). If a patient is following an alternative dietary approach such as this, various factors should be considered and discussed:

- How does the patient feel about following the diet?
- Who prescribed it?
- Is the diet harmful (nutritionally and / or financially)?

If it is important to the patient to continue with the chosen diet, look for ways to maintain nutritional status within the guidelines. Advice from a specialist dietitian may be required.

Nutritional management of weight loss

There are no national or international guidelines on the management of weight loss associated with illness. It is generally accepted that attempts should be made to increase oral nutrient intake, but there is no clear guidance on whether this should result from increased food intake, addition of commercially available nutritional supplements or a combination of both. The British Dietetic Association (BDA) recommends that "....improving nutritional intake via ordinary foods and beverages is the first step in the process of providing nutritional support'.[29] The BDA goes on to suggest that some people may need further support in the form of sip feeds and other supplements. These recommendations are made without reference to a supportive evidence base, and researchers have demonstrated a lack of evidence for such an approach.[30] However, it is important to recognise that very few studies of dietary advice were found, and this finding may be a reflection of the absence of evidence rather than evidence of no effect.

NICE has recently examined this area and reports that significant improvements in survival, weight and incidence of complications are associated with provision of oral nutritional support (dietary advice and / or nutritional supplements) to patients with weight loss or considered to be at nutritional risk.[5] Moreover, a systematic review of oral and enteral nutritional support in patients with cancer has concluded that oral nutritional supplements are associated with improvements to dietary intake in subgroups of patients, but has failed to demonstrate any clinical benefits.[15] This analysis included only four trials in cancer patients. The findings do not change when the results from the 17 RCTs of nutritional intervention in patients with cancer are combined (see earlier, Evidence for providing nutritional intervention in patients with cancer).

Local policies may exist to guide nutritional management in weight loss and it is important to identify these.

1. Dietary advice
Advice to increase food intake has potential advantages in that it offers variety, can be tailored to individual needs and may be associated with lower costs to the health service. Provision of dietary advice to increase food intake is a core dietetic skill, but its efficacy is unknown. A recent systematic review suggests that dietary advice plus nutritional

supplements have a greater role than dietary advice alone or no intervention in the improvement of body weight and some anthropometric and functional outcomes.[30] There were insufficient data on survival to allow conclusions to be drawn, but again this does not mean that dietary advice is not effective. There were very few trials identified that examined survival, so the findings reflect absence of evidence rather than evidence of no effect.

It is uncertain whether the effects produced by dietary advice and nutritional supplements are the same, but if supplements produce clinical benefits, it is reasonable to presume that they do so by increasing nutrient intake. It then follows that if a similar increase in nutrient intake can be achieved by dietary means as by using supplements, similar clinical benefits would be expected to occur. A caveat to this is that we do not know which nutrient or combination of nutrients is responsible for the benefit (protein, energy, vitamins or trace elements) and it may not be possible to reproduce the exact changes induced by supplements using ordinary food.

The aim of advice to increase food intake is to increase the calorie and nutrient content of consumed food.

Encourage small, frequent, nourishing meals and snacks

The first way to try to increase calorie and nutrient intake is to make sure the patient is eating small amounts frequently. Assessment of the usual food intake in a 24-hour period will determine whether the patient is accustomed to having three meals a day and no snacks in between. If so, the patient can be encouraged to eat small, frequent meals and snacks. It can be helpful to suggest suitable high-calorie snacks and to talk about when to include them in the day. Remember to discuss the possibility of having a small dessert after lunch and / or dinner. The leaflet 'Focus on Food' contains lots of ideas on simple snacks to suggest—see below for an example.

Snacks equivalent to four plain biscuits (140 calories)
A fairy cake, slice of malt loaf, half a hot cross bun, half a scone and jam, medium sausage roll, packet of crisps, scoop of ice cream, slice of pizza, a cereal bar, handful of dried fruit, pot of custard or rice pudding, ham / cheese sandwich (1 slice), jam tart

It can also be useful to encourage drinks that are high in calories in place of tea, coffee and water. Milk-based drinks tend to be the most calorific: the addition of just one extra drink made with full-cream milk could increase calorie intake by up to 200 calories.

Fortify food to increase the calorie content

Fortifying is when small quantities of everyday foods such as cream, milk powder or butter are added to a dish to increase its nutritional content without increasing the portion size.

There are a number of ways of fortifying foods, but it important not to make foods so rich or unappealing that they are not eaten. Milk powder may be added to full-cream milk (5 tablespoons in 1 pint), and the fortified milk can then be used throughout the day in drinks, on cereals and in puddings. Cream, butter and cheese can be used to fortify foods such as soups, pasta sauces, cereals, mashed potato and puddings. Manufactured soup can be made more nutritious, for example by adding milk powder to tinned soup or by reconstituting the powdered variety with full-cream milk instead of water. Energy-rich foods such as sugar, honey, jam and dried fruit can be added to cereals and puddings.

See 'Focus on Food' *(Section 2)* for more ideas.

Table 1 shows how a patient's calorie intake can be altered over a day with carefully chosen additional and fortified foods.[31]

Table 1: Food choices that increase daily calorie intake

Mealtime	Standard daily food intake	Approx. calories (kcal)	Fortified / high-calorie daily food intake	Approx. calories (kcal)
Breakfast	Porridge made with water	65	Porridge made with ½ pint of full-cream (whole) milk and 2 teaspoons of sugar<br>Small glass of fruit juice	265<br>40
Lunch	Packet soup made with water<br>Bread roll<br>Banana	180<br>155<br>75	Packet soup made with full-cream (whole) milk<br>Bread roll with butter<br>Half a small banana with a scoop of ice cream	345<br>230<br>155
Evening meal	Minced meat<br>Boiled potato<br>Carrots<br>Tinned peaches in natural juice	230<br>50<br>10<br>40	Minced meat<br>Mashed potato with butter and milk<br>Carrots with butter<br>Gravy<br>Tinned peaches in syrup	230<br>70<br>45<br>35<br>55
Evening snack	2 plain biscuits	65	1 small slice of bread with butter and jam	145
Milk in tea and coffee	½ pint of semi-skimmed milk	125	½ pint of full-cream (whole) milk	165
**Total**		**995**		**1780**

2. Oral nutritional supplements

Evidence for their use

Oral nutritional supplements are commonly used to manage malnutrition. In the UK in 2004, expenditure on oral nutrition in the NHS accounted for £106 million (Department of Health). Two reviews combining results from patients with a range of clinical backgrounds have cautiously concluded that supplements may benefit survival, weight change and clinical function[32,33] but neither review addresses the role of dietary advice.[32] Also, the analysis carried out by NICE demonstrates that patients with weight loss who receive nutritional supplements have significant improvements in weight and survival and fewer complications.[5]

One of the biggest problems with routine use of nutritional supplements is whether patients can take them consistently, for long enough, to achieve the potential benefits—studies having highlighted problems with compliance.[33,34] One of the factors influencing compliance is the palatability of the supplement. For example, a study has shown that patients with cancer tend to prefer supplements based on fresh milk to those based on UHT milk or UHT fruit.[35] However this issue has not been explored adequately and cannot account for all of the taste fatigue commonly reported by patients taking supplements. More information is needed to understand the issues surrounding compliance in different clinical situations.

A qualitative study of a group of cancer patients taking nutritional supplements suggests that experiences are enormously varied (personal communication). Some people choose to follow instructions given by a health professional and take the supplements like medicine, while those at the opposite end of the spectrum choose to reject the instructions, perhaps as a way of retaining some control at a time when the daily timetable may be heavily influenced by drugs and hospital visits. Many other experiences have been identified in this study, but the most important message for health professionals is the need to listen to patients and consider the type of intervention that is most suited to helping each individual.

Using nutritional supplements

Most of the nutritional supplements marketed for the management of weight loss and disease-related malnutrition are drinks fortified with vitamins and minerals, provided in 200–300 ml cartons. They are often described as nutritionally complete, meaning that they can be used as the sole source of nutrition. In addition, there are products described as nutritionally incomplete, which means that they are not designed to be the sole source of nutrition because they do not provide all the essential nutrients required, eg fat or carbohydrate supplements that are not fortified with a complete range of vitamins and minerals.

Almost all of the published guidelines on the provision of nutritional support to people with weight loss suggest that supplements should be used only when patients have tried to increase their food intake with extra snacks and by fortifying food. This food-first policy may seem sensible, but there is no evidence in the literature to support it. Conversely, there is no evidence that a food-first policy is harmful. There is simply no good evidence on which to base practice. What we do know is that patients who receive interventions (either food or supplements) to improve their nutritional intake do significantly better than patients who do not receive additional help.[30] For some patients receiving palliative care, the benefit may be psychological, and the potential positive outcomes may lie in addressing the meaning of food and the anxieties around problems with eating.

In practice, not all dietitians follow a step-wise approach to the management of weight loss, eg some give advice on dietary modification and nutritional supplementation at the same time. However, the most important factor in making recommendations is that the advice takes into consideration what is best for the patient. For example, someone living alone and spending lots of time resting may find that sipping a nutritional supplement over the course of the day is easier to manage than shopping and preparing additional foods or seeking other practical alternatives, such as a carer providing meals, use of ready meals, etc. In contrast, if there is someone around to help prepare varied and interesting snacks, this may be a better way to encourage the patient to eat more. In addition, when considering commencing nutritional supplements, it is important to be clear of the intended outcomes and these should be explained to the patient. Commencing supplements with unattainable goals in mind should be avoided.

Choice of nutritional supplement
Choice of supplements depends partly on the nutritional needs of the person living with cancer, but mainly on the palatability and acceptability of the product. Good compliance is essential, so whether the patient likes the product is an important consideration. When giving someone an initial supply of supplements it is a good idea to provide one or two of each flavour in different product types, so patients can determine which ones they like. This approach fits well with an early review following the initial intervention, and is described in Dietary Algorithm 1 (*Section 2*).

- **Liquid supplements**

The most commonly used nutritional supplements are milk-based, ready-to-drink, nutritionally complete liquids. These are listed in the pack (*Section 2*). They are generally used in addition to food where dietary intake is compromised, eg by fatigue, swallowing difficulties or oral problems. Some patients can only manage liquids, and these drinks can be used as a sole source of nutrition provided the individual can take the required amount to meet their nutritional requirements. It is important to provide clear instructions on how to take these drinks.

Patients who find milky drinks unacceptable may prefer fruit-flavoured supplements such as Enlive Plus®, Fortijuice® and Provide Xtra®. These drinks are not intended as a sole source of nutrition, but are recommended for patients who are eating food. However, they can be used in late palliative care for patients unable to manage food or UHT milk-based supplements. It should be noted that not all fruit-flavoured products are milk-protein free.

Starter packs containing an assortment of UHT milk-based and fruit-based supplements can be useful if you are unsure about which products to give a patient and for finding out the patient's taste preferences.

Patients with established ACS often struggle to meet the recommended intake of supplement drinks and it is important to avoid coercing them and increasing anxiety and hopelessness. The focus in these patients must be on maximising comfort and minimising distress, rather than nutritional intake.

- **Powdered supplements**

There are several powdered supplements available for the management of weight loss, but they vary in nutritional content and in what they can be added to. Only Complan® Shake is suitable as a sole source of nutrition for patients who are able to eat only a limited range of foods. The other fresh milk-based powdered supplements are not intended as a sole source of nutrition and are recommended for patients who are also eating food, although they can be used in late palliative setting for patients unable to manage to eat or use UHT milk-based supplements *(see Section 2).*

Some powdered products are available from chemists and supermarkets, eg Complan® and Build Up®, but are not available on prescription and should not be confused with Complan Shake®.

Fresh milk-based supplements that are available on prescription include Complan Shake®, Scandishake®, Enshake® and Calshake® (*see Section 2*). It is worth considering prescribing fresh milk-based supplements as some patients prefer these to UHT milk-based and UHT fruit-based supplements.[35]

- **Semi-solid supplements**

Dessert-like supplements, such as Clinutren® and Forticreme Complete®, are largely used for patients with dysphagia who are at risk of aspiration. A speech and language therapist can advise on the most suitable texture modification and whether such products are safe for use.

- **Energy supplements**

Energy supplements are not suitable as the sole source of nutrition. They tend not to be the first products tried for patients with weight loss and are more likely to be suggested if other types of help have not worked. The main energy supplements are carbohydrate-containing products, eg Caloreen®, Maxijul® and Polycal®. They provide no protein and are often used when extra calories are needed with a restricted fluid intake. They can also be useful for patients who dislike other supplements or to boost energy intake for patients who are managing on a limited range of low-calorie foods. Liquid products can be diluted and used as squash or added to fruit puddings and jellies. They are high in calories but not as sweet as sugar, and can be incorporated into drinks, soups and puddings without affecting the taste.

Calogen® is a supplement containing fat and carbohydrate, and it can be taken like medicine (for example 3 x 30 ml / day). It is important that Calogen® is used to supplement a range of other foods as it is definitely not suitable as a sole source of nutrition.

Starting supplements

The amount of supplement required will vary according to how much food the patient is eating. There is no absolute rule about how much supplementation to recommend per day, but it is important not to overwhelm patients by advising unrealistic quantities. Patients are normally advised to start with two cartons or sachets per day. However, it may be more appropriate for people with established ACS to commence with just one a day, with advice to increase if they feel they can take more.

People living with cancer frequently develop taste fatigue, so, to minimise waste, it is best to prescribe small quantities initially and to wait until a patient is established on a product of their choice before prescribing larger quantities. Patients should be re-assessed frequently, and the supplement prescription reviewed (*see Section 2*).

Monitoring of nutritional supplements

There are a number of reasons for monitoring patients on nutritional supplements:

- to ensure compliance—the patient may need to try other varieties or flavours, or need recipes to support the use of supplements
- to review the nutritional objectives, which may change as the patient's disease changes; it is important to clarify and agree objectives of treatment at intervals in the disease pathway
- to consider referral to a dietitian, which is needed if the patient shows no improvement, dislikes supplements or has been using supplements for more than six months (*see Section 2*).

The precise details of monitoring need to be agreed by local nutrition support teams and may vary according to local clinical experience and the clinical progress of the patient. It is likely that the frequency of monitoring will be dictated by the number of reviews for symptom management. The parameters included in monitoring will be guided by the goals of nutritional support agreed with the patient.

NICE provides a comprehensive set of guidelines on monitoring patients on nutritional support.[5]

References

1. Bruera E *et al*. Thalidomide in patients with cachexia due to terminal cancer: preliminary report. *Annals of Oncology* 1999; **10**: 857–859.
2. Davis MP, Dickerson D. Cachexia and anorexia: cancer's covert killer. *Supportive Care in Cancer* 2000; **8**:180–187.
3. Strasser F, Bruera E. Cancer anorexia/cachexia syndrome: epidemiology, pathogenesis, and assessment. In: Ripamonti C, Bruera E, eds, Gastrointestinal symptoms in advanced cancer patients. Oxford: Oxford University Press, 2002.
4. Puccio M, Nathanson L. The cancer cachexia syndrome. *Seminars in Oncology* 1997; **24**: 277–287.
5. National Institute for Health and Clinical Excellence. Nutrition support in adults: oral supplements, enteral and parenteral feeding. Available at: http://guidance.nice.org.uk/CG32/guidance/pdf/English (accessed Aug, 2007).
6. Elia M. Screening for malnutriton: a multidisciplinary responsibility. Development and use of the 'Malnutrition Universal Screening Tool' (MUST) for adults. Redditch: BAPEN, 2003.

7. Bauer J *et al*. Use of the scored Patient-Generated Subjective Global Assessment (PG-SGA) as a nutrition assessment tool in patients with cancer. *Eur J Clin Nutr* 2002; **56**, 779–785.
8. Capra S *et al*. Cancer: impact of nutrition intervention outcome—nutrition issues for patients. *Nutrition* 2001; **17**: 769–772.
9. Langer C J *et al*. Clinical significance of weight loss in cancer patients: rationale for the use of anabolic agents in the treatment of cancer-related cachexia. *Nutrition* 2001; **17**, S1–20.
10. Strasser F. Eating-related disorders in patients with advanced cancer. *Supportive care in cancer* 2003; **11**: 11–20.
11. Department of Health. Manual of cancer services. Available at: http://www.dh.gov.uk/PolicyAndGuidance/HealthAndSocialCareTopics/Cancer/CancerArticle/fs/en?CONTENT_ID=4135595&chk=CSpbzI (accessed Aug, 2007).
12. Shils ME. Principles of nutritional therapy. *Cancer* 1979; **43**: 2093–2102.
13. Elkort RJ *et al*. Long-term nutritional support as an adjunct to chemotherapy for breast cancer. *J Parenter Enteral Nutr* 1981; **5**, 385–390.
14. Brown JK. A systematic review of the evidence on symptom management of cancer-related anorexia and cachexia. *Oncol Nurs Forum* 2002; **29**, 517–532.
15. Elia M *et al*. Enteral (oral or tube administration) nutritional support and eicosapentaenoic acid in patients with cancer: a systematic review. *Int J Oncol* 2006; **28**, 5–23.
16. Arends *et al*. ESPEN guidelines on enteral nutrition: non-surgical oncology. *Clin Nutr* 2006; **25**: 245–259.
17. Baldwin C *et al*. The efficacy of nutritional intervention in the management of weight loss in cancer: a systematic review (manuscript in preparation).
18. Schulz KF *et al*. Empirical evidence of bias. Dimensions of methodological quality associated with estimates of treatment effects in controlled trials. *JAMA* 1995; **273**: 408–412.
19. Hopkinson JB *et al*. The prevalence of concern about weight loss and change in eating habits in people with advanced cancer. *J Pain Symptom Manage* 2006; **2**, 322–331.
20. Hawkins C. Anorexia and anxiety in advanced malignancy: the relative problem. *J Hum Nutr Dietet* 2000; **13**: 113–117.
21. Souter J. Loss of appetite: a poetic exploration of cancer patients' and their carers' experiences. *Int J Pall Nursing* 2005; **11**: 524–532.
22. Hopkinson J *et al*. Exploring the experience of weight loss in people with advanced cancer. *J Adv Nurs* 2006; **54**: 304–312.
23. Hopkinson J, Corner J. Helping patients with advanced cancer live with concerns about eating: a challenge for palliative care professionals. *J Pain Symptom Manage* 2006; **31**: 293–305.
24. Findlay L *et al*. Allied Health Professional Services: an assessment of need. Available at: http://www.palliativecareglasgow.info//pdf/AHPreport.pdf (accessed Aug, 2007).
25. Nelson KA. Modern management of the cancer anorexia-cachexia syndrome. *Curr Oncol Rep* 2000; **2**: 362–368.
26. Department for Work and Pensions, Households Below Average Income 1994/95 to 2000/01, Corporate Document Services, 2002.
27. Stratton R J *et al*. Disease related malnutrition. An evidence-based approach to treatment. Oxford: CAB International, 2003.
28. Ottery FD. Supportive nutrition to prevent cachexia and improve quality of life. *Semin Oncol* 1995; **22**: 98–111.
29. Thomas B. The manual of dietetic practice. 3rd edn. Oxford: Blackwell Science Ltd, 2001.
30. Baldwin C *et al*. Dietary advice for illness-related malnutrition in adults. *Cochrane Database Syst Rev* 2007; CD002008.
31. National Prescribing Centre. Oral nutritional support (part 1). *MeRec Bulletin* 1998; **9**, 25–28.
32. Potter J *et al*. Routine protein energy supplementation in adults: systematic review. *BMJ* 1998; **317**: 495–501.
33. Peake HJ *et al*. Nutritional supplementation: how much do people drink? *Proc Nutr Soc* 1998; **57**: 94A.
34. Keele AM *et al*. Two phase randomised controlled clinical trial of postoperative oral dietary supplements in surgical patients. *Gut* 1997; **40**: 393–399.
35. Rahemtulla Z *et al*. The palatability of milk-based and non-milk-based nutritional supplements in gastrointestinal cancer and the effect of chemotherapy. *Clin Nutr* 2005; **24**: 1027–1037.

Management of skeletal muscle wasting in cancer anorexia-cachexia syndrome (ACS)

the role of exercise prescription

Introduction

The algorithms and programmes of exercise provided in the Macmillan Durham Cachexia Pack do not replace the need for specialist palliative care physiotherapy assessment and intervention. They are written for healthcare professionals who may be asked direct questions relating to exercise and activity by patients or carers, but who may not have access to specialist physiotherapy advice.

Definitions of key terms:

- endurance: "The ability to work for a prolonged time and the ability to resist fatigue"[1]
- resistance: "If resistance is applied to a muscle as it contracts, the muscle will become stronger over time"[1]
- functional activities: activities centred on key tasks
- activities of daily living (ADL): Include washing, dressing, transferring, etc
- hypertrophy: increase in muscle mass.

Effects of exercise

The beneficial effects of exercise in healthy individuals are well described in health, sports and psychology literature (see Table 1). There are a number of known effects on skeletal muscles that are trained through resistance exercise.[1] Changes occur in muscle over time in response to this training that lead to hypertrophy and an increase in mass and power. Lower intensity, higher repetition exercises may not have such a significant hypertrophic effect, but will lead to improved endurance and strength. This is the case for healthy individuals, whose diet provides them with sufficient muscle building nutrients, including carbohydrates and protein.

Goal	Method	Benefits
**Strength (increase or maintenance)**	Overload principle<br>Exercise to fatigue<br>High or low loads	Ability to perform ADL
**Endurance and cardiovascular fitness**	Moderate loads<br>Large muscle groups<br>Exercise to fatigue<br>Frequent sessions	Increased blood flow<br>Increased efficiency of heart<br>Improved recovery rate
**Mobility and flexibility**	Stretching<br>Flexibility exercises	Maintains soft tissue length<br>Maintains joint range of motion
**Relaxation**	Contract and relax programmes	Releasing tension
**Coordination and skill**	Repetition of activities	Safety<br>Functional ability

Table 1: Summary of the benefits of different types of exercise in healthy individuals.

In patients with anorexia-cachexia Syndrome (ACS), normal balances in metabolism within skeletal muscle are lost.[2] The presence of tumour factors (with concurrent deconditioning due to reduced activity levels) means that skeletal muscle loss (protein breaks down in the muscle itself) represents an abnormally high proportion of total body weight lost. Within this altered physiological context, we might ask the following questions:

- What is the primary goal of exercise in patients with ACS?
- Are there secondary objectives?
- What type of exercise programme is best to achieve these goals?
- What are the contraindications or precautions to the use of these programmes within ACS?
- What other advice should be given alongside exercise advice?
- Does research evidence support or tell us anything else about the use of exercise in ACS?

The published literature

A search of relevant medical literature was undertaken, using the online databases CancerLIT, PEDro, CINAHL, Medline, PubMed, EMBASE, Cochrane Library and ISI Proceedings. The following keywords and their synonyms were used as appropriate for each database: cancer, cachexia, exercise and physiotherapy. The search retrieved 594 results. We then further refined the search to encompass only those papers directly related to the effects of exercise on skeletal muscle in ACS.

Overall, there is a paucity of research into the effects of resistance, endurance or other exercise on the muscle bulk / strength in patients with ACS. Furthermore, there is no authoritative conclusion arising from the most directly relevant articles. However, critically, none of these articles contradicted the best practice view of palliative and oncology specialist physiotherapists as described below.

In laboratory models

Of the papers reviewed that specifically commented on the role of exercise in muscle strengthening or improved functional ability, nine were experimental studies on rats and mice. Although it is difficult to extrapolate the results to patients with ACS, these experiments are worthy of comment. In the laboratory based studies, researchers transplanted active neoplasms into rodent hosts and induced the cachexic effect. In eight studies that used either electrical stimulation to simulate exercise in specific muscles or measured spontaneous exercise, there was a hypertrophic or maintenance effect of training.[3–10] One further study noted an adverse effect of exercise on rats.[11] In this study, however, there was a vigorous, enforced exercise programme, which the authors felt may have been of an excessively high intensity and duration (it involved rats running 2 km in 100 min with an incline of 13%, three times per week). Thus it is possible to build or maintain muscle mass in the presence of ACS in mice and rats without detrimental effects, provided exercise is not excessive.

Further research is clearly required to assess the validity of these findings to people with ACS.

In people

Research has been conducted into the effects of resistance and endurance exercise in patients with chronic wasting conditions, including: HIV,[12] rheumatoid arthritis[13] and chronic heart failure.[14] Zinna *et al*[15] concluded that "patients with muscle wasting conditions and who are capable and willing to adhere to resistance and aerobic exercise training regimens can increase their muscle protein mass and strength". With regard to patients with cancer, they suggest that further research is needed in people, especially when undergoing different chemotherapy regimens. Running, treadmill walking and swimming (endurance) as part of exercise management of cancer symptoms were shown to have positive effects, including: decreased depression,[2] increased oxygen consumption, decreased nausea, increased self reported quality of life (QOL), increased oxidative capacity of muscles and increased endurance.[16] Hemming and colleagues[17] commented that exercise is better than rest in patients suffering from fatigue, reduced muscle tone or muscle mass loss, although there is no indication of what exercise means in this context.

Analysis of molecular pathways in ACS and the effect of exercise on these may give clearer indications of the most effective parameters of exercise programmes, including type, duration and intensity.[2] In a study of men receiving androgen deprivation therapy for prostate cancer, which included patients in the palliative phase, 12-week supervised resistance exercise programmes were prescribed.[18] The result was a clear improvement in muscular fitness, even among patients with more advanced disease. However, it is difficult to assess the validity of these findings for patients with other cancers and / or cachexia. The levels of resistance used (70% of one repetition maximum) far exceeded the free and body weight resisted exercises described in the best practice survey.

Schneider *et al*[19] described an exercise intervention for cancer patients at the Rocky Mountain Cancer Rehabilitation Institute. The stated goal of the institute is to "…provide scientifically based individualised exercise intervention for cancer patients…". Their programme included individualised assessments and prescription of aerobic and anaerobic exercises to be done three times a week over six months. The exact structure of programmes varied, depending on the results of initial screening and assessment and if the patient was undergoing treatment. There is no specific mention of ACS patients in the report, but the findings were positive for the following: functional capacity, muscular strength, muscular endurance, pulmonary function, decreased perception of fatigue and depression.

A distinction needs to be made between 'prescribed exercise interventions' and 'activity'. 'Activity' refers to total physical exertion while carrying out ADL and functional tasks. Whenever there were comments on 'activity' in the literature, the message was consistent and clear: activity is better than rest and should be encouraged in patients with cancer. The assumption that rest will help to increase energy levels and tolerance to activity should be challenged (in the absence of underlying clinical risk factors). Further research into the role of increasing activity levels as part of exercise prescription will depend on identification of reliable measures of physical activity.[20]

Although there is a lack of research that specifically addresses patients with ACS, overall this literature supports the notion that appropriate exercise is beneficial.

Best practice

To supplement the knowledge drawn from the literature, short interviews of best practice were carried out. Ten specialist physiotherapists in oncology / palliative care answered questions about how they prescribed exercise to the ACS client group. The data (summarised below) indicate a strong consensus of professional opinion and form the basis of Patient Exercise Programmes 1 and 2 in this pack.

What is the primary goal in the use of exercise within ACS?
Physiotherapists prescribed exercise to this client group to maintain and increase muscle strength, prevent wasting, maintain muscle bulk, give a positive focus and to maintain functional abilities (especially mobility).

Are there secondary objectives?
Physiotherapists also prescribed exercise to increase comfort, maintain soft tissue length and joint range of movement, decrease fatigue and enhance cardio-respiratory and vascular systems (conditioning).

What type of exercise programme is best to achieve these goals?
Both generalised functional exercises and targeted specific exercises were prescribed. These included walking, stair climbing and seated exercise programmes that consist of aerobic body weight resisted exercises.

Along with these programmes, the following instructions were given; "little and often", "low resistance higher repetitions", "stop if causes pain or fatigue", "start low and build intensity gradually".

What are the contraindications or precautions to the use of these programmes within ACS?
Physiotherapists were concerned about the possible presence of bony metastases, spinal cord compression, adverse fatigue and activity-induced lower limb weakness, which increases the risk of falls. They were also aware of the effects of concurrent treatment, including radiotherapy and chemotherapy, and how demoralising 'failure' is for patients with unrealistic goals.

What other advice is given alongside exercise advice?
Physiotherapists regularly gave advice on pacing of activities and energy conservation, including: respecting good days and bad days, planning ahead and not overdoing things on good days *(see patient information leaflet Pacing and Daily Activities, Section3).*

A consensus opinion

Patient exercise programmes 1 and 2 and related algorithms (see pack) were scrutinised at a meeting of 'Durham and Teesside Physiotherapy Clinical Peer Support Group, Cancer and Palliative Care' held at Teesside Hospice on July 17th 2007. Amendments were made to the layout of the programmes and a consensus was reached that they reflected current best practice for the ACS client group.

Conclusion

There is no conclusive evidence that describes the effects of resistance exercise or aerobic whole body exercise on muscle strength in patients with ACS. However, literature relating to the area of exercise and muscle strengthening, hypertrophy or maintenance of bulk in cancer patients (and patients with other chronic diseases) was reviewed. The effects of exercise on cancer-related fatigue (CRF) have been far better researched and reviewed. This literature is not described here. The author acknowledges that fatigue and muscle weakness are closely linked. This information paper should be seen alongside conclusions reached in CRF studies.

Though the interviews of specialist physiotherapists were both short and few, it is felt the resulting consensus of approach and opinion confirms the validity of the exercise programmes produced.

Psychological problems experienced by patients with cancer are often mentioned in the literature. The link between exercise, an improved perception of QOL and wellness and well being is also supported by the interviewees. Physiotherapists provide exercise programmes in the light of potential psychological benefits and in an attempt to avoid adverse psychological effects associated with unrealistic goals and expectations.

The evidence supports the view that prescribed exercise can be effective in the management of muscle wasting and other symptoms seen in patients with cachexia related to cancer.

References

1. Kisner C, Colby L. Therapeutic exercise: foundations and techniques. Philadelphia: F A Davis Company, 1990.
2. Ardies CM. Exercise, cachexia, and cancer therapy: a molecular rationale. *Nutrition Cancer* 2002; **42**: 143–157.
3. Karlberg I *et al*. Branched-chain amino-acid enriched diets and exercise prevent muscle wasting in tumor cachexia. *Surgical Forum* 1983; **34**: 437–439.
4. Baracos VE. Exercise inhibits progressive growth of the Morris hepatoma 7777 in male and female rats. *Can J Physiol Pharmacol* 1989; **67**: 864–870.
5. Daneryd PL *et al*. Effects of spontaneous physical exercise on experimental cancer anorexia and cachexia. *Eur J Cancer* 1990; **26**: 1083–1088.
6. Daneryd PL *et al*. Insulin sensitivity, hormonal levels and skeletal-muscle protein-metabolism in tumor-bearing exercising rats. *Eur J Cancer* 1995; **31**: 97–103.
7. Daneryd P *et al*. Coenzymes Q9 and Q10 in skeletal and cardiac muscle in tumour-bearing exercising rats. *Eur J Cancer* 1995; **31**: 760–765.
8. Al-Majid SSM. Effect of resistance exercise training on muscle mass and protein content in tumor-bearing mice. Madison: The University of Wisconsin, 2000.
9. Al-Majid S, McCarthy DO. Cancer-induced fatigue and skeletal muscle wasting: the role of exercise. *Biol Research Nursing* 2001; **2**: 186–197.
10. Baracos VE. Management of muscle wasting in cancer-associated cachexia—understanding gained from experimental studies. *Cancer* 2001; **92**: 1669–1677.
11. Deuster PA *et al*. Endurance exercise modifies cachexia of tumor growth in rats. *Med Sci Sports Exercise* 1985; **17**: 385–392.
12. Evans WJ *et al*. Exercise and the treatment of wasting: aging and human immunodeficiency virus infection. *Semin Onc* 1998; **25** (Suppl 6): 112–122.
13. Marcora SM *et al*. Can progressive resistance training reverse cachexia in patients with rheumatoid arthritis? Results of a pilot study. *J Rheumatol* 2005; **32**: 1031–1039.
14. Strassburg S *et al*. Muscle wasting in cardiac cachexia. *Int J Biochemistry Cell Biol* 2005; **37**: 1938–1947.
15. Zinna EM, Yarasheski KE. Exercise treatment to counteract protein wasting of chronic diseases. *Curr Opin Clin Nutrition Metabolic Care* 2003; **6**: 87–93.
16. Al-Majid S, McCarthy DO. Resistance exercise training attenuates wasting of the extensor digitorum longus muscle in mice bearing the colon-26 adenocarcinoma. *Biol Research Nursing* 2001; **2**: 155–166.
17. Hemming L, Maher D. Understanding cachexia and excessive weight loss in cancer. *Br J Community Nursing* 2005; **10**: 492–495.
18. Segal RJ *et al*. Resistance exercise in men receiving androgen deprivation therapy for prostate cancer. *J Clin Oncol* 2003; **21**: 1653–1659.
19. Schneider CM *et al*. A model program: exercise intervention for cancer rehabilitation. *Integrative Cancer Ther* 2003; **1**: 76–82.
20. Dahele M, Fearon KCH. Research methodology: cancer cachexia syndrome. *Palliative Med* 2004; **18**: 409–417.

Acknowledgement

Thank you to 'Durham and Teesside Physiotherapy Clinical Peer Support Group, Cancer and Palliative Care' for their expertise and attention.
Thank you also to the questionnaire respondents for your crucial involvement.

Management strategies for symptoms related to the anorexia-cachexia syndrome in patients with cancer

the evidence base

The medications recommended in this pack are based on the limited evidence available. Where no evidence base is present, the recommendations are based on work done in the audit and consequent research study relating to this project. It is also worth remembering that many of these recommendations are made for off-label indications or doses of medications. This is commonplace in palliative medicine; a recent audit in one palliative care unit found one in four prescriptions (affecting 66% of patients) were for medications being used off-label.[1] Therefore, many of the recommendations in this resource pack may be deemed as arguable or controversial. This is why they are meant to serve as a guide only, to be used in conjunction with the usual information sources including textbooks, for example the *Oxford Textbook of Palliative Medicine, The Palliative Care Formulary (PCF)* and websites such as www.palliativedrugs.com, www.pallcare.info and www.cancerbackup.org.uk.

This evidence base document is intended to focus on symptom management to be used in reference with *Section 4*. However, where practical information is required on the finer details of prescribing some of the more complicated medications, advice should be sought from local services such as the specialist palliative care team and pharmacy.

Introduction

Anorexia-cachexia syndrome (ACS) appears to be due to two main mechanisms—a reduction in food intake and a derangement in the body's metabolism due to factors produced either by the tumour (eg proteolysis-inducing factor) or by the body in response to the tumour (eg cytokines, including tumour-necrosis factor-α (TNFα), interleukin-1 (IL1), interleukin-6 (IL6) and others). This leads to a chronic inflammatory state, which reflects the degree and rate of weight loss.[2]

It is important to be able to recognise cachexia, identify all the symptoms that the patient is suffering from and seek to treat any reversible underlying causes. A suitable management plan can then be devised to address ongoing problems.

When devising the management plan, it is important to establish the expectations for treatment and to have realistic objectives. Neither nutritional nor drug treatments confer any survival benefit in advanced cancer, and only a minority of patients will experience weight gain. However, many of the patients' symptoms can be significantly improved.

Pharmacological management should be part of a multidisciplinary approach.Progressive cachexia causes great psychological and social distress—for example, patients and carers often believe that cachexia is due to lack of caloric intake, and counselling can reduce anxiety that the patient is starving to death. Nutritional counselling can increase intake in the majority of patients and drugs can increase the effectiveness of this.[3]

DRUGS

No appetite

Dexamethasone 4 mg mane after food, review weekly
Megestrol acetate 160 mg tds + / - ibuprofen 400 mg tds after food, review every two weeks

Corticosteroids
Corticosteroids have long been the mainstay of appetite stimulation in palliative medicine. Several double-blind randomised controlled trials have demonstrated that different types and doses of corticosteroids produce symptomatic benefit in patients with cancer cachexia.[3-6] Benefits are seen in, for example, appetite, food intake, sensation of well-being and performance status. However, the beneficial effects are limited to 2–4 weeks, so a two week review is recommended with the intention of reducing the dose and discontinuing the drug if no benefits have been demonstrated. If the patient does report benefit, the dose is continued for a further two weeks, then gradually reduced until discontinued completely. Most studies have failed to demonstrate a significant gain in body weight, consequently steroids are not recommended for weight loss alone.

Choice of corticosteroid has not been established but dexamethasone, methylprednisolone, prednisolone and hydrocortisone have been found to be effective. Optimal doses are also unclear but equivalents of prednisolone 20–40 mg have been used.[7] In the absence of specific evidence on the choice of corticosteroid and exact dose, dexamethasone 4 mg has been chosen in the Macmillan Durham Cachexia Pack to minimise the tablet burden for patients and also because of familiarity with its use at this dose in palliative medicine.[8,9]

Steroids are well tolerated for brief periods, particularly in more unwell patients and those with a poor prognosis.[7] However, the course of treatment should be monitored closely as steroids are not without problems, particularly in long-term use.

The exact mechanism of action of corticosteroids is unclear, but is thought to involve inhibition of the release of metabolic products by the tumour or the host immune system (prostaglandin). Corticosteroids also have a non-specific central euphoriant effect. In addition, there is evidence to show that steroids inhibit TNF (anti-inflammatory) action and are also involved in TNF-induced muscle proteolysis, which is characteristic of cachexia. However, experiments in animals with tumours which induce cachexia have contradicted this view, and suggest that glucocorticoids are not involved in the skeletal muscle wasting associated with cancer cachexia.[10]

Corticosteriods also have anti-emetic and analgesic properties, and can relieve compression secondary to oedema. All can be beneficial effects in patients with advanced cancer suffering from ACS.

Progestional drugs
A systematic review of megestrol acetate in the treatment of ACS analysed 26 studies.[11] Compared with placebo, megestrol acetate increased appetite in oncology patients, encouraged weight gain and improved quality of life (QOL). There were no differences between lower (<800 mg / day) and higher doses of the drug. One detailed dose-finding study, which examined doses between 160 and 1280 mg found a dose-related benefit on appetite, caloric intake, body weight gain (mostly fat) and sensation of well-being, with the optimal dose being around 800 mg. However, side effects are also dose-dependent, and the studies suggest that the starting dose of megestrol acetate could be lower—the most frequently studied dose being 480 mg / day.[12] This is why the pack recommends a dose of 160 mg tds, which can be titrated at the two week review according to the clinical response.

The exact mechanism of action of megestrol acetate is unclear. It is likely to be related to glucocorticoid and/or anabolic activity, and the drug is also thought to affect cytokine release—possibly inhibiting IL1, IL6 and / or TNFα. Megestrol acetate may also act centrally via stimulation of neuropeptide γ in the hypothalamus.[7]

Few serious adverse events have been recorded while using megestrol acetate for the treatment of ACS.[13] In trials, patients rarely need to discontinue due to side effects, which may include thromboembolism, breakthrough bleeding, oedema, hyperglycaemia, hypertension, cushingoid symptoms, alopecia and adrenal suppression. However, one study of patients with non-small-cell lung cancer showed that giving megestrol acetate might decrease survival and increase the rate of thomboembolic disease (9% with megestrol acetate versus 2% with placebo).[14]

Nonsteroidal anti-inflammatory drugs (NSAIDs)
One randomised controlled trial has compared megestrol acetate plus ibuprofen with megestrol acetate and placebo.[15] The megestrol acetate / ibuprofen group showed significant weight gain and significant improvement in quality of life scores. Both groups had increased appetite. The pack encourages consideration of the combination of megesterol acetate and ibuprofen on the basis of this evidence.

NSAIDs act to reduce mediators of inflammatory response, such as IL6. Ibuprofen has been shown to decrease resting energy expenditure and acute-phase protein production in patients with cancer. Also, studies in laboratory models have shown that NSAID-induced inhibition of prostaglandin synthesis may attenuate tumour progression and decrease cachexia.[16,17]

NSAIDs have significant side effects, including nausea, peptic ulceration, renal toxicity and exacerbation of asthma and heart failure. Many patients with ACS are frail and have coexisting morbidities, which may mean that NSAIDs are contraindicated or require careful monitoring.

Weight loss

Megestrol acetate 160 mg tds, review every two weeks (see above)
EPA supplement: Prosure (see Section 2 'Range of prescribed nutritional supplements) or Omacor 1g od to bd
Thalidomide 50 mg bd, (unlicensed medicine, risk management programme)

Thalidomide
A number of small studies have supported the use of thalidomide in patients with ACS. For example, one study of 37 patients with advanced cancer and cachexia found that those on thalidomide showed significant improvement in

appetite, nausea and sensation of well-being.[18] Further studies have confirmed benefit in patients with pancreatic or oesophageal cancer, with those on thalidomide showing either weight gain or significantly attenuated weight loss.[19,20] A dose of 50 mg bd is recommended initially, and can be titrated to effect.

Thalidomide acts to inhibit TNFα and has some complex effects on other cytokines. These actions have proved useful in the management of a range of symptoms seen in palliative care, including insomnia, sweating, nausea and neuropathic pain and add weight to the potential benefit in ACS.

Because of the risk of teratogenesis, thalidomide was withdrawn from use in the UK and is now available only on a named-patient basis via STEPS (System for Thalidomide Education and Prescribing Safely).[21] Other potential side effects include neuropathy (in which case the drug should be withdrawn immediately), drowsiness, allergic reactions, haematological effects and drug interactions.

Eicosapentanoic acid (EPA)
There is some evidence for the use of EPA in ACS. It has been shown to inhibit the production and effect of various inflammatory factors, including cytokines and proteolysis inducing factor.[22,23] In a controlled trial of 200 patients with pancreatic cancer taking supplements with or without added EPA, both groups had reduced loss of weight / lean body mass.[24] There was no overall difference in survival or QOL, but patients taking 1.5–2 EPA-supplemented cartons per day (1.1 g EPA per carton) did have a significant gain in weight and lean body mass. This group also survived longer and had higher QOL scores.

This study emphasises the importance of ensuring that the recommended dose is achieved (2 x 240 ml cartons Prosure® or 3 x 125 ml cartons FortiCare®). Omacor® contains 840 mg of EPA and docosahexaneoic acid (DHA) per 1 g capsule, and a starting range of 1–2 capsules per day is recommended. This is a subtherapeutic dose in view of the trial data, so it should be titrated to effect as side effects allow.

The most commonly encountered side effect with EPA is diarrhoea, and this is dose dependent. Other potential side effects include abnormal taste, abnormal body smell, belching and flatulence

In the trial referred to above, supplements were given for up to eight weeks, at which point benefit was seen in terms of weight gain, (lean body mass) and QOL. Review is, therefore, recommended four weekly to check patient tolerance in terms of side effects and perceived benefit in terms of weight gain and QOL.[25] If patients are not achieving the correct dose due to side effects it is advisable to try switching to a different form of EPA.

Feeling full quickly

Metoclopramide 10 mg tds before food, review within one week; fortnightly thereafter
Increase to a maximum of 120 mg / day, in divided doses

Prokinetic drugs
Feeling full quickly is thought to be due to both central and peripheral effects, although the exact pathophysiology remains unclear. Central influences may include taste changes and food aversion. Peripheral changes are thought to include poor gastric accommodation, delayed gastric emptying and / or altered sensory signals.[26] Metoclopramide is an antidopaminergic drug with a central antiemetic effect. It also has a prokinetic effect that promotes gastric emptying and is particularly effective for patients with chronic nausea due to autonomic failure and delayed gastric emptying. These problems typically respond well to metoclopramide, with significant improvement in appetite and food intake.[27–29]

Domperidone is an appropriate alternative, but metoclopramide is the most widely used prokinetic for this indication. The recommended dose of metoclopramide is 10–40 mg, taken 30 minutes before meals, or 30–120 mg as a continuous subcutaneous infusion. There is evidence to show that doses above the maximum stated in texts such as the *British National Formulary* (*BNF*) can be beneficial, and current palliative care textbooks reflect this finding.[8,30] This is why a wide dose range has been recommended, and the drug should be titrated to effect.

The main side effects of metoclopramide are extrapyramidal effects, neuroleptic malignant syndrome and occasionally drowsiness, restlessness, depression and diarrhoea. Extrapyramidal effects are rare but more common in young females and in patients left on high doses of the drug for prolonged periods of time. Therefore, it is recommended that patients taking metoclopramide are reviewed regularly and that the drug is not continued long-term where possible.

Nausea and vomiting

Metoclopramide 30–60 mg / day by injection if needed (can be given in subcutaneous syringe driver); increase dose up to 120 mg / day

Metoclopramide has been found to be particularly effective for the chronic nausea associated with ACS.[31] The drug has a short elimination half life and thus needs to be given frequently (eg 10 mg tds). If patients are vomiting, a continuous subcutaneous infusion using a syringe driver may give optimal results. There is evidence that doses above the maximum stated in texts such as the *BNF* can be beneficial, and current palliative care textbooks reflect this.[8,30] This is why a wide dose range has been recommended, and the drug should be titrated to effect.

Metoclopramide is a dopamine-receptor antagonist and a 5-HT4-receptor agonist, and in high doses it has 5-HT3 antagonist activity. It is probably useful in the nausea and vomiting caused by ACS because of its prokinetic effect (see above: 'Feeling full quickly', where the side effects of metoclopramide and dose review are also described).

Diarrhoea

Rehydration sachets, one after each loose bowel motion
Loperamide, 4 mg stat then 2 mg after each loose bowel motion (maximum 16 mg per day)

With all of these pharmacological recommendations, the initial strategy is to tackle any reversible causes. This is particularly true with diarrhoea, which may be caused by, for example, infection or laxative use. The next aim of treatment must be to replace lost fluid and make sure the patient does not become dehydrated. This can be achieved using oral rehydration salts, which enhance the absorption of water and electrolytes, replace the electrolyte deficit adequately and safely, contain an alkalinising agent to counter acidosis and are slightly hypo-osmolar, to prevent the possible induction of osmotic diarrhoea.

If patients are not improved by oral rehydration and the diarrhoea continues, is debilitating and the cause has been investigated, then loperamide is a useful drug. Loperamide is a potent mu-opioid receptor agonist, and acts almost entirely by a local effect in the gastrointestinal tract. The maximum effect may not be seen for the first 16–24 hours. The dose and use is long established and is described here in accordance with the current edition of the *BNF*.

Constipation

Senna 15 mg nocte, review within one week; 1–2 weekly thereafter
Docusate 200–400 mg / day, review within one week; 1–2 weekly thereafter
Macrogol (eg Movicol®) 1–2 sachets daily, review within one week; 1–2 weekly thereafter

It is important to try to establish the cause and nature of constipation so that the appropriate laxative can be chosen. It is worth remembering that the aim of treatment with laxatives is to make sure patients can defecate comfortably rather than at a particular interval or frequency. Separate laxatives have been recommended initially to allow titration of each one to ensure optimum stool consistency and frequency. Although laxatives can be classified according to their stimulant or softening effects, it is important to recognise that all laxatives have both effects to some degree.

For a stimulant laxative, senna is recommend at the usual dose stated in the current edition of the *BNF*. Senna stimulates the myenteric plexus to initiate peristalsis and reduce the total absorption of water and electrolytes in the colon. Its usual onset of action is after 6–12 hours, and the dose can be titrated to response. The side effects of senna are well documented, and include intestinal colic and diarrhoea. If these occur the drug should be stopped and an alternative chosen.

The softening agent docusate is recommended at a daily dose of 200–400 mg. At higher doses it becomes a stimulant laxative (as listed in the *BNF*). Docusate behaves as a detergent, emulsifying and wetting agent. It softens the stools by increasing water penetration. It also stimulates fluid secretion by both the small and large bowel. Its usual onset of action is after 12–72 hours. Where possible, docusate should be prescribed in capsule form, because the solution can produce an unpleasant aftertaste or burning sensation.

If a combination of senna and docusate have not worked, macrogols can be tried, starting with one sachet daily and with dose titratation according to response. Macrogols are osmotic laxatives, but they are administered with fluid and so do not extract further fluid from the body into the bowel. Their onset of action is after 1–2 days. Macrogols are also licensed for use in faecal impaction.[32]

Lactulose is not recommended because of its lack of efficacy and troublesome side-effects.[33]

Mouth sores

Oral candida
Fluconazole 50 mg mane, review after seven days; if candida still present continue 50 mg om for a further seven days; if still present, swab and send for culture and sensitivity analysis

Oral candidiasis is the most common oral infection in palliative care; it may be present in several different clinical forms and may spread locally. The first step in management is to treat any predisposing factors, such as dry mouth, immunosuppression and problems with dentures. Subsequent management involves treatment of the infection with an antifungal agent. Both topical and systemic preparations are available. Although the evidence for systemic over topical therapies in patients with advanced cancer is weak,[34] concordance with topical antifungal treatment regimens is worse than with the systemic agents, so a systemic agent is recommended in the pack. Systemic treatments have the added advantage of efficacy in widespread disease.

Fluconazole is suggested as the first-line treatment, based on its side effects profile. 50 mg / day is the standard dose recommended in the current edition of the *BNF*, and a course of seven days should be given, which can be extended for a further seven days if the infection persists. Because resistance to the 'azole' drugs is an increasing problem in palliative care, a swab of the oral mucosa should be sent for culture and sensitivity testing if the infection persists beyond 14 days.

The side effects of fluconazole include nausea, abdominal discomfort, diarrhoea, flatulence, headache and rash, and warrant discontinuing the treatment. Fluconazole also has some significant drug interactions, so drug combinations should be checked in the current edition of the *BNF* before starting treatment.

It is important to prevent recurrence of oral candida after treatment, which can be done successfully with good oral hygiene *(see Oral Care fact sheet, Section 4)*.

Ulcers
Adcortyl in Orabase®, apply qds, do not rub in
Corlan® pellets, apply to ulcer qds

Topical corticosteroids are the main treatment for ulcers in the oral mucosa.[35] However, they are more effective when used to prevent the development of further lesions than for treating established ulcers.[36] There is no difference in efficacy between the lozenges and paste, so the formulation should be chosen according to patient preference and ease of use. The treatment should be given for five days and then reviewed.

Occasionally, use of topical corticosteroids can cause an exacerbation of local infections and may promote the development of oral candidiasis, so the need for good oral hygiene should be emphasised.

Unknown cause
Benzydamine mouthwash 0.15% 15 ml 1–3 hourly (if it stings, dilute with warm water)

Benzydamine mouthwash has a broad spectrum of activity and is thus useful when the cause of pain is unknown. There is evidence that it is effective in a range of painful oral conditions in advanced cancer.[37] The mouthwash contains an atypical nonsteroidal anti-inflammatory drug that has analgesic, anaesthetic and antimicrobial properties.[38,39]

The main side effects of the treatment are oral numbness and stinging or burning, which can be alleviated by diluting the mouthwash with an equal volume of warm water.[37]

Dry mouth

Sugar-free sweets, chewing gum
Pilocarpine 5 mg tds, review every two weeks
Biotene Oralbalance® gel, rub on hand then apply qds and prn to mouth and lips
AS Saliva Orthana® spray, qds and prn

Dry mouth may be caused by a decrease in the secretion of saliva, an alteration in the composition of saliva or a combination of both. A variety of factors can be involved, and perhaps the most common in palliative care are irradiation of salivary glands, drug therapy (anticholinergics, antidepressants, opioids, sedatives, diuretics), dehydration, mouth breathing and oxygen therapy. In light of these possible causes, it is important to review the patient's history and medications, promote good oral hygiene, review diet and advise the patient to avoid fruit juice.

The next step is to try to stimulate saliva production, which can be achieved in several ways:

Pilocarpine is a choline ester that acts via the parasympathetic nervous supply to the salivary glands. A dose of 5–10 mg tds has been shown to improve saliva production with minimum side effects, but does require residual functioning salivary tissue.[40,41] In a randomised controlled trial comparing a mucin-based artificial saliva to pilocarpine, 76% of patients chose to continue with pilocarpine, and 90% found it produced an improvement of dry mouth.[42] The side effects of pilocarpine are usually just those of parasympathetic stimulation, ie sweating, urinary frequency, headache, flushing, nausea, dizziness, dyspepsia, fatigue. These effects appear to be dose-dependant, so a starting dose of 5 mg tds is recommended.

Chewing gum has been shown to be effective for dry mouth in a variety of patients.[43] It increases the flow of the saliva, mainly by stimulating the chemoreceptors and, to a lesser extent, the mechanoreceptors. Evidence shows that chewing gum is more effective than saliva substitutes and better tolerated by patients.[41]

If stimulating the saliva does not work or is not acceptable to patients then a saliva substitute can be used. Artificial saliva substitutes are commonly prescribed for dry mouth, and several types have been developed, differing in the lubricant used, the formulation available and the additives present. In trials patients found that mucin-based artificial saliva was more effective and better tolerated than cellulose-based artificial saliva.[44,45] It is in light of this evidence that AS Saliva Orthana® spray is recommended in the pack. It contains mucin from the gastric mucosa of pigs, thought to be less sticky and to last longer than other forms. In a separate study, patients also reported that gel-based artificial saliva was more effective than spray-based artificial saliva,[46] so Biotene Oral Balance Gel® is also recommended in this section. In practice, it has been found to be beneficial to prescribe both products and allow the patient to choose which they prefer. Also, although patients may find gel more effective and longer lasting, they find the spray more convenient when they are out.[46]

Altered taste

Solvazinc one tablet tds, review every two weeks

The most common enzyme in the membrane of the taste bud is alkaline phosphatase, for which zinc is the co-factor. Zinc, and other metals, also affect the conformation of the protein that regulates the passage of tastes through the taste bud pore.

Zinc deficiency has been linked to altered taste in several groups of patients including those with cancer.[47] Also, drugs that chelate zinc have been associated with taste disturbance. Yet the exact role of zinc in the control of taste is not fully known.[48] Many of the trials are in patients undergoing radiotherapy, so it is not clear if the data can be extrapolated for patients with taste disturbances due to other causes. A trial of zinc supplements may be helpful in patients with altered taste that remains unresolved through good oral hygiene and that is affecting their quality of life. The recommended prescription is Solvazinc® one tablet three times a day. The treatment should be undertaken as a trial, with review at two weeks, stopping if there is no apparent benefit or continuing if the treatment is helpful (with regular review). As zinc can accumulate in renal impairment it is recommended that patients have their renal function checked before initiating this therapy.

Problems swallowing

Metoclopramide 10mg tds review every two weeks

If patients report having new problems with swallowing, this must be reported to medical staff and investigated. However, if it is an existing symptom that has been investigated with no identifiable cause, a trial of metoclopramide can be considered. In some patients, problems with swallowing are linked to gastric stasis and delayed emptying of the stomach, and metoclopramide may be beneficial due to its prokinetic activity. This was found to be the case in some patients in the Durham research study. For prescription information and details of side effects and monitoring, see *Feeling full quickly, Section 5*.

References

1. Bennett M *et al*. The use of drugs beyond licence in palliative care and pain management. A position statement prepared on behalf of the Association of Palliative Medicine and the Pain Society. Southampton: APM, 2005.
2. Wilcock A. Cachexia and omega-3 polyunsaturated fatty acids: the beginning of the end or the end of the beginning. *Palliative Medicine* 2005; **19**: 500–502.
3. Bruera E *et al*. Action of oral methylprednisolone in terminal cancer patients: a prospective randomised double-blind study. *Cancer Treatment Reports* 1985; **69**: 751–754.
4. Moertel CG *et al*. Corticosteroid therapy of preterminal gastrointestinal cancer. *Cancer* 1974; **33**: 135–141.
5. Wilcox JC *et al*. Prednisolone as an appetite stimulant in patients with cancer. *BMJ* 1984; **288**: 27.
6. Della Cuna GR *et al*. Effect of methylprednisolone sodium succinate on quality of life in preterminal cancer patients: a placebo-controlled, multicenter study. The Methylprednisolone Preterminal Cancer Study Group. *Eur J Cancer Clin Oncol* 1989; **25**: 1817–1821.
7. Bruera E, Sweeney C. Pharmacological interventions in cachexia and anorexia. In: Hanks G *et al*, eds, Oxford textbook of palliative medicine. Oxford: Oxford University Press, 2004; 552–557.
8. Watson M *et al*. Oxford handbook of palliative care. Oxford: Oxford Handbooks, 2005.
9. Fearon K, Barber M. Anorexia, cachexia nutrition and fatigue. In: Hanks G, Fallon M, eds, ABC of palliative care. Oxford: BMJ Books, Plackwell Publishing Ltd, 2006; 21–24.
10. Llovera M *et al*. Muscle hypercatabolism during cancer cachexia is not reversed by the glucocorticoid receptor antagonist RU38486. *Cancer Letters* 1996; **99**: 7–14.
11. Lopez AP *et al*. Systematic review of megesterol acetate in the treatment of anorexia cachexia syndrome. *J Pain Symptom Management* 2004; **27**: 360–368.
12. Loprinzi CL *et al*. Phase III evaluation of four doses of megestrol acetate as therapy for patients with cancer anorexia and / or cachexia. *J Clin Oncol* 1993; **11**(4): 762–767.
13. Lopez AP *et al*. Systematic review of megestrol acetate in the treatment of anorexia-cachexia syndrome. *J Pain Symptom Management* 2004; **27**: 360–368.
14. Rowland KM. Randomised double blind placebo-controlled trial of cisplatin and etoposide plus megestrol acetate / placebo in extensive stage small-cell lung cancer: a North Central Cancer Treatment group study. *J Clin Oncol* 1996; **14**:135–141.
15. McMillan DC *et al*. A prospective randomised study of megesterol acetate and ibuprofen in gastrointestinal cancer patients with weight loss. *Br J Cancer* 1999; **79**: 495–500.
16. Gelin J, Andersson C, Lundholm K. Effects of indomethacin, cytokines and cyclosporine A on tumour growth and the subsequent development of cancer cachexia. *Cancer Research* 1991; **51**: 880-885
17. Tessitore L, Costelli P, Baccino FM. Pharmacological interference with tissue hypercatabolism in tumour-bearing rats. *Biochemical Journal* 1994; **299**: 71-78
18. Bruera *et al*. Thalidomide in patients with cachexia due to terminal cancer: preliminary report. *Ann Oncol* 1999; **10**: 857–859.
19. Gordon JN *et al*. Thalidomide in the treatment of cancer cachexia: a randomised placebo controlled trial. *Gut* 2005; **54**: 540–545.
20. Khan ZH *et al*. Oesophageal cancer and cachexia: the effect of short-term treatment with thalidomide on weight loss and lean body mass. *Aliment Pharmacol Ther* 2003; **17**: 677–682.
21. Peuckmann *et al*. Potential novel uses of thalidomide—focus on palliative care. *Drugs* 2000; **60**: 273–292.
22. Purasiri P *et al*. Modulation of cytokine production *in vivo* by dietary essential fatty acids in patients with colorectal cancer. *Clinical Science* 1994; **87**: 711–717.
23. Barber MD *et al*. Effect of a fish oil enriched nutritional supplement on metabolic medicators in patients with pancreatic cancer cachexia. *Nutr Cancer* 2001; **40**: 118–124.
24. Fearon KCH. Effect of a protein and energy dense n-3 fatty acid enriched oral supplement on loss of weight and lean tissue in cancer cachexia: a randomised double blind trial. *Gut* 2003; **52**: 1479–1486.

25. Freeman L *et al*. Eicosapentanoic acid (EPA) enriched nutritional supplements—what do you do with them? *Complete Nutrition* 2006; **6**: 21–23.
26. Davis MP *et al*. Early satiety in cancer patients: a common and important but underrecognized symptom. *Support Care Cancer* 2006; **14**: 693–698.
27. Shivshanker K *et al*. Tumour-associated gastric paresis: correction with metoclopramide. *Am J Surg* 1983; **145**, 221–225.
28. Bruera E *et al*. A double blind, cross over study of controlled release metoclopramide and placebo for the chronic nausea and dyspepsia of advanced cancer. *J Pain Symptom Management* 2000; **19**: 427–435.
29. Bruera E *et al*. Chronic nausea in advanced cancer patients: a retrospective assessment of a metoclopramide-based antiemetic regimen. *J Pain Symptom Management* 1996; **11**: 147–153.
30. Twycross R *et al*. Palliative care formulary, 2nd edn. Oxford: Radcliffe Medical Press Ltd, 2003.
31. Pereira J, Bruera E. Chronic nausea. In: Bruera E, Higginson I, eds, Cachexia-anorexia in cancer patients. Oxford: Oxford University Press, 1996; 23–27.
32. Culbert P *et al*. Highly effective new oral therapy for faecal impaction. *Br J Gen Pract* 1998; **48**: 1599–1600.
33. Attar A *et al*. Comparison of low dose polyethylene glycol electrolyte solution with lactulose for the treatment of chronic constipation. *Gut* 1999; **44**: 226–230.
34. Clarkson JE *et al*. Interventions for treating oral candidiasis for patients with cancer receiving treatment (Cochrane review). Oxford: Cochrane Library, 2002.
35. Ship JA. Recurrent apthous stomatitis. An update. *Oral Surg Oral Med Oral Pathol Oral Radiol Endod* 1996; **82**: 141–147.
36. Holbrook WP *et al*. Aqueous hydrocortisone mouthwash solution: clinical evaluation. Acta Odontol Scand 1988; **56**: 157–160.
37. Sonis S *et al*. Benzydamine HCL in the management of chemotherapy-induced mucositis. *J Oral Med* 1985; **40**: 67–71.
38. Turnball RS. Benzydamine hydrochloride in the management of oral inflammatory conditions. *J Can Dent Assoc* 1995; **61**: 127–134.
39. Kim JH *et al*. A clinical study of for the treatment of radiotherapy-induced mucositis of the oropharynx. *Int J Tissue React* 1985; **7**: 215–218.
40. LeVeque FG *et al*. A multicentre, randomized, double-blind, placebo-controlled, dose-titration study of oral pilocarpine for the treatment of radiation-induced xerostomia in head and neck cancer patients. *J Clin Oncol* 1993; **11**: 1124–1131.
41. Fox PC *et al*. Pilocarpine treatment of salivary gland hypofunction and dry mouth (xerostomia). *Arch Intern Med* 1991; **151**: 1149–1152.
42. Davies AN. A comparison of artificial saliva and chewing gum in the management of xerostomia in patients with advanced cancer. *Palliat Med* 2000; **14**: 197–203.
43. Davies AN. The management of xerostomia: a review. *Eur J Cancer Care* 1997; **6**: 209–214.
44. Vissink A. A clinical comparison between commercially available mucin- and CMC- containing saliva substitutes. *Int J Oral Surg* 1983; **12**: 232–238.
45. Visch LL *et al*. A double-blind crossover trial of CMC—and mucin-containing saliva substitutes. *Int J Oral Maxillofac Surg* 1986; **15**: 395–400.
46. Furumoto EK *et al*. Subjective and clinical evaluation of oral lubricants in xerostomic patients. *Sped Care Dent* 1998; **18**: 113–118.
47. DeWys WD, Walters K. Abnormalities of taste sensation in cancer patients. *Cancer* 1975; **36**: 1888–1896.
48. Ripamonti C, Fulfaro F. Taste alterations in cancer patients. *J Pain Symptom Manage* 1998; **16**: 349–351.
